# Psychosexual burden in adult genital psoriasis: outcome measurement, construct coverage, and clinical recognition gaps

**DOI:** 10.3389/fpsyt.2026.1890558

**Published:** 2026-08-12

**Authors:** Haiying Lv, Haiming Chen, Haowei Xu, Liya He, Huimei Wu, Danni Yao, Chuanjian Lu

**Affiliations:** 1 Second Clinical Medical College, Guangzhou University of Chinese Medicine, Guangzhou, China; 2 State Key Laboratory of Dampness Syndrome of Chinese Medicine, The Second Affiliated Hospital of Guangzhou University of Chinese Medicine, Guangzhou, China; 3 Department of Dermatology, The Second Affiliated Hospital of Guangzhou University of Chinese Medicine, Guangzhou, China; 4 State Key Laboratory of Traditional Chinese Medicine Syndrome, the Second Affiliated Hospital of Guangzhou University of Chinese Medicine, Guangzhou, China; 5 Guangdong–Hong Kong–Macau Joint Lab on Chinese Medicine and Immune Disease Research, Guangzhou University of Chinese Medicine, Guangzhou, China

**Keywords:** adult genital psoriasis, clinical recognition gap, construct coverage, outcome measurement, psychosexual burden, sexual function

## Abstract

Adult genital psoriasis is associated with local symptoms and effects on sexual activity, intimate relationships, body exposure, and patient communication. The associated psychosexual burden is not adequately summarized by lesion severity or sexual function scores alone. This integrative review examines outcome measurement, construct coverage, and clinical recognition of psychosexual burden in adult genital psoriasis. Existing measurement tools and related measurement approaches capture several relevant components, including genital-site severity, local symptoms, sexual activity-related impact, sexual function, quality of life, sexual health-related quality of life, genital self-image, sexual distress, and selected communication or care needs. These tools and approaches differ in measurement targets, assessment perspectives, applicable populations, scoring assumptions, and contexts of use. They therefore do not readily provide a complete representation of psychosexual burden. This review uses four candidate domains—symptom experience, sexual and intimacy-related impact, psychological and self-presentation burden, and communication and care needs—to compare construct coverage across existing tools. Sexual function scores provide information on sexual response and functional status, but do not substitute for identifying sexual distress, sexual avoidance, genital self-image, self-presentation burden, or communication needs. Even when related burden is recorded in research instruments, it does not necessarily gain clinical visibility in routine dermatology practice. To address this clinical recognition gap, this review proposes a brief, non-diagnostic, low-burden communication entry point that serves as a conceptual starting point for initial discussion, documentation, and future evaluation of psychosexual concerns in routine dermatology practice.

## 1 Introduction

Psoriasis is a chronic, immune-mediated inflammatory skin disease that can affect multiple body sites and exert sustained effects on quality of life, body image, and psychosocial functioning (1–4). Among adults with psoriasis, genital involvement is not uncommon and represents a site-specific manifestation of particular clinical relevance. Patients with genital involvement often experience impaired sexual life, sexual avoidance, and reduced quality of life (5–9). Because genital involvement directly relates to bodily exposure, intimate relationships, and sexual health, its clinical significance cannot be fully captured by the extent of involvement or by conventional severity measures alone (4, 8).

In routine dermatology practice, genital psoriasis and its associated burdens may be underreported, underrecognized, and insufficiently addressed. Some patients do not spontaneously disclose genital involvement or raise related sexual health concerns with their clinicians. Genital involvement and associated sexual health impacts are not always systematically elicited or documented during routine dermatology visits (10–13). The clinical challenge in adult genital psoriasis is not merely whether genital involvement affects sexual health, but whether these impacts can be identified and understood in routine dermatology practice.

Existing studies have used site-specific severity instruments, symptom instruments, measures of sexual impact, and quality-of-life instruments to document clinical outcomes related to genital psoriasis, including lesion characteristics, local symptoms, and impact on sexual activity (14–16). These instruments differ, however, in their measurement targets, respondents or evaluators, and contexts of use. How their findings should be interpreted collectively in relation to the sexual health burden associated with adult genital psoriasis requires closer examination.

Against this background, this review provides an integrative analysis of measurement and clinical recognition of psychosexual burden in adult genital psoriasis. The analysis focuses on four issues. First, it distinguishes the burden associated with adult genital psoriasis from sexual function or lesion-severity outcomes alone and discusses its construct boundaries as psychosexual burden. Second, it analyzes and summarizes how existing severity, symptom, sexual impact, sexual function, quality-of-life, and psychosocial tools differ in their measurement targets, assessment perspectives, and limits of applicability. Third, it uses the perspectives of construct coverage, construct underrepresentation, and construct-irrelevant variance to explain why available measurement components do not, by themselves, form a continuous representation of psychosexual burden. Fourth, it distinguishes visibility within research measurement from clinical visibility in routine dermatology practice. This distinction provides a conceptual basis for patient-centered communication, low-burden initial identification, and measurement-tool refinement research.

## 2 Literature search and evidence selection

This integrative review examined psychosexual burden associated with adult genital psoriasis, focusing on outcome measurement, construct coverage, measurement-tool applicability, and recognition in routine dermatology practice. Medline, Embase, APA PsycINFO, and CINAHL were searched from inception to June 12, 2026. The Medline search was used as the base strategy and adapted for the syntax, subject headings, and field rules of the other databases. No age or language filters were applied at the database-search stage. Language, full-text availability, adult applicability, and relevance to the review question were assessed during screening according to prespecified eligibility criteria. Details of the databases, platforms, search dates, search themes, and search strategies are provided in **Supplementary Table 1**.

The search was organized around three types of evidence. First, we identified direct evidence on adult genital, anogenital, or perianal psoriasis and related genital-site involvement. Second, we identified sources describing measurement tools, validation status, score interpretation, and applicability boundaries. Third, we identified supplementary construct, communication, and methodological evidence relevant to psychosexual burden, construct coverage, patient communication, and clinical recognition. Additional targeted searches addressed prespecified issues related to scale applicability and methodology, including the applicability of sexual function scales, patients without partners or without recent sexual activity, sexual orientation and gender identity (SOGI) and lesbian, gay, bisexual, transgender, queer or questioning, and related (LGBTQ+) representation, communication about genital psoriasis, and validity theory.

Evidence not specific to adult genital psoriasis was used only to support construct interpretation, clarify the boundaries of measurement tools, or provide methodological context. It was not treated as direct evidence of disease burden in adult genital psoriasis. Study selection was performed independently by two researchers (Haowei Xu and Liya He), according to prespecified eligibility criteria, evidence-use categories, and evidence-use boundaries. Disagreements were adjudicated by a third researcher (Danni Yao). Detailed eligibility criteria and evidence-use boundaries are provided in **Supplementary Table 2**. The search, screening, and inclusion process is shown in **Supplementary Figure 1**.

The tools and measurement pathways summarized in **Tables 1**, **2** were identified through database searching, source tracing of measurement tools, extraction of validation status and applicability boundaries, targeted supplementary searches, and manual verification. **Table 2** summarizes the relationship between each measurement tool or measurement pathway, the background clinical anchors, and the four candidate domains. Coding was performed independently by two authors (Haiying Lv and Huimei Wu) using prespecified rules. A filled circle indicated core construct coverage, a triangle indicated a construct-related signal, and a dash indicated no meaningful construct-relevant coverage. Initial agreement was reached for 78 of 80 scoring cells; the remaining two discrepancies were resolved through discussion. Detailed coding rules and decision criteria are provided in **Supplementary Material 1**.

**Table 1:** Overview of measurement tools relevant to adult genital psoriasis.

Measurement category	Representative tool(s)	Tool type	Reporting source
Genital overall severity	sPGA-G/genital PGA	ClinRO	clinician- or investigator-rated
Overall psoriasis severity reference	PASI/PGA	ClinRO	clinician- or investigator-rated
Genital area/severity scoring	mGPASI	ClinRO	clinician- or investigator-rated
Patient-perceived genital disease severity	PatGA-Genital	PRO	patient global assessment
Genital symptoms	GPSS	GenPs-specific PRO	patient-reported
Genital itch	GPI-NRS/genital itch NRS	Single-item PRO	patient-reported
Genital sexual activity frequency and limitation	GenPs-SFQ	GenPs-specific PRO	patient-reported
Genital sexual activity impact	GPSIS/sexual impact PROs	GenPs-related PRO	patient-reported
Reasons for sexual impairment	RSI	Study-specific measure	patient-reported
Generic sexual function or sexual response	FSFI/IIEF/IIEF-5/ASEX	Generic sexual function PRO	patient-reported
Psoriasis- or skin-related sexual life	QualipsoSex/SRSLQ	Psoriasis- or skin-related sexual life PRO	patient-reported
Male sexual quality of life	SQoL-M	Male sexual QoL PRO	patient-reported
Female sexual distress	FSDS/FSDS-R	Female sexual distress PRO	patient-reported
Relationship and dermatologic intimacy impact	RSS/DIS	Relationship and sexuality PRO	patient-reported
Dermatology-related QoL and sexual difficulties	DLQI/DLQI item 9	Dermatology-related QoL PRO	patient-reported
Genital self-image	FGSIS	Genital self-image PRO	patient-reported
Patient needs and treatment benefit	PBI	Dermatology PRO	patient-reported

ASEX, Arizona Sexual Experience Scale; ClinRO, clinician-reported outcome; DLQI, Dermatology Life Quality Index; DIS, Dermatologic Intimacy Scale; FGSIS, Female Genital Self-Image Scale; FSDS, Female Sexual Distress Scale; FSDS-R, Female Sexual Distress Scale-Revised; FSFI, Female Sexual Function Index; GenPs, genital psoriasis; GenPs-Itch NRS, Genital Psoriasis Itch Numeric Rating Scale; GenPs-SFQ, Genital Psoriasis Sexual Frequency Questionnaire; genital PGA, genital Physician’s Global Assessment; GPI-NRS, Genital Psoriasis Itch Numeric Rating Scale; GPSIS, Genital Psoriasis Sexual Impact Scale; GPSS, Genital Psoriasis Symptoms Scale; IIEF, International Index of Erectile Function; IIEF-5, five-item International Index of Erectile Function; mGPASI, modified Genital Psoriasis Area and Severity Index; NRS, numeric rating scale; PASI, Psoriasis Area and Severity Index; PatGA-Genital, Patient’s Global Assessment of Genital Psoriasis; PBI, Patient Benefit Index; PGA, Physician’s Global Assessment; PRO, patient-reported outcome; QoL, quality of life; RSI, Reasons for Sexual Impairment questionnaire; RSS, Relationship and Sexuality Scale; sPGA-G, static Physician’s Global Assessment of Genitalia; SQoL-M, Sexual Quality of Life Questionnaire for Men; SRSLQ, Skin-Related Sexual Life Questionnaire.

**Table 2:** Construct coverage of representative instruments and measurement pathways relevant to psychosexual burden in adult genital psoriasis.

Representative instrument or measurement pathway	Background clinical anchor: severity/local disease status	Symptom experience	Sexual and intimacy-related impact	Psychological and self-presentation burden	Communication and care needs
sPGA-G/genital PGA	•	—	—	—	—
PASI/PGA	△	—	—	—	—
mGPASI	•	—	—	—	—
PatGA-Genital	•	—	—	—	—
GPSS	—	•	△	—	—
GPI-NRS/genital itch NRS	—	•	△	—	—
GenPs-SFQ	—	—	•	—	—
GPSIS/sexual impact PROs	—	△	•	△	—
RSI	—	△	•	•	—
FSFI/IIEF/IIEF-5/ASEX	—	—	•	△	—
QualipsoSex/SRSLQ	—	—	•	•	—
SQoL-M	—	—	•	△	—
FGSIS/MGSIS	—	—	△	•	—
DLQI	—	△	△	△	—
FSDS/FSDS-R/RSS/DIS	—	—	•	•	—
PBI	—	△	△	△	•

• Indicates direct coverage; △ indicates partial or indirect coverage; — indicates no meaningful coverage. The background clinical anchor column is included only as an interpretive reference for severity or local disease status and is not considered a constituent domain of psychosexual burden. Ratings were based on the item content and intended measurement target of each instrument or pathway. Use in genital psoriasis or in a specific anatomical site was therefore not taken to indicate coverage of all psychosexual burden domains. General sexual function instruments were interpreted as measures of sexual function or sexual response, rather than as complete measures of psychosexual burden in adult genital psoriasis. Validation status, reliability, responsiveness, and cross-population applicability are summarized in **Supplementary Table 3**. Detailed scoring rules are provided in **Supplementary Material 1**. ASEX, Arizona Sexual Experience Scale; DIS, Dermatologic Intimacy Scale; DLQI, Dermatology Life Quality Index; FGSIS, Female Genital Self-Image Scale; FSDS, Female Sexual Distress Scale; FSDS-R, Female Sexual Distress Scale-Revised; FSFI, Female Sexual Function Index; GenPs, genital psoriasis; GenPs-SFQ, Genital Psoriasis Sexual Frequency Questionnaire; GPI-NRS, Genital Psoriasis Itch Numeric Rating Scale; GPSIS, Genital Psoriasis Sexual Impact Scale; GPSS, Genital Psoriasis Symptoms Scale; IIEF, International Index of Erectile Function; IIEF-5, five-item International Index of Erectile Function; MGSIS, Male Genital Self-Image Scale; mGPASI, modified Genital Psoriasis Area and Severity Index; NRS, numeric rating scale; PASI, Psoriasis Area and Severity Index; PatGA-Genital, Patient’s Global Assessment of Genital Psoriasis; PBI, Patient Benefit Index; PGA, Physician’s Global Assessment; PRO, patient-reported outcome; PROs, patient-reported outcomes; RSI, Reasons for Sexual Impairment; RSS, Relationship and Sexuality Scale; sPGA-G, static Physician’s Global Assessment of Genitalia; SQoL-M, Sexual Quality of Life Questionnaire for Men; SRSLQ, Skin-Related Sexual Life Questionnaire.

Because the included evidence differed in study design, research question, and purpose, we did not conduct a meta-analysis, formal risk-of-bias assessment, or Grading of Recommendations Assessment, Development and Evaluation (GRADE) certainty-of-evidence assessment. We instead emphasized transparency by documenting the search strategy, eligibility criteria, evidence-use categories, screening process, and **Table 2** coding procedure.

## 3 Conceptual boundaries and organizing framework

Psychosexual burden associated with adult genital psoriasis should not be reduced to impaired sexual function or directly replaced by a single sexual function score. To avoid conflating sexual function, sexual health, and psychosexual burden, this review first distinguishes their conceptual boundaries. It then examines the construct coverage, applicability boundaries, and clinical recognition implications of existing tools for adult genital psoriasis-related burden. Within this organizing framework, four candidate domains are used: symptom experience, sexual and intimacy-related impact, psychological and self-presentation burden, and communication and care needs. These domains show how existing measurement findings are distributed across different content areas and contexts of use. This dispersion is not interpreted as a deficiency of any single tool, but rather as an indication that findings from existing tools do not yet provide a continuous representation of psychosexual burden associated with adult genital psoriasis.

### 3.1 Sexual function, sexual health, and psychosexual burden

Sexual function generally refers to mental and physical functions involved in sexual response and sexual activity (17). Commonly used sexual function instruments record sexual response and functional status across domains such as desire, arousal, lubrication or erection, orgasm, pain during intercourse, and satisfaction. These instruments provide standardized information on sexual function or specific functional impairment. They are also often used as sexual health-related outcomes in psoriasis research. However, these scores primarily reflect the sexual function constructs, applicable populations, and scoring conditions defined by each instrument. They do not adequately capture psychosexual burden related to body exposure, intimate interaction, self-presentation, and patient–clinician communication.

Normal sexual function is not equivalent to sexual health. The World Health Organization defines sexual health as a state of physical, emotional, mental, and social well-being in relation to sexuality and emphasizes positive, respectful, and safe sexual experiences and relationships (18). This definition indicates that sexual health-related outcomes should not be judged solely by sexual response or functional performance. In adults with genital psoriasis, psychosexual burden is used here to denote an integrated burden associated with genital-site symptoms and lesions across the four candidate domains of symptom experience, sexual and intimacy-related impact, psychological and self-presentation burden, and communication and care needs. It is not synonymous with sexual dysfunction or sexual function scores.

Studies have shown that genital involvement is associated with reduced quality of life, impaired sexual health-related quality of life, and sexual health-related burden (7, 8, 19). Some studies suggest that sexual distress or genital-site-related burden may not always correspond fully to sexual function scores. Sexual function scores therefore cannot directly substitute for the assessment of psychosexual burden (6). In addition, willingness to expose the body in intimate relationships, anticipated pain, shame or embarrassment, and avoidance of sexual contact may all contribute to this burden (10, 20). Related distress may also remain outside routine clinical communication because of embarrassment or communication barriers (10). Therefore, this review does not treat sexual function scores as proxy measures of psychosexual burden. Instead, it regards them as measurable outcome nodes with clear interpretive boundaries. The subsequent analysis of construct coverage and the clinical recognition gap is based on this conceptual distinction.

### 3.2 Candidate domains of psychosexual burden

This review uses four candidate domains to organize existing measurement findings on psychosexual burden in adult genital psoriasis. This approach reflects the fact that existing tools were not designed around a single burden construct. Instead, they measure different content areas, including local symptoms, sexual activity-related impact, sexual function, quality of life, psychosocial burden, and communication needs. The four candidate domains are not derived from a single scale structure and do not constitute a validated measurement model. Rather, they serve as an analytical framework for comparing and interpreting these heterogeneous measurement findings.

The specification of these four domains draws primarily on distinctions in sexual health, the International Classification of Functioning, Disability and Health (ICF) and patient-reported outcome (PRO) and clinical outcome assessment (COA) frameworks regarding body functions, activities and participation, contextual factors, symptom impact, functional impact, and patient experience (18, 21–25). Relevant research also suggests that sexual and intimacy-related burden in genital psoriasis and other dermatologic diseases may involve local symptoms, sexual activity and intimate relationships, shame or stigma, self-presentation, and communication barriers (26, 27). Accordingly, this review uses these four candidate domains primarily to illustrate which content areas are covered by existing measurement findings, which areas remain fragmented or underrepresented, and what coverage boundaries exist when existing instruments are used to represent psychosexual burden in adult genital psoriasis.

Genital-site lesion severity and local disease status provide important clinical context for understanding related burden, but they should not be directly equated with psychosexual burden itself. Site-specific severity instruments and local disease assessments record genital-site lesion presentation, disease severity, or treatment response (14). These indicators help clarify the disease basis for symptoms and burden. However, they do not directly represent the burden that patients experience in intimate relationships, sexual contexts, self-presentation, and patient–clinician communication.

Symptom experience primarily refers to patients’ subjective experience of local symptoms associated with genital or perianal psoriasis, including itching, pain, burning sensations, friction-related discomfort, and discomfort related to sexual activity. Such local symptoms can be recorded using symptom-specific instruments, such as the Genital Psoriasis Symptoms Scale (GPSS) and related pruritus numeric rating scales (15). In the analysis of psychosexual burden, the relevance of symptoms lies not only in their intensity or frequency. It also concerns whether these symptoms become salient during body exposure, intimate contact, friction, or sexual activity, and whether they further influence patients’ behavioral choices and subjective burden.

Sexual and intimacy-related impact primarily refers to the effects of genital or perianal psoriasis on participation in sexual activity, intimate contact, and sex-related distress, including reduced frequency of sexual activity, limitations in intimate activities, avoidance of sexual contact and concerns about symptom worsening. Related tools record sexual activity frequency or limitations in sexual activity, such as the Genital Psoriasis Sexual Frequency Questionnaire (GenPs-SFQ) (16). This domain may be related to changes in sexual function, but it does not presuppose sexual dysfunction. Avoidance of sexual contact should also not be treated simply as a secondary manifestation of sexual dysfunction. It may reflect anticipated pain, shame, concerns about symptom worsening, or self-protection in intimate contexts (20).

Psychological and self-presentation burden primarily refers to shame, embarrassment, exposure anxiety, concerns about body or genital self-image, stigma, and concerns about partner evaluation arising in contexts of body exposure, intimate contact, partner evaluation, or disease misconceptions. The impact of genital psoriasis is not limited to symptoms or sexual function. It also involves psychological stress related to body exposure, intimate relationships, and disease communication (10). This domain helps explain why some burdens may not be fully captured by routine severity measures or a single sexual function score.

Communication and care needs mainly concern whether patients are willing or able to raise genital involvement and related psychosexual concerns. They also concern whether these issues are identified, documented, and linked to appropriate follow-up assessment or care when needed in routine dermatology practice. Symptoms involving genital sites and sexual health-related concerns are often difficult to discuss because of embarrassment or shame. Therefore, even when patients can report related impacts in research questionnaires, these concerns do not necessarily enter patient–clinician communication, systematic inquiry, or clinical documentation during routine dermatology care (10, 28, 29).

### 3.3 Contexts of use and interpretive boundaries

A measurement instrument does not serve the same function across contexts. The interpretation and use of patient-reported outcomes require attention to the intended purpose, target population, and context of use (24). Their interpretation should not be generalized beyond that context without caution. In research, measurement tools typically serve as standardized outcomes for comparisons across populations, time points, or treatment responses. In clinical assessment, they may support evaluation of disease severity, local symptom burden, sexual activity-related impact, or specific functional impairment. In routine dermatology practice, however, the focus is not necessarily on administering a complete scale assessment. Rather, it is on determining whether genital involvement and its associated psychosexual burden warrant further inquiry, documentation, or evaluation.

This distinction in context of use is particularly important for adult genital psoriasis. Instruments used as research outcomes improve standardization and comparability, but they are not necessarily suitable for initial identification in routine dermatology practice. Instruments that record sexual function, sexual activity frequency, or limitations in sexual activity also do not necessarily capture shame, concerns about body exposure, self-presentation burden, or communication needs. Research measurement, individual clinical assessment, and recognition in routine dermatology practice should therefore be understood as related but functionally distinct components.

## 4 Existing measurement instruments: landscape and limits

Outcome measurement for adult genital psoriasis-related burden is not absent. Rather, it is dispersed across existing tools for clinical severity, symptoms, sexual impact, sexual function, quality of life, and psychosocial burden. Different tools provide information on disease severity, local symptoms, sexual impact, sexual function, quality of life, and psychosocial context. However, they differ in measurement focus, assessment perspective, context of use, and interpretive boundaries. This section does not aim to provide an exhaustive list of all scales. Instead, it summarizes the construct coverage of different tool categories and their applicability boundaries when used to interpret psychosexual burden in adult genital psoriasis. Representative tools and measurement approaches are shown in **Table 1**. The validation status and applicability boundaries of each tool are provided in **Supplementary Table 3**.

### 4.1 Main types of existing measurement instruments

#### 4.1.1 Lesion and symptom assessment instruments

Assessment of lesion severity is a foundational component of the measurement landscape for adult genital psoriasis. Whole-body severity indices such as the Psoriasis Area and Severity Index (PASI) can still serve as references for overall psoriasis disease activity, but they are not designed to assess the genital area specifically and have limited ability to capture the site-specific impact of sensitive-site involvement. Among site-specific instruments, the static Physician’s Global Assessment of Genitalia (sPGA-G) is a representative measure that enables standardized assessment of genital lesion severity, allowing genital involvement to be evaluated as a distinct target of assessment in clinical research and treatment-response evaluation (14). Other approaches to assessing genital severity include the Patient’s Global Assessment of Genital Psoriasis (PatGA-Genital), which evaluates the overall severity of genital involvement from the patient perspective, and the modified Genital Psoriasis Area and Severity Index (mGPASI), which provides a complementary lesion-based assessment of genital severity.

Several clinical studies of genital psoriasis have used the sPGA-G, modified genital PGA response, or similar genital severity indicators to define study populations, evaluate treatment response, and track disease changes (30–35). These instruments and related measurement pathways provide complementary information on genital severity from both patient-reported and clinician- or investigator-rated perspectives (33, 34, 36), but they should be distinguished from generic severity measures such as PASI and PGA, as well as from sexual function scales.

Symptom-oriented instruments more closely reflect patients’ subjective experience. The GPSS records patient-reported local symptoms associated with adult genital psoriasis in a structured way, including itching, pain, burning sensations, erythema, and scaling (15). Studies have also used the genital psoriasis itch Numeric Rating Scale (GPI-NRS) or similar single-symptom ratings to record the intensity of individual symptoms such as genital itch and their changes with treatment (37–39). Such single-symptom ratings primarily capture the intensity of a specific symptom and should not be equated with full multi-item symptom scales.

Overall, lesion- and symptom-oriented instruments are valuable for translating genital-site lesions and local symptoms into clinical outcomes that can be recorded and compared. However, their measurement focus remains on lesion presentation and local symptom intensity. On their own, they do not explain the meaning of these symptoms in intimate or sexual contexts. They are therefore necessary components of the measurement landscape, but they do not form an integrated assessment pathway for psychosexual burden.

#### 4.1.2 Measures of sexual activity impact and sexual function

Measures of sexual activity impact extend assessment beyond local lesions and symptoms to include participation in sexual activity, sexual frequency, and related limitations. In adult genital psoriasis, the GenPs-SFQ is a representative site-specific instrument used to assess recent sexual activity frequency and the degree to which genital psoriasis restricts it (16).

In addition to the GenPs-SFQ, other patient-reported instruments or endpoints have been used to record sexual impact related to genital psoriasis, such as the Genital Psoriasis Sexual Impact Scale (GPSIS). These instruments or endpoints typically focus on avoidance of sexual activity, the effects of sexual activity on symptoms, or the impact of genital involvement on participation in sexual activity. Clinical studies have also incorporated genital symptoms and their impact on sexual activity into outcome frameworks, indicating that genital-specific sexual impact has become a quantifiable research target (37, 38).

Generic sexual function or sexual response instruments, including the Female Sexual Function Index (FSFI), the International Index of Erectile Function (IIEF) and its short form, the IIEF-5, and the Arizona Sexual Experience Scale (ASEX), can serve as general sexual function comparators. These instruments assess domains such as desire, arousal, lubrication or erection, orgasm, pain during intercourse, and satisfaction (40–43). They are appropriate for evaluating general sexual function, but they do not directly cover local symptoms specific to adult genital psoriasis, intimate contexts, sexual avoidance, or self-presentation burden. Thus, although these instruments provide information on sexual response and functional performance, they should be interpreted as measures of general sexual function rather than measures of genital psoriasis-specific psychosexual burden. The interpretation of these sexual function instruments needs to account for item assumptions and target populations. Some sexual function instruments include item content or scoring approaches that may assume recent sexual activity, a partner relationship, or specific forms of sexual activity. For patients without recent sexual activity or a partner, or whose forms of sexual activity do not match item assumptions, scores may be affected by inapplicable items, missing responses, zero-score handling, or interpretive boundaries. Interpretation of IIEF scores may be influenced by recent sexual activity and relationship status (44). The applicability of the FSFI in sexual minority women and in populations without a partner or without recent sexual activity also requires cautious interpretation (45). Cognitive interviewing and applicability studies of the FSFI further suggest that some items may embed assumptions about partner relationships, current sexual activity, heterosexual activity, or penetrative sexual activity. Its use across groups differing in sexual orientation, partner status, recent sexual activity, and forms of sexual activity therefore requires attention to applicability boundaries (46).

Overall, the measurement tools described above provide partial but useful information for understanding psychosexual burden in adult genital psoriasis, but their measurement targets and interpretive boundaries differ. Therefore, when discussing psychosexual burden, sexual activity-related impact and sexual function scores should be interpreted according to their specific contexts of use. They should not be treated as direct proxy measures of overall psychosexual burden.

#### 4.1.3 Quality-of-life and psychosocial instruments

Generic quality-of-life and psychosocial instruments provide information on overall burden and broader context. The Dermatology Life Quality Index (DLQI) is widely used in psoriasis research and captures the overall impact of skin disease on daily life (47). Because item 9 of the DLQI addresses sexual difficulties, it may provide a brief signal related to sexual quality of life. However, a single item cannot distinguish among contributing sources such as local symptoms, sexual avoidance, self-image concerns, intimate-relationship factors, or care needs. The GENIPSO study reported that genital involvement was associated with impairments in general quality of life and sexual quality of life, suggesting that generic quality-of-life frameworks can partially reflect the overall burden in patients with genital involvement (7).

In addition to generic quality-of-life instruments, QualipsoSex and the Skin-Related Sexual Life Questionnaire (SRSLQ) extend the measurement of psoriasis-related sexuality and skin-related sexual life. QualipsoSex assesses the impact of psoriasis on patients’ sexual experience and sexual quality of life, with content that may address sexual attractiveness, initiative in intimate or sexual contexts, the impact of genital involvement on sexual life, and perceived lack of physician attention to sexual quality of life (48, 49). The SRSLQ provides a broader approach to measuring the impact of skin disease on sexual life, with item themes that may include overall impact on sexual life, social and sexual avoidance, and body image (50). Although neither instrument is specific to adult genital psoriasis, both can serve as non-site-specific supplementary references for understanding constructs such as skin disease-related sexuality, sexual quality of life, and avoidance behavior.

Instruments that assess sexual distress, intimate relationships, and sexual health-related quality of life further indicate that measurement of psychosexual burden cannot rely solely on lesion, symptom, or sexual function scores. The Female Sexual Distress Scale (FSDS), the Female Sexual Distress Scale–Revised (FSDS-R), Sexual Quality of Life Questionnaire for Men (SQoL-M), Relationship and Sexuality Scale (RSS), Dermatologic Intimacy Scale (DIS), and research pathways related to reasons for sexual impairment (RSI) provide complementary reference points from the perspectives of sexual distress, sexual quality of life, relationship burden, intimacy-related impact, and reasons for sexual impairment, respectively (5, 6, 19, 51–53). Patient Benefit Index (PBI) provides an additional measurement reference for communication and care needs from the perspectives of patient needs and treatment benefits (28, 54, 55). However, these instruments or pathways are mostly general, sex-specific, skin-related, treatment-benefit, or study-specific comparators rather than adult genital psoriasis-specific psychosexual burden instruments. Their main value is therefore to indicate which psychosocial constructs are already recordable within the existing measurement landscape and which still rely on indirect reference points.

Genital self-image is an often overlooked component of psychological and self-presentation burden and is directly relevant to genital involvement. Existing evidence suggests that this construct is measurable, although its measurement coverage in the adult genital psoriasis literature remains uneven. The Female Genital Self-Image Scale (FGSIS) quantifies female genital self-image and includes content on genital self-evaluation, satisfaction with genital appearance, comfort with exposure in partner contexts, perceived function, and shame or embarrassment (56). In women with psoriasis, genital involvement has also been reported to be associated with impaired genital self-image, increased sexual distress, and poorer sexual health-related indicators (59). By comparison, general measurement references for men include the Male Genital Self-Image Scale (MGSIS) (57). Research in men with psoriasis suggests that psoriasis and genital involvement may affect sexuality, sexual satisfaction, or erectile function (58), although this evidence is more indirect. Overall, these findings support including genital self-image in the discussion of psychological and self-presentation burden and indicate asymmetric coverage of this construct across sex groups.

Quality-of-life and psychosocial instruments provide information on sexual quality of life, self-image, shame, stigma, intimate relationships, and psychosocial context. However, their measurement targets usually concern overall quality of life, skin disease-related sexual impact, or psychosocial circumstances, rather than adult genital psoriasis-specific psychosexual burden itself. These instruments therefore help expand the construct visibility of psychosexual burden, but they do not directly constitute an integrated assessment of psychosexual burden in adult genital psoriasis.

### 4.2 Measurement boundaries and construct coverage

Taken together, existing instruments provide several measured components of adult genital psoriasis-related burden, including lesion severity, local symptoms, sexual activity-related impact, sexual function, sexual health-related quality of life, and selected psychosocial constructs. Representative instruments are shown in **Table 1**; construct coverage relationships between these instruments and the candidate domains are shown in **Table 2**. However, these instruments differ in measurement targets, assessment perspectives, validation status, and applicability boundaries. Some genital psoriasis-specific instruments have been developed or used in clinical trial settings, whereas others are single-symptom scores or measurement approaches with relatively limited validation evidence. The evidence base for content development, reliability, responsiveness, and cross-population applicability also differs across instruments. Therefore, this review treats these instruments as heterogeneous measurement sources, not as psychometrically equivalent tools. It also does not interpret them as a complete, unified assessment system for psychosexual burden associated with adult genital psoriasis.

This review uses the four candidate domains to compare construct coverage across existing instruments. The purpose is to clarify which content areas have been measured more fully, which still depend mainly on indirect signals, single items, or supplementary evidence, and to provide a basis for the subsequent discussion of construct underrepresentation, construct-irrelevant variance, and the clinical recognition gap.

## 5 Construct misalignment and the clinical recognition gap

**Figure 1:**
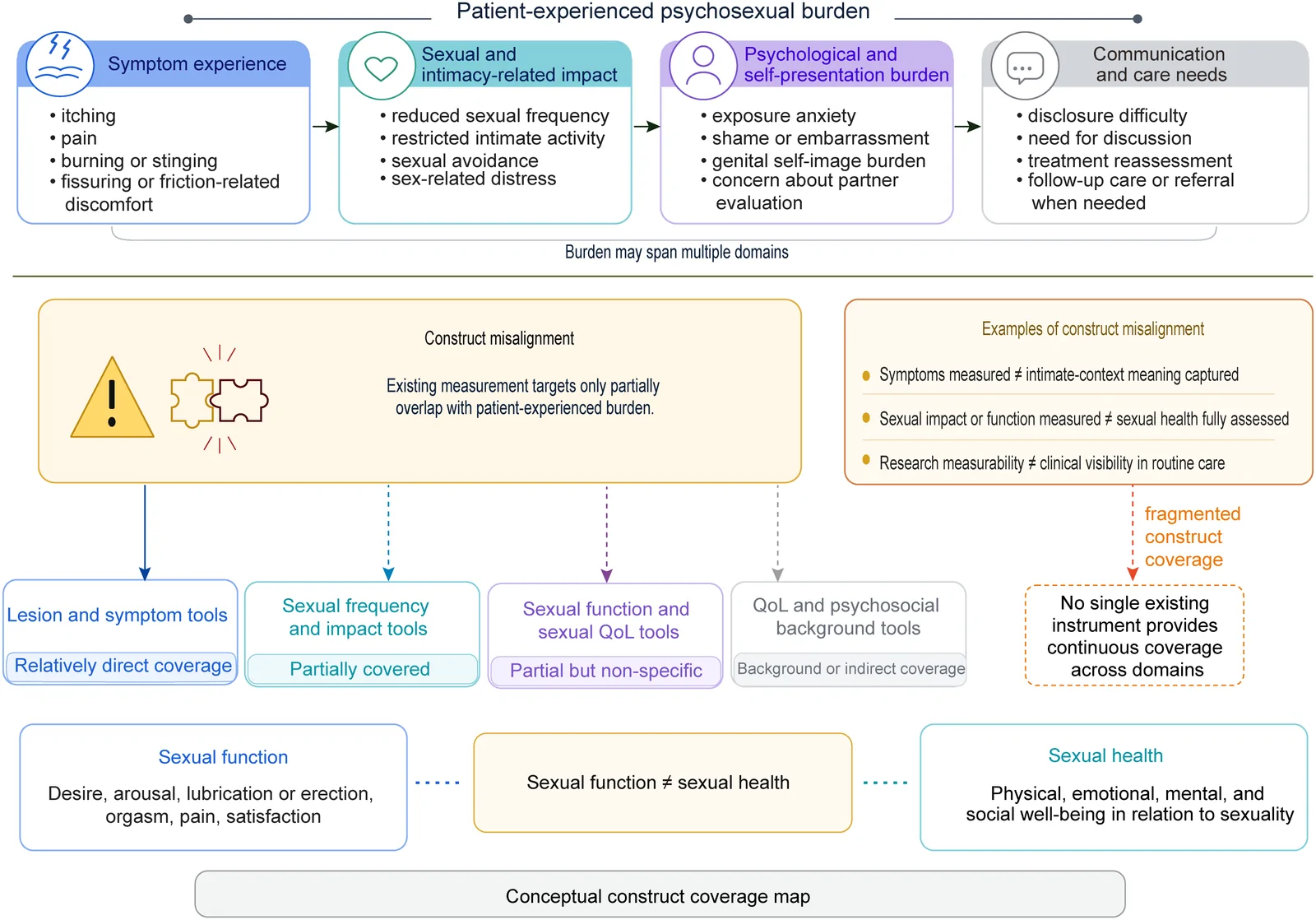
Conceptual construct coverage map of psychosexual burden in adult genital psoriasis. This conceptual map illustrates how patient-experienced psychosexual burden in adult genital psoriasis may span four candidate domains: symptom experience, sexual and intimacy-related impact, psychological and self-presentation burden, communication and care needs. Existing measurement targets provide partial and fragmented construct coverage across lesion and symptom tools, sexual frequency and impact tools, sexual function and sexual quality-of-life tools, and quality-of-life or psychosocial background tools. The figure illustrates construct misalignment through three examples: measured symptoms may not capture intimate-context meaning; sexual impact or function may not fully assess sexual health; and research measurability may not translate into clinical visibility in routine dermatology practice. The figure also distinguishes sexual function from sexual health. Sexual function comparators assess desire, arousal, lubrication or erection, orgasm, pain, and satisfaction. Sexual health encompasses physical, emotional, mental, and social well-being in relation to sexuality. Coverage labels indicate conceptual proximity to the candidate domains and do not indicate validation status, psychometric quality, or instrument ranking. QoL, quality of life.

This review defines construct misalignment as an incomplete correspondence between the measurement targets, item content, and score interpretation of existing instruments and the psychosexual burden actually experienced by adults with genital psoriasis. Existing instruments record several important outcomes in adult genital psoriasis, but these outcomes do not fully correspond to that burden. Specifically, lesion or symptom scores mainly represent disease status and local symptoms. They do not necessarily capture the meaning of symptoms during intimate contact, body exposure, or partner interaction. Sexual activity-related impact or sexual function indicators record sexual participation, sexual response, or functional limitation, but they do not necessarily cover sexual distress, sexual avoidance, genital self-image, self-presentation burden, or care needs. Burden recorded in research questionnaires or interviews also does not necessarily enter proactive inquiry, systematic documentation, or follow-up care in routine dermatology practice. Existing studies on adult genital psoriasis and related supplementary construct evidence suggest that genital involvement may be associated with reduced quality of life, sexual health-related burden, sexual avoidance, burden related to sexual life, body exposure-related stress, shame or stigma, impaired genital self-image, and communication difficulties (5, 6, 10, 13, 19, 20, 59). Therefore, the core focus of this review is the correspondence between existing measurement content and these construct nodes, together with the interpretive boundaries of that correspondence. These construct coverage relationships and key forms of construct misalignment are illustrated in **Figure 1**.

This misalignment can be further understood through two validity-related issues: construct underrepresentation and construct-irrelevant variance (22, 24, 60). Construct underrepresentation refers to insufficient coverage of important content within the target construct, such as sexual avoidance, body exposure-related stress, genital self-image, self-presentation burden, communication difficulties, and care needs. Construct-irrelevant variance refers to the influence of factors outside the target construct on instrument scores or score interpretation, such as partner status, recent sexual activity, scoring rules, age, cultural context, forms of sexual activity, and sexual orientation.

Therefore, this review uses the four candidate organizing domains as analytical threads to organize and synthesize existing heterogeneous evidence. Building on this framework, it further examines the construct boundaries and misalignment among local symptoms, sexual function measurement, and clinical recognition.

### 5.1 From symptoms to psychosexual burden

Psychosexual burden in adult genital psoriasis does not necessarily begin with sexual dysfunction. When local itching, pain, friction-related discomfort, or appearance changes involve genital sites, these symptoms or changes may become salient in intimate contexts involving body exposure, touch, sexual intercourse, partner evaluation, and self-presentation. Patient experience may then extend beyond symptom intensity to include anticipated pain, concerns about symptom worsening, embarrassment, shame, concerns about rejection, and anticipatory avoidance. Existing studies suggest that genital involvement is associated with reduced quality of life, sexual health-related burden, sexual avoidance, and burden related to sexual life. It may also involve body exposure-related stress, shame, stigma, and concerns about partner interaction (7, 8, 10, 19, 20). Recent routine-care studies from Asia and Germany further support associations between genital involvement and quality of life, sexual health-related outcomes, or burden related to sexual life, and suggest that more proactive clinical assessment may be needed in routine dermatology practice (5, 13).

On this basis, psychosexual burden in adult genital psoriasis should also be understood in relation to body image, shame, stigma, and sexuality-related cognitions. Existing studies suggest that avoidance related to genital psoriasis may be associated with pain, shame, or concerns about rejection, rather than only with sexual response or functional performance (5, 20). Research in women with psoriasis also suggests that genital involvement may be associated with impaired genital self-image, sexual distress, and poorer sexual health-related indicators (59). Broader evidence from psoriasis and chronic skin diseases suggests that lesion location, body dissatisfaction, shame, stigma, coping style, and social support may influence patients’ self-perception and avoidance behavior in contexts involving body exposure, sensitive sites, and intimate interaction (1–3, 61–63). This evidence does not replace direct evidence on adult genital psoriasis, but it supports including body evaluation, shame, self-presentation, and social interaction-related stress within the interpretive scope of psychosexual burden.

Therefore, psychosexual burden associated with adult genital psoriasis should not be understood as a simple sum of lesion severity, symptom, sexual function, or quality-of-life scores. Local symptoms provide information on disease status and physical discomfort. However, understanding the burden that patients actually experience also requires attention to intimate contact, body exposure, partner interaction, self-presentation, body image, and experiences of stigma. Recording symptom intensity or sexual function performance alone may not adequately capture psychosexual burden in these contexts.

### 5.2 Sexual function scores and psychosexual burden

Sexual function measures are important measurement nodes in research on psoriasis−related burden. Instruments such as the FSFI, IIEF, and ASEX record sexual response and functional status, including sexual desire, orgasm, pain during intercourse, and satisfaction (40–43). They have also been used to quantify sexual function impairment and related treatment outcomes in patients with psoriasis (64–66). However, sexual function scores mainly reflect sexual function or sexual response. They are not equivalent to sexual distress, sexual avoidance, self−image burden, or intimate relationship−related stress. A network analysis study also suggests that psychological symptoms, sexual function, and sexual distress are related but distinct constructs (67). Studies of adult genital psoriasis and related evidence suggest that genital involvement may be accompanied by sexual distress, sexual avoidance, limitations in sexual life, and impaired genital self−image. These burden components are related to sexual function, but sexual function scores alone may not fully capture them (5, 19, 20, 59).

Evidence on impaired body image, shame, stigma experiences, and negative sexual cognitions suggests that sexual function scores may not fully reflect patients’ self-perception, body exposure-related stress, or avoidance behavior in intimate contexts (1–3, 62). Research on chronic skin diseases also shows that stress, rumination, perceived stigma, body image concerns, coping style, and social support may influence patients’ psychosocial responses to disease experience (61, 63). Therefore, sexual function should be understood as one measurement node within psychosexual burden, not as a complete proxy measure. The absence of obvious abnormalities in sexual function measures does not rule out psychosexual burden related to local symptoms, body exposure, self-presentation, or partner interaction.

The functional status measured by sexual function instruments is not equivalent to the sexual distress that patients actually experience. The DLQI “not relevant” response provides a useful methodological example: when item content does not match a patient’s actual life situation, raw scores may be affected by item applicability, inapplicable responses, and scoring rules (68–71). Similarly, when sexual function instruments assume recent sexual activity, a partner relationship, attempted intercourse, or specific forms of sexual activity, but the patient’s actual situation does not match these assumptions, scores may be affected by absence of recent sexual activity, absence of a partner, no attempted intercourse, missing responses, or zero-score handling. If items imply assumptions about sex, sexual orientation, or penetrative sexual activity, interpretation across populations may also be limited (45, 46, 72–74). Studies on the IIEF suggest that when “no sexual activity,” “did not attempt intercourse,” or related inapplicable responses are scored as 0, erectile function classification may be affected, and some respondents without recent sexual activity or attempted intercourse may be shifted into more severe functional impairment categories (44, 75). Relationship status, intercourse frequency, and age may also affect interpretation of IIEF results (44, 76). The applicability of sexual function or sexual health-related instruments also requires cautious interpretation among sexual minority women and among populations without a partner or without recent sexual activity (45, 46, 72–74). Therefore, sexual function instruments should be interpreted in relation to recent sexual activity, partner status, item applicability, and forms of sexual activity. For adults with genital psoriasis, absence of sexual activity may itself be related to pain, shame, concerns about symptom worsening, relationship status, or sexual avoidance.

Therefore, sexual function instruments can serve as important measurement nodes within psychosexual burden, but they should not be used as proxy measures. Their limitations include insufficient coverage of sexual distress, sexual avoidance, and self-presentation burden, as well as the influence of recent sexual activity, partner status, scoring rules, and item applicability on score interpretation.

### 5.3 From construct misalignment to the clinical recognition gap

Construct misalignment not only indicates the measurement boundaries of existing instruments, but also affects whether related burden enters patient–clinician communication and clinical documentation. Research settings typically use prespecified questions and scale items to record local symptoms, sexual activity-related impact, quality of life, self-image, or patient needs in a structured manner. Routine dermatology practice does not necessarily provide the same entry points. Genital involvement is associated with reduced quality of life, sexual health-related burden, sexual activity-related impact, impaired sexual health-related quality of life, sexual activity avoidance, and impaired genital self-image (7, 8, 19, 20, 59), but these concerns may still lack sufficient clinical visibility in routine care (5, 10). Therefore, the absence of patient-initiated discussion of sexual health-related concerns in routine dermatology practice does not indicate that the related burden is absent.

This clinical recognition gap is also shaped by communication barriers. Adult genital psoriasis involves sensitive issues such as privacy, shame, stigma, body exposure, partner evaluation, and sexual activity. Patients may lack appropriate language to describe these concerns, or they may not view dermatology consultations as an appropriate setting for discussing sexual health-related issues. Research on adult genital psoriasis in Asian populations suggests that related burden may require more active inquiry and assessment by clinicians (13). Evidence from anogenital psoriasis also indicates that sex-related impairment, patient needs, and treatment benefits can be difficult-to-communicate topics. This suggests that recognition of related burden depends on whether patients have opportunities to express concerns, are asked about them appropriately, and are linked to appropriate follow-up care when needed (28).

Therefore, the clinical recognition gap for psychosexual burden associated with adult genital psoriasis does not stem solely from the absence of a single ideal instrument. Measurability in research does not automatically translate into clinical visibility in routine dermatology practice. The associated burden may remain outside routine dermatology practice because of a lack of inquiry entry points, communication barriers, or insufficient documentation.

## 6 A brief clinical entry point: candidate domains and future design directions

### 6.1 Positioning and design rationale

Based on the clinical recognition gap described above, the goal in routine dermatology practice is not to directly administer full sexual function scales, psychological assessments, or sexual medicine evaluations. Rather, it is to provide a brief, non-diagnostic, low-burden clinical entry point. This entry point allows genital symptoms, sexual and intimacy-related impact, self-presentation burden, and communication needs that patients may not raise spontaneously to be initially asked about, documented, and linked to further assessment or discussion when needed.

**Figure 2:**
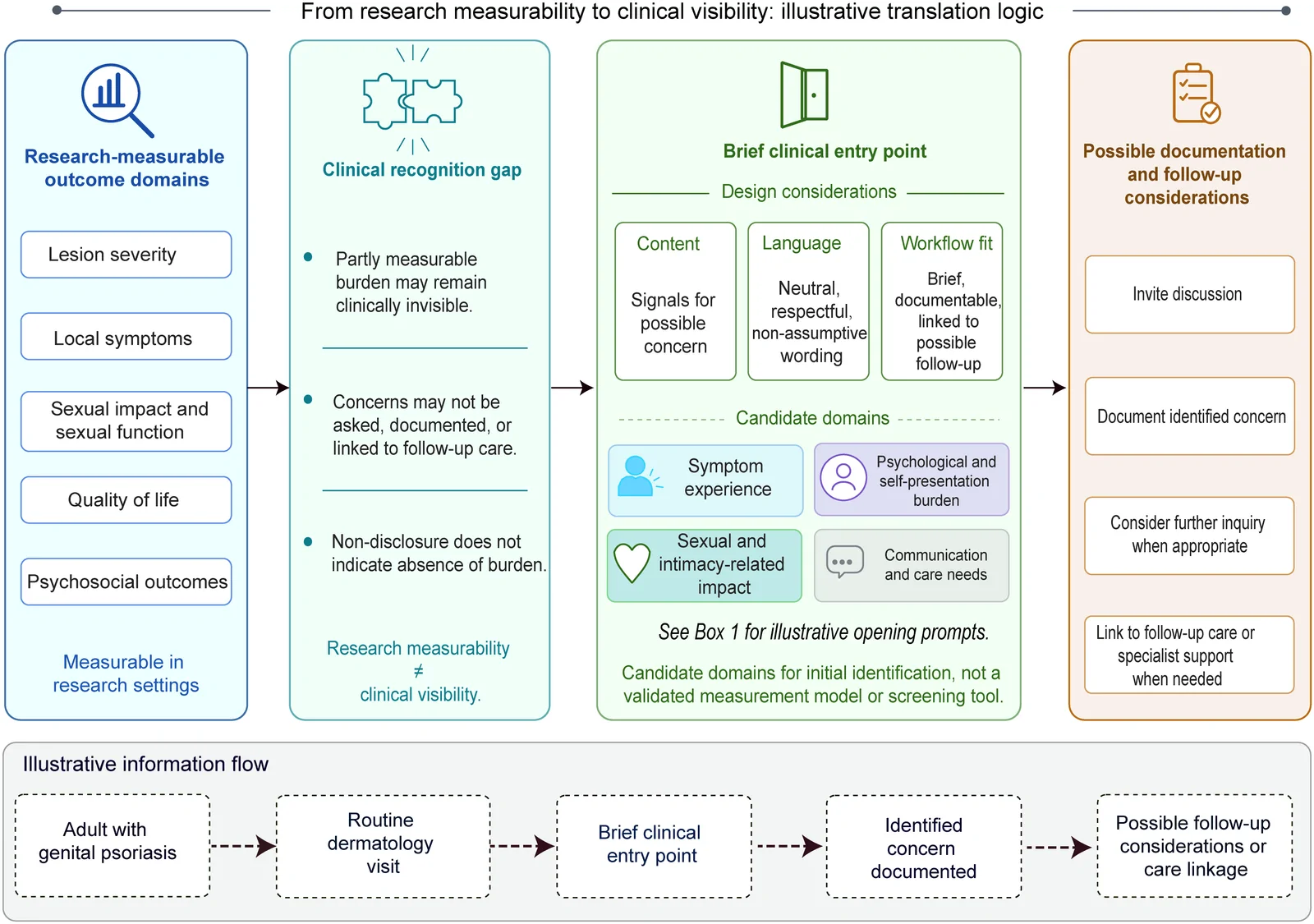
Illustrative translation logic from research measurability to clinical visibility in adult genital psoriasis. This figure distinguishes research measurability from clinical visibility in routine dermatology practice. Research-measurable outcome domains may include lesion severity, local symptoms, sexual impact, sexual function, quality of life, and psychosocial outcomes, but related concerns may still remain outside routine clinical communication and documentation. The brief clinical entry point is intended as a low-burden, non-diagnostic conversation structure for initial identification. Its design centers on content, language, and workflow fit, and uses four candidate domains: symptom experience, sexual and intimacy-related impact, psychological and self-presentation burden, communication and care needs. Illustrative opening prompts are provided in **Box 1**. Identified concerns may be documented and linked to possible follow-up considerations, including further inquiry, treatment reassessment, patient education, follow-up care, or specialist support when needed. This figure is illustrative only and does not represent a validated screening or diagnostic instrument, a validated measurement model, a standardized clinical workflow, a formal care pathway, or a clinical guideline.

Under this positioning, the role of the brief clinical entry point is to translate the four candidate domains into an organizing logic for initial identification in routine dermatology practice. Its design should consider not only which content areas it should cover, but also how it should address barriers to discussing sensitive topics. Research on communication in genital psoriasis suggests that patients’ willingness to discuss these topics may be influenced by question wording and language acceptability (29). Therefore, this entry point should initiate discussion in a neutral, respectful, and low-shame manner. The illustrative translation logic from research measurability to clinical visibility is shown in **Figure 2**.

### 6.2 Candidate domains

Building on the preceding construct analysis, the brief clinical entry point can be organized around four candidate domains: symptom experience, sexual and intimacy-related impact, psychological and self-presentation burden, communication and care needs. These domains support initial inquiry and documentation in routine dermatology practice. Within the symptom experience domain, the identification focus is whether local symptoms such as itching, pain, or friction-related discomfort become more distressing during touch, body exposure, friction, or intimate contact. This domain helps clinicians connect local symptoms with the patient’s actual sexual and intimate contexts. In the sexual and intimacy-related impact domain, the identification focus is on sexual activity, intimate relationships, physical contact, sexual avoidance, and concerns in intimate contexts. Inquiry in this domain should consider the patient’s partner status, recent sexual activity, sexual orientation, and specific forms of sexual activity. The purpose is to identify, in an open and non-presumptive manner, whether genital involvement has affected intimate behavior, sexual participation, or a sense of safety within intimate relationships. In the psychological and self-presentation burden domain, the identification focus is on shame, embarrassment, body exposure-related stress, concerns about partner evaluation, genital self-image, and self-presentation burden. This domain indicates that psychosexual burden in patients with genital psoriasis may more often present as impaired body image, avoidance of exposure, concerns about being evaluated, or difficulty presenting oneself naturally in intimate interactions. In the communication and care needs domain, identification focuses on whether patients wish to discuss related impacts further, receive disease−related explanations, consider whether further assessment or treatment−related discussion is needed, or obtain other support. This domain is intended to connect initial identification with subsequent assessment or care discussion, so that related burden is not merely documented without further response. **Box 1** provides candidate domains and illustrative discussion prompts to support brief, low-burden, non-diagnostic communication and documentation of psychosexual concerns in routine dermatology practice.

Box 1 **Candidate domains and example prompts for psychosexual burden in adult genital psoriasis.**

**Table:** 

Candidate domain	Initial focus	Example discussion prompt	Possible documentation or follow-up
Symptom experience	Distress from local symptoms during daily activities, friction, exposure, or intimate contact	“Do itching, pain, burning, or discomfort in these areas become worse or more distressing during touch, friction, exposure, or intimate contact?”	Document the symptom context and main triggers. Reassess symptom control or treatment if needed.
Sexual and intimacy-related impact	Sexual activity, intimacy, physical contact, avoidance, or related concerns	“Whether or not you currently have a partner or are sexually active, have these lesions or symptoms affected intimacy, sexual activity, or led you to avoid certain intimate situations?”	Document the scope of impact and the patient’s main concerns. Discuss sexual health, quality of life, or related support needs if needed.
Psychological and self-presentation burden	Embarrassment, shame, exposure-related stress, self-image, or concerns about how others may respond	“Have these lesions or symptoms made you feel embarrassed, uncomfortable, or ashamed, or worried about how others may respond when the area is visible or during intimacy?”	Document psychological and self-presentation burden. Provide explanation, supportive communication, or further discussion according to the patient’s needs.
Communication and care needs	Preference for further discussion, explanation, treatment adjustment, or follow-up support	“Would you like to discuss these concerns further, or review whether treatment, follow-up, or other support may need adjustment?”	Clarify patient preferences and document main needs. Arrange follow-up, patient education, treatment adjustment, or additional support if needed.

These candidate domains and example discussion prompts are intended only to support brief initial identification and documentation of relevant concerns in routine dermatology practice. They are not intended for screening, diagnosis, scoring, or fixed triage, and should not be interpreted as validated questionnaire items, scales, diagnostic instruments, clinical guidelines, scoring systems, or fixed care pathways. In practice, wording should be adapted to the patient’s language preferences, cultural background, sex and gender identity, sexual orientation, partner status, recent sexual activity status, and forms of sexual activity.

### 6.3 Positioning and outlook for initial identification

The role of the brief clinical entry point is to provide an opportunity for related concerns to enter clinical communication and documentation. When patients raise such concerns, they should not be interpreted directly as indicating sexual dysfunction or a psychological disorder. Rather, they should be treated as a starting point for further inquiry, documentation, or subsequent assessment. Subsequent discussion may focus on local symptoms and triggers, sexual and intimacy-related impact, treatment-related issues, patient education, or further communication needs, depending on the specific context (7, 13, 28). Specific responses should be guided by patient preferences, issue severity, available resources, and local clinical practice.

Because these candidate domains and the related dialogue logic have not yet been validated as a standardized instrument or fixed process, any future standardization would need to assess content validity, acceptability, feasibility, and applicability boundaries. Content validity assessment should include patient input, clinical expert input, and cognitive debriefing to determine whether the relevant content covers the target construct, is understandable, and applies to adults with genital psoriasis across different ages, sexes, gender identities, sexual orientations, partner status, recent sexual activity, forms of sexual activity, and cultural backgrounds. Future research should also evaluate whether this low−burden entry point can be accepted by patients, used naturally by clinicians in real−world dermatology practice, and generate clinical information that can be documented and discussed.

## 7 Discussion

The measurement challenge for psychosexual burden associated with adult genital psoriasis is not merely due to a limited number of instruments. The more central issue is that existing assessment components differ in measurement targets, assessment perspectives, and applicability boundaries. Lesion severity, local symptoms, sexual function, sexual health-related quality of life, and psychosocial impact each provide partial information, but they cannot be directly combined into a continuous and complete burden metric. Future studies should clearly distinguish local disease status, sexual functioning, sexual distress, and patient communication needs when selecting outcome measures.

The fragmentation of measurement also affects clinical recognition. Content that can be recorded through scales or prespecified items in research does not necessarily enter routine dermatology practice. Concerns related to genital involvement may remain clinically invisible because of shame, privacy concerns, communication barriers, or insufficient inquiry. Therefore, the core issue in the clinical recognition gap is not the absence of a single ideal instrument. Rather, measurability in research does not automatically translate into clinical visibility in routine dermatology practice. A brief, low-burden clinical communication entry point may serve as a complementary conceptual framework, but its role should be limited to initial identification and the organization of communication.

Future research should not focus only on developing additional scales. It should also evaluate which identification content and communication approaches are acceptable to patients in real-world dermatology practice, can be used naturally by clinicians, and can generate clinical information that can be documented and discussed. Future work should also address applicability across patient groups differing in sex, gender identity, sexual orientation, partner status, recent sexual activity status, forms of sexual activity, age, and cultural background. This would help ensure that the identification framework for psychosexual burden associated with adult genital psoriasis remains low-burden while staying aligned with routine dermatology practice.

## 8 Limitations

This review uses an integrative review approach, rather than a systematic review or a psychometric review based on the Consensus-based Standards for the Selection of Health Measurement Instruments (COSMIN). Therefore, its analysis of instrument validation status, construct coverage, and applicability boundaries is intended primarily to describe and interpret the existing measurement landscape, not to provide a systematic evaluation of the psychometric quality or evidence grading of each instrument. The included studies are heterogeneous in design, target population, instrument type, assessment perspective, and outcome definition. Some instruments are adult genital psoriasis-specific, whereas others derive from psoriasis, general dermatology, sexual function, sexual quality of life, or broader psychosocial measurement contexts. Therefore, different instruments should not be regarded as psychometrically equivalent, and their results should not be directly combined into a single psychosexual burden metric. Evidence from broader contexts serves only as a supplementary construct reference and cannot replace direct evidence on adult genital psoriasis. The four candidate domains and the brief clinical entry point proposed in this review remain conceptual organizing frameworks. They do not constitute a validated measurement model, screening tool, diagnostic instrument, clinical guideline, or fixed care pathway. Their content validity, acceptability, feasibility, and boundaries of clinical use still require evaluation in future research. The available evidence also remains limited in its coverage of patients differing in sex, gender identity, sexual orientation, partner status, recent sexual activity status, and cultural background.

## 9 Conclusion

Psychosexual burden in adult genital psoriasis should not be represented solely by lesion severity or sexual function scores. Existing instruments provide measurement nodes for local symptoms, sexual function, quality of life, sexual activity-related impact, and selected psychosocial constructs, but these nodes differ in measurement targets, assessment perspectives, and contexts of use. They therefore do not readily provide a complete representation of psychosexual burden. Future research should distinguish measurability in research settings from clinical visibility in routine dermatology practice. Low-burden approaches to initial identification that are acceptable to patients may allow relevant concerns to be identified, documented, and discussed in routine dermatology practice.

## Supplementary material

Supplementary Figure 1 Evidence identification and selection flow for the integrative review. This figure presents an evidence-selection flow account for this integrative review and is not intended as a PRISMA systematic review flow diagram. AGP, adult genital psoriasis; PRO, patient-reported outcome; QoL, quality of life.
