# Supplementary material for: Psychosexual burden in adult genital psoriasis: outcome measurement, construct coverage, and clinical recognition gaps
Source: Front Psychiatry. 2026 Aug 12;17:1890558. doi: 10.3389/fpsyt.2026.1890558 (PMC13507888; doi:10.3389/fpsyt.2026.1890558)
Supplement: Supplementary file 1 [file Supplementaryfile1.docx]

**Supplementary Material 1. Scoring rules for construct coverage ratings in Table 2**

# Methodological basis and rating criteria

Table 2 used structured construct coverage ratings to describe how each instrument or measurement pathway mapped to one background clinical anchor and four candidate domains. The background clinical anchor referred to severity or local disease status. It was used only as an interpretive reference and was not considered a domain of psychosexual burden in adult genital psoriasis. The four candidate analytical domains were symptom experience, sexual and intimacy-related impact, psychological and self-presentation burden, and communication and care needs.

The scoring rules were developed by drawing on methodological principles related to PROM content validity, context of use, evaluation of existing PRO instruments, directed content analysis, and coder or rater agreement. The main methodological sources included the COSMIN content validity methodology and manual (1,2), FDA PRO guidance (3), and ISPOR recommendations on the use or modification of existing PRO instruments and on the development and reporting of PRO content validity evidence (4,5). Additional sources included methodological work on directed content analysis (6) and literature on coding agreement and rater agreement (7,8). Ratings considered each instrument’s measurement target, item content, domain or subscale structure, scoring components, intended use, and published descriptions of development, validation, or application. The ratings were not intended to evaluate psychometric quality, validation status, clinical priority, or relative instrument quality.

# Scoring process

For the scoring process, two authors (Haiying Lv and Huimei Wu) independently rated each “measurement instrument or measurement pathway × Table 2 column” unit according to the rules described above. Each initial rating was restricted to ●, △, or —, and each rater recorded a brief justification based on the instrument’s measurement target, item content, scoring components, intended use, or published description. After independent rating, the two authors compared the initial ratings. Ratings that agreed were retained, and discrepant ratings were resolved through discussion. A third author (Haiming Chen) served as a prespecified adjudicator and would have been involved only if disagreement remained after discussion.

**Table A. Operational definitions of construct coverage symbols in Table 2**

| **Symbol** | **Meaning** | **Operational rule** |
| --- | --- | --- |
| ● | Direct coverage | The instrument or measurement pathway is specifically designed to measure the corresponding construct, or includes that construct as core measurement content. This applies when the construct is a primary measurement target, a core score, a named domain or subscale, or is directly represented by multiple core items. For a single-item instrument, direct coverage was assigned only when the item itself represented the prespecified measurement target. |
| △ | Partial or indirect coverage | The instrument or measurement pathway includes relevant items or provides signals related to the construct, but is not a dedicated measure of that construct. This applies when the construct is represented by one or a small number of items, appears as a secondary component, or can only be inferred indirectly from the instrument content. |
| — | No meaningful coverage | The instrument or measurement pathway does not provide interpretable measurement information related to the corresponding construct. This applies when the instrument content lacks the construct, when the construct is not part of the measurement target or scoring structure, or when the construct can only be inferred from distal clinical interpretation rather than item content. |

**Table B. Construct coverage criteria for each Table 2 column**

| **Table 2 column** | **Rating rule** |
| --- | --- |
| Background clinical anchor: severity/local disease status | A rating of ● was assigned when the instrument specifically measured genital-site or local disease severity. A rating of △ was assigned when the instrument measured overall psoriasis severity or general disease status and could serve as a background anchor, but was not genital-site specific. A rating of — was assigned when the instrument did not measure disease severity or local disease status. |
| Symptom experience | A rating of ● was assigned when the instrument primarily measured genital or local symptoms, such as itching, pain, burning, discomfort, erythema, or scaling. A rating of △ was assigned when symptoms appeared only as secondary items or indirect signals. A rating of — was assigned when no symptom content was present. |
| Sexual and intimacy-related impact | A rating of ● was assigned when the instrument primarily measured sexual activity, sexual frequency, sexual function, impaired sexual activity, sexual avoidance, sexual satisfaction, restricted intimate behavior, or sexual impact within partner relationships. A rating of △ was assigned when the instrument contained only limited sexual or intimacy-related signals, or when it could only provide indirect indications of sexual and intimacy-related impact. A rating of — was assigned when no relevant content was present. Sexual distress or sexual quality of life was not automatically assigned to this domain. It was used as evidence for this domain when it mainly reflected impairment in sexual activity, sexual function, or intimate behavior. When it mainly reflected shame, concern, body image, self-presentation pressure, or emotional burden, it was preferentially assigned to psychological and self-presentation burden. |
| Psychological and self-presentation burden | A rating of ● was assigned when the instrument primarily measured genital self-image, body image, shame, stigma, embarrassment, attractiveness concerns, partner evaluation concerns, concerns about being seen or exposed, psychological distress related to sexual or intimate contexts, or self-presentation burden. A rating of △ was assigned when these contents appeared only indirectly or as secondary content. A rating of — was assigned when no psychological or self-presentation-related content was present. Sexual distress was used as evidence for this domain only when it mainly reflected emotional distress, shame, body image concerns, self-presentation pressure, or psychological burden within relationships. When it mainly reflected sexual function or restricted sexual activity, it was preferentially assigned to sexual and intimacy-related impact. |
| Communication and care needs | A rating of ● was assigned when the instrument or pathway primarily measured patient needs, treatment goals, expected treatment benefits, care preferences, unmet care needs, communication barriers, topics that are difficult to discuss, help-seeking willingness, or needs for follow-up care linkage. A rating of △ was assigned when the instrument only indirectly suggested a need for further discussion, explanation, referral, or care linkage. A rating of — was assigned when no communication or care-needs content was present. Coverage of communication and care needs was not assigned solely because a symptom or psychosexual burden might theoretically require clinician–patient communication. |

Note: Table 2 ratings were based only on the actual content measured by each instrument. Use in genital psoriasis or at a specific anatomical site was not treated as evidence of coverage across all psychosexual burden domains. General sexual function instruments indicate sexual function or sexual response-related content, but do not represent psychosexual burden in adult genital psoriasis as a whole. For comparator instruments or instruments used as construct references, a rating was assigned only when item content could be clearly mapped to a specific domain. Validation status, reliability, responsiveness, and cross-population applicability are summarized separately in Supplementary Table S3 and were not used as rating criteria for Table 2. The symbols in Table 2 indicate conceptual construct coverage only. They do not represent instrument quality, psychometric quality, screening or diagnostic use, or a clinical care pathway.

# ****Dual rating and agreement****

Two authors (Haiying Lv and Huimei Wu) independently completed the initial ratings for 80 rating units in Table 2 according to the rules described above. Each rating unit was restricted to ●, △, or —, and a brief justification was recorded. Of the initial ratings, 78 units were concordant, corresponding to an initial percentage agreement of 97.5%. The two discrepant units were resolved through discussion, and third-author adjudication was not required. The final Table 2 presents the consensus ratings.

**References**

1.	Terwee CB, Prinsen C a. C, Chiarotto A, Westerman MJ, Patrick DL, Alonso J, Bouter LM, de Vet HCW, Mokkink LB. COSMIN methodology for evaluating the content validity of patient-reported outcome measures: a Delphi study. *Qual Life Res* (2018) 27:1159–1170. doi: 10.1007/s11136-018-1829-0

2.	COSMIN initiative. COSMIN methodology for content validity: User manual. COSMIN. (2019). https://www.cosmin.nl/wp-content/uploads/COSMIN-methodology-for-content-validity-user-manual-v1.pdf

3.	U.S. Food and Drug Administration. Patient-Reported Outcome Measures: Use in Medical Product Development to Support Labeling Claims. [Guidance for Industry]. U.S. Food and Drug Administration. (2009). https://www.fda.gov/media/77832/download

4.	Rothman M, Burke L, Erickson P, Leidy NK, Patrick DL, Petrie CD. Use of existing patient-reported outcome (PRO) instruments and their modification: the ISPOR Good Research Practices for Evaluating and Documenting Content Validity for the Use of Existing Instruments and Their Modification PRO Task Force Report. *Value Health* (2009) 12:1075–1083. doi: 10.1111/j.1524-4733.2009.00603.x

5.	Patrick DL, Burke LB, Gwaltney CJ, Leidy NK, Martin ML, Molsen E, Ring L. Content validity--establishing and reporting the evidence in newly developed patient-reported outcomes (PRO) instruments for medical product evaluation: ISPOR PRO good research practices task force report: part 1--eliciting concepts for a new PRO instrument. *Value Health* (2011) 14:967–977. doi: 10.1016/j.jval.2011.06.014

6.	Hsieh H-F, Shannon SE. Three approaches to qualitative content analysis. *Qual Health Res* (2005) 15:1277–1288. doi: 10.1177/1049732305276687

7.	McHugh ML. Interrater reliability: the kappa statistic. *Biochem Med (Zagreb)* (2012) 22:276–282.

8.	Burla L, Knierim B, Barth J, Liewald K, Duetz M, Abel T. From text to codings: intercoder reliability assessment in qualitative content analysis. *Nurs Res* (2008) 57:113–117. doi: 10.1097/01.NNR.0000313482.33917.7d
