# Supplementary material for: Psychosexual burden in adult genital psoriasis: outcome measurement, construct coverage, and clinical recognition gaps
Source: Front Psychiatry. 2026 Aug 12;17:1890558. doi: 10.3389/fpsyt.2026.1890558 (PMC13507888; doi:10.3389/fpsyt.2026.1890558)
Supplement: Supplementary file 2 [file Table1.docx]

**Supplementary Table 1. Four-database search strategies and search records**

Note: This table records the search strategies and search records for Medline, Embase, APA PsycINFO, and CINAHL. All four databases were searched on June 12, 2026, from database inception to June 12, 2026. The Medline strategy was used as the base strategy for cross-database adaptation; the other database strategies were adapted according to each platform’s syntax, subject headings, and field rules. No adult or language filters were applied at the search stage. Adult applicability and English full-text availability were assessed during subsequent screening. The main search set was used for export, deduplication, and primary screening. Targeted supplementary searches were run and recorded separately and used to support specific evidence roles, including tool applicability, score interpretation, SOGI/LGBTQ+-related representativeness limits, communication, and methodological rationale.

| Search source | Search module | Purpose | Search strategy and records |
| --- | --- | --- | --- |
| Medline | P1A: Core disease–site search. | To identify core literature on genital, anogenital, perianal, or genital-relevant special-site psoriasis. | ("Psoriasis"[Mesh] OR psoriasis[tiab] OR "psoriasis vulgaris"[tiab] OR "plaque psoriasis"[tiab]) AND (genital*[tiab] OR genitalia[tiab] OR "genital involvement"[tiab] OR "genital lesion"[tiab] OR "genital lesions"[tiab] OR "genital psoriasis"[tiab] OR anogenital[tiab] OR "anogenital psoriasis"[tiab] OR perigenital[tiab] OR vulvar[tiab] OR vulval[tiab] OR vulva[tiab] OR labia[tiab] OR labial[tiab] OR penile[tiab] OR penis[tiab] OR penoscrotal[tiab] OR glans[tiab] OR foreskin[tiab] OR prepuce[tiab] OR preputial[tiab] OR scrotal[tiab] OR scrotum[tiab] OR perianal[tiab] OR "perianal psoriasis"[tiab] OR perineal[tiab] OR perineum[tiab] OR groin[tiab] OR inguinal[tiab] OR "inguinal crease"[tiab] OR "inguinal creases"[tiab] OR intergluteal[tiab] OR "intergluteal fold"[tiab] OR "intergluteal cleft"[tiab] OR "gluteal cleft"[tiab] OR "natal cleft"[tiab] OR "inverse psoriasis"[tiab] OR "intertriginous psoriasis"[tiab] OR "flexural psoriasis"[tiab]) |
| Medline | P1B: Disease–site, outcome, measurement, communication, and clinical-recognition search. | To identify genital-relevant psoriasis literature on symptoms, sexual health, sexual impact, QoL, PROs, measurement, patient needs, communication, documentation, referral, follow-up care, or clinical recognition. | ("Psoriasis"[Mesh] OR psoriasis[tiab] OR "psoriasis vulgaris"[tiab] OR "plaque psoriasis"[tiab]) AND (genital*[tiab] OR genitalia[tiab] OR "genital involvement"[tiab] OR "genital lesion"[tiab] OR "genital lesions"[tiab] OR "genital psoriasis"[tiab] OR anogenital[tiab] OR "anogenital psoriasis"[tiab] OR perigenital[tiab] OR vulvar[tiab] OR vulval[tiab] OR vulva[tiab] OR labia[tiab] OR labial[tiab] OR penile[tiab] OR penis[tiab] OR penoscrotal[tiab] OR glans[tiab] OR foreskin[tiab] OR prepuce[tiab] OR preputial[tiab] OR scrotal[tiab] OR scrotum[tiab] OR perianal[tiab] OR "perianal psoriasis"[tiab] OR perineal[tiab] OR perineum[tiab] OR groin[tiab] OR inguinal[tiab] OR "inguinal crease"[tiab] OR "inguinal creases"[tiab] OR intergluteal[tiab] OR "intergluteal fold"[tiab] OR "intergluteal cleft"[tiab] OR "gluteal cleft"[tiab] OR "natal cleft"[tiab] OR "inverse psoriasis"[tiab] OR "intertriginous psoriasis"[tiab] OR "flexural psoriasis"[tiab]) AND (symptom*[tiab] OR itch*[tiab] OR prurit*[tiab] OR pain[tiab] OR painful[tiab] OR burning[tiab] OR fissur*[tiab] OR discomfort[tiab] OR friction[tiab] OR "sexual health"[tiab] OR psychosexual[tiab] OR "sexual function"[tiab] OR "sexual dysfunction"[tiab] OR "sexual distress"[tiab] OR "sexual activity"[tiab] OR "sexual frequency"[tiab] OR "sexual impact"[tiab] OR "sexual impairment"[tiab] OR "sexual avoidance"[tiab] OR intimacy[tiab] OR "intimate relationship"[tiab] OR "intimate relationships"[tiab] OR "quality of life"[Mesh] OR "quality of life"[tiab] OR QoL[tiab] OR "sexual quality of life"[tiab] OR "patient-reported outcome"[tiab] OR "patient-reported outcomes"[tiab] OR "patient reported outcome"[tiab] OR "patient reported outcomes"[tiab] OR questionnaire*[tiab] OR scale*[tiab] OR instrument*[tiab] OR measure*[tiab] OR measurement[tiab] OR assessment*[tiab] OR endpoint*[tiab] OR psychometric*[tiab] OR valid*[tiab] OR validation[tiab] OR reliability[tiab] OR responsiveness[tiab] OR "content validity"[tiab] OR "construct validity"[tiab] OR "score interpretation"[tiab] OR "patient needs"[tiab] OR "unmet needs"[tiab] OR "treatment benefit"[tiab] OR "treatment benefits"[tiab] OR communication[tiab] OR discuss*[tiab] OR conversation*[tiab] OR "clinical recognition"[tiab] OR "clinical visibility"[tiab] OR documentation[tiab] OR referral[tiab] OR "follow-up care"[tiab]) |
| Medline | P2A: GenPs-specific or genital-site-specific instrument search. | To identify genital psoriasis-specific or genital-site-specific instrument names, endpoint names, study-specific measurement paths, trial outcome terms, and source reports. | ("Psoriasis"[Mesh] OR psoriasis[tiab] OR "genital psoriasis"[tiab] OR "anogenital psoriasis"[tiab] OR "perianal psoriasis"[tiab]) AND ("sPGA-G"[tiab] OR "static Physician's Global Assessment of Genitalia"[tiab] OR "static Physician Global Assessment of Genitalia"[tiab] OR "genital PGA"[tiab] OR "genital Physician's Global Assessment"[tiab] OR "genital Physician Global Assessment"[tiab] OR "physician global assessment of genital psoriasis"[tiab] OR "Patient Global Assessment-Genital"[tiab] OR "Patient's Global Assessment of Genital Psoriasis"[tiab] OR "Patient Global Assessment of Genital Psoriasis"[tiab] OR "PatGA-Genital"[tiab] OR mGPASI[tiab] OR "modified Genital Psoriasis Area and Severity Index"[tiab] OR "modified genital PASI"[tiab] OR GPSS[tiab] OR "Genital Psoriasis Symptoms Scale"[tiab] OR "GPI-NRS"[tiab] OR "Genital Psoriasis Itch Numeric Rating Scale"[tiab] OR "genital itch Numeric Rating Scale"[tiab] OR "genital itch NRS"[tiab] OR "GenPs-Itch NRS"[tiab] OR "GenPs-SFQ"[tiab] OR "Genital Psoriasis Sexual Frequency Questionnaire"[tiab] OR "Sexual Frequency Questionnaire"[tiab] OR GPSIS[tiab] OR "Genital Psoriasis Sexual Impact Scale"[tiab] OR "Reasons for Sexual Impairment"[tiab] OR "reasons for sexual impairment"[tiab] OR (RSI[tiab] AND psoriasis[tiab] AND ("sexual impairment"[tiab] OR "sexual impact"[tiab] OR "sexual activity"[tiab] OR "sexual avoidance"[tiab]))) |
| Medline | P2B: GenPs-specific or genital-site-specific instruments plus validation and psychometric terms. | To identify evidence on the development, validation, content validity, reliability, responsiveness, score interpretation, and psychometric status of genital psoriasis-specific or genital-site-specific instruments. | ("Psoriasis"[Mesh] OR psoriasis[tiab] OR "genital psoriasis"[tiab] OR "anogenital psoriasis"[tiab] OR "perianal psoriasis"[tiab]) AND ("sPGA-G"[tiab] OR "static Physician's Global Assessment of Genitalia"[tiab] OR "static Physician Global Assessment of Genitalia"[tiab] OR "genital PGA"[tiab] OR "genital Physician's Global Assessment"[tiab] OR "genital Physician Global Assessment"[tiab] OR "physician global assessment of genital psoriasis"[tiab] OR "Patient Global Assessment-Genital"[tiab] OR "Patient's Global Assessment of Genital Psoriasis"[tiab] OR "Patient Global Assessment of Genital Psoriasis"[tiab] OR "PatGA-Genital"[tiab] OR mGPASI[tiab] OR "modified Genital Psoriasis Area and Severity Index"[tiab] OR "modified genital PASI"[tiab] OR GPSS[tiab] OR "Genital Psoriasis Symptoms Scale"[tiab] OR "GPI-NRS"[tiab] OR "Genital Psoriasis Itch Numeric Rating Scale"[tiab] OR "genital itch Numeric Rating Scale"[tiab] OR "genital itch NRS"[tiab] OR "GenPs-Itch NRS"[tiab] OR "GenPs-SFQ"[tiab] OR "Genital Psoriasis Sexual Frequency Questionnaire"[tiab] OR "Sexual Frequency Questionnaire"[tiab] OR GPSIS[tiab] OR "Genital Psoriasis Sexual Impact Scale"[tiab] OR "Reasons for Sexual Impairment"[tiab] OR "reasons for sexual impairment"[tiab] OR (RSI[tiab] AND psoriasis[tiab] AND ("sexual impairment"[tiab] OR "sexual impact"[tiab] OR "sexual activity"[tiab] OR "sexual avoidance"[tiab]))) AND (psychometric*[tiab] OR validation[tiab] OR validated[tiab] OR validity[tiab] OR valid*[tiab] OR reliability[tiab] OR reliable[tiab] OR responsiveness[tiab] OR responsive[tiab] OR "content validity"[tiab] OR "construct validity"[tiab] OR "structural validity"[tiab] OR "factor analysis"[tiab] OR "internal consistency"[tiab] OR "test-retest"[tiab] OR "cognitive debriefing"[tiab] OR "item generation"[tiab] OR "item reduction"[tiab] OR "score interpretation"[tiab] OR interpretability[tiab] OR "meaningful change"[tiab] OR "minimally important difference"[tiab] OR "patient-reported outcome"[tiab] OR "patient-reported outcomes"[tiab] OR "clinical outcome assessment"[tiab] OR "clinical outcome assessments"[tiab]) |
| Medline | P2C: Focused search for dermatology-, psoriasis-, skin-related sexuality, and generic comparator instruments. | To identify dermatology-related, psoriasis-related, skin-related sexuality, generic sexual function, sexual distress, genital self-image, intimacy, stigma, patient needs, and comparator instruments. | ("Psoriasis"[Mesh] OR psoriasis[tiab] OR dermatolog*[tiab] OR "skin disease"[tiab] OR "skin diseases"[tiab] OR "chronic skin disease"[tiab] OR "chronic skin diseases"[tiab] OR "visible skin disease"[tiab] OR "visible skin diseases"[tiab] OR "cutaneous disease"[tiab] OR "cutaneous diseases"[tiab] OR "vulvar dermatosis"[tiab] OR "vulvar dermatoses"[tiab] OR "genital dermatosis"[tiab] OR "genital dermatoses"[tiab]) AND ("Female Sexual Function Index"[tiab] OR FSFI[tiab] OR "International Index of Erectile Function"[tiab] OR IIEF[tiab] OR "IIEF-5"[tiab] OR "Arizona Sexual Experience Scale"[tiab] OR "Arizona Sexual Experiences Scale"[tiab] OR ASEX[tiab] OR "Massachusetts General Hospital Sexual Functioning Questionnaire"[tiab] OR "MGH-SFQ"[tiab] OR "Female Sexual Distress Scale"[tiab] OR "Female Sexual Distress Scale-Revised"[tiab] OR FSDS[tiab] OR "FSDS-R"[tiab] OR QualipsoSex[tiab] OR Qualipsosex[tiab] OR "QualipsoSex questionnaire"[tiab] OR "Qualipsosex questionnaire"[tiab] OR "Skin-Related Sexual Life Questionnaire"[tiab] OR "Skin Related Sexual Life Questionnaire"[tiab] OR SRSLQ[tiab] OR "Sexual Quality of Life Questionnaire for Men"[tiab] OR "Sexual Quality of Life Questionnaire for use in Men"[tiab] OR "SQoL-M"[tiab] OR "Female Genital Self-Image Scale"[tiab] OR FGSIS[tiab] OR "Dermatology Life Quality Index"[tiab] OR DLQI[tiab] OR "DLQI item 9"[tiab] OR "sexual difficulties item"[tiab] OR "Skindex-16"[tiab] OR "Vulval Quality of Life Index"[tiab] OR "Vulvar Quality of Life Index"[tiab] OR VQLI[tiab] OR VLQI[tiab] OR "Psoriasis Disability Index"[tiab] OR PDI[tiab] OR "Psoriasis Internalized Stigma Scale"[tiab] OR PISS[tiab] OR "Relationship and Sexuality Scale"[tiab] OR "Revised Dyadic Adjustment Scale"[tiab] OR "Dermatologic Intimacy Scale"[tiab] OR "Perceived Stigmatization Questionnaire"[tiab] OR "Dysmorphic Concern Questionnaire"[tiab] OR "Patient Benefit Index"[tiab] OR PBI[tiab] OR "Patient Needs Questionnaire"[tiab] OR "Patient Benefit Questionnaire"[tiab] OR "Comprehensive Assessment of Psoriasis-related Patient-reported outcomes"[tiab]) AND ("sexual health"[tiab] OR psychosexual[tiab] OR "sexual function"[tiab] OR "sexual dysfunction"[tiab] OR "sexual distress"[tiab] OR "sexual quality of life"[tiab] OR "skin-related sexuality"[tiab] OR sexuality[tiab] OR intimacy[tiab] OR "body image"[tiab] OR "genital self-image"[tiab] OR "sexual self-image"[tiab] OR stigma*[tiab] OR "self-stigma"[tiab] OR "internalized stigma"[tiab] OR shame[tiab] OR embarrassment[tiab] OR "quality of life"[tiab] OR QoL[tiab] OR psychometric*[tiab] OR valid*[tiab] OR validation[tiab] OR reliability[tiab] OR responsiveness[tiab] OR "content validity"[tiab] OR "construct validity"[tiab] OR "structural validity"[tiab] OR "factor analysis"[tiab] OR "score interpretation"[tiab] OR interpretability[tiab] OR "meaningful change"[tiab] OR "minimally important difference"[tiab] OR "not relevant"[tiab] OR "not applicable"[tiab] OR "not sexually active"[tiab] OR "no sexual activity"[tiab] OR "no partner"[tiab] OR "partner status"[tiab] OR "current sexual activity"[tiab] OR "sexual orientation"[tiab] OR "gender identity"[tiab] OR transgender[tiab] OR "gender diverse"[tiab] OR "same-sex partnership"[tiab]) |
| Medline | P3A-2: Targeted construct-evidence search with measurement and interpretation terms. | To identify supplementary construct evidence that includes construct terms together with measurement, instruments, PROs, psychometrics, communication, clinical recognition, or patient needs. | ("Psoriasis"[Mesh] OR psoriasis[tiab] OR dermatolog*[tiab] OR "skin disease"[tiab] OR "skin diseases"[tiab] OR "chronic skin disease"[tiab] OR "chronic skin diseases"[tiab] OR "visible skin disease"[tiab] OR "visible skin diseases"[tiab] OR "cutaneous disease"[tiab] OR "cutaneous diseases"[tiab] OR "vulvar dermatosis"[tiab] OR "vulvar dermatoses"[tiab] OR "genital dermatosis"[tiab] OR "genital dermatoses"[tiab]) AND (psychosexual[tiab] OR "sexual distress"[tiab] OR "sexual avoidance"[tiab] OR "sexual quality of life"[tiab] OR "sexual health"[tiab] OR "sexual function"[tiab] OR "sexual dysfunction"[tiab] OR "sexual impairment"[tiab] OR "sexual activity"[tiab] OR "sexual self-image"[tiab] OR "genital self-image"[tiab] OR "body image"[tiab] OR "appearance concern"[tiab] OR "appearance concerns"[tiab] OR shame[tiab] OR embarrassment[tiab] OR stigma[tiab] OR stigmatization[tiab] OR stigmatisation[tiab] OR "self-stigma"[tiab] OR "internalized stigma"[tiab] OR "internalised stigma"[tiab] OR intimacy[tiab] OR "intimate relationship"[tiab] OR "intimate relationships"[tiab] OR "relationship burden"[tiab] OR "partner evaluation"[tiab] OR "fear of rejection"[tiab] OR avoidance[tiab]) AND (measurement[tiab] OR measure*[tiab] OR instrument*[tiab] OR questionnaire*[tiab] OR scale*[tiab] OR construct[tiab] OR constructs[tiab] OR "patient-reported outcome"[tiab] OR "patient-reported outcomes"[tiab] OR "patient reported outcome"[tiab] OR "patient reported outcomes"[tiab] OR psychometric*[tiab] OR valid*[tiab] OR validation[tiab] OR "score interpretation"[tiab] OR communication[tiab] OR "clinical recognition"[tiab] OR "clinical visibility"[tiab] OR "patient needs"[tiab] OR "unmet needs"[tiab]) |
| Medline | P3B-1-revised: Patient-needs and care-context search. | To identify evidence on patient needs, unmet needs, patient preferences, patient–clinician communication, clinical recognition, and clinical visibility in psoriasis, dermatology, or genital-dermatology literature. | ("Psoriasis"[Mesh] OR psoriasis[tiab] OR dermatolog*[tiab] OR "skin disease"[tiab] OR "skin diseases"[tiab] OR "chronic skin disease"[tiab] OR "chronic skin diseases"[tiab] OR "visible skin disease"[tiab] OR "visible skin diseases"[tiab] OR "cutaneous disease"[tiab] OR "cutaneous diseases"[tiab] OR "dermatologic disease"[tiab] OR "dermatologic diseases"[tiab] OR "vulvar dermatosis"[tiab] OR "vulvar dermatoses"[tiab] OR "genital dermatosis"[tiab] OR "genital dermatoses"[tiab] OR ((genital[tiab] OR anogenital[tiab] OR perianal[tiab] OR vulvar[tiab] OR vulval[tiab]) AND (dermatolog*[tiab] OR dermatos*[tiab] OR cutaneous[tiab] OR skin[tiab]))) AND ("patient needs"[tiab] OR "unmet needs"[tiab] OR "patient need"[tiab] OR "unmet need"[tiab] OR "patient preference"[tiab] OR "patient preferences"[tiab] OR "Patient Benefit Index"[tiab] OR "Patient Needs Questionnaire"[tiab] OR "Patient Benefit Questionnaire"[tiab] OR "patient-clinician communication"[tiab] OR "clinician-patient communication"[tiab] OR "physician-patient communication"[tiab] OR "doctor-patient communication"[tiab] OR "difficult-to-communicate"[tiab] OR "preferred language"[tiab] OR "dermatology practice"[tiab] OR "dermatology visit"[tiab] OR "dermatology visits"[tiab] OR "routine dermatology"[tiab] OR "outpatient dermatology"[tiab] OR "clinical recognition"[tiab] OR "clinical visibility"[tiab]) AND (genital[tiab] OR genitalia[tiab] OR anogenital[tiab] OR perianal[tiab] OR vulvar[tiab] OR vulval[tiab] OR sexual[tiab] OR psychosexual[tiab] OR "sexual health"[tiab] OR "sexual function"[tiab] OR "sexual distress"[tiab] OR "sexual avoidance"[tiab] OR intimacy[tiab] OR "body image"[tiab] OR "genital self-image"[tiab] OR stigma*[tiab] OR shame[tiab] OR embarrassment[tiab] OR "quality of life"[tiab] OR QoL[tiab] OR "patient-reported outcome"[tiab] OR "patient-reported outcomes"[tiab] OR communication[tiab] OR "clinical recognition"[tiab] OR "clinical visibility"[tiab]) |
| Medline | P3A-1: Broad supplementary construct search. | To identify supplementary construct evidence related to sexual function versus sexual health, sexual distress, sexual avoidance, body image, genital self-image, sexual self-image, shame, stigma, intimacy, and relationship burden. | ("Psoriasis"[Mesh] OR psoriasis[tiab] OR dermatolog*[tiab] OR "skin disease"[tiab] OR "skin diseases"[tiab] OR "chronic skin disease"[tiab] OR "chronic skin diseases"[tiab] OR "visible skin disease"[tiab] OR "visible skin diseases"[tiab] OR "cutaneous disease"[tiab] OR "cutaneous diseases"[tiab] OR "vulvar dermatosis"[tiab] OR "vulvar dermatoses"[tiab] OR "genital dermatosis"[tiab] OR "genital dermatoses"[tiab]) AND (psychosexual[tiab] OR "sexual distress"[tiab] OR "sexual avoidance"[tiab] OR "sexual quality of life"[tiab] OR "sexual health"[tiab] OR "sexual function"[tiab] OR "sexual dysfunction"[tiab] OR "sexual impairment"[tiab] OR "sexual activity"[tiab] OR "sexual self-image"[tiab] OR "genital self-image"[tiab] OR "body image"[tiab] OR "appearance concern"[tiab] OR "appearance concerns"[tiab] OR shame[tiab] OR embarrassment[tiab] OR stigma[tiab] OR stigmatization[tiab] OR stigmatisation[tiab] OR "self-stigma"[tiab] OR "internalized stigma"[tiab] OR "internalised stigma"[tiab] OR intimacy[tiab] OR "intimate relationship"[tiab] OR "intimate relationships"[tiab] OR "relationship burden"[tiab] OR "partner evaluation"[tiab] OR "fear of rejection"[tiab] OR avoidance[tiab]) |
| Medline | P3B-2: Sexual-health and genital-sensitive communication, and clinical-recognition search. | To identify evidence related to sexual-health or genital-sensitive communication, clinical recognition, documentation, referral, and follow-up care. | ("Psoriasis"[Mesh] OR psoriasis[tiab] OR dermatolog*[tiab] OR "skin disease"[tiab] OR "skin diseases"[tiab] OR "chronic skin disease"[tiab] OR "chronic skin diseases"[tiab] OR "visible skin disease"[tiab] OR "visible skin diseases"[tiab]) AND ("sexual health discussion"[tiab] OR "sexual health communication"[tiab] OR "sexual communication"[tiab] OR "patient-clinician communication"[tiab] OR "clinician-patient communication"[tiab] OR "physician-patient communication"[tiab] OR "doctor-patient communication"[tiab] OR "sensitive topic"[tiab] OR "sensitive topics"[tiab] OR "sensitive issue"[tiab] OR "sensitive issues"[tiab] OR "difficult-to-communicate"[tiab] OR "preferred language"[tiab] OR disclosure[tiab] OR disclose*[tiab] OR discuss*[tiab] OR conversation*[tiab] OR "clinical recognition"[tiab] OR "clinical visibility"[tiab] OR documentation[tiab] OR "medical record"[tiab] OR "medical records"[tiab] OR referral[tiab] OR "follow-up care"[tiab] OR triage[tiab] OR "initial identification"[tiab]) AND (genital[tiab] OR genitalia[tiab] OR "genital psoriasis"[tiab] OR "genital involvement"[tiab] OR anogenital[tiab] OR perianal[tiab] OR vulvar[tiab] OR vulval[tiab] OR psychosexual[tiab] OR "sexual health"[tiab] OR "sexual function"[tiab] OR "sexual dysfunction"[tiab] OR "sexual distress"[tiab] OR "sexual avoidance"[tiab] OR "sexual activity"[tiab] OR "sexual quality of life"[tiab] OR intimacy[tiab] OR "intimate relationship"[tiab] OR "intimate relationships"[tiab] OR "genital self-image"[tiab] OR "sexual self-image"[tiab] OR (("body image"[tiab] OR stigma*[tiab] OR shame[tiab] OR embarrassment[tiab]) AND (sexual[tiab] OR genital[tiab] OR intimacy[tiab] OR psychosexual[tiab]))) |
| Medline | P3C-1: DLQI “not relevant/not applicable” and score-interpretation search. | To identify literature on DLQI item 9, not relevant/not applicable responses, inapplicable items, scoring, interpretability, content relevance, and score interpretation. | ("Dermatology Life Quality Index"[tiab] OR DLQI[tiab]) AND (psoriasis[tiab] OR dermatolog*[tiab] OR "skin disease"[tiab] OR "skin diseases"[tiab] OR "quality of life"[tiab]) AND ("not relevant"[tiab] OR "not applicable"[tiab] OR inapplicable[tiab] OR "response option"[tiab] OR scoring[tiab] OR "score interpretation"[tiab] OR interpretability[tiab] OR "content validity"[tiab] OR "content relevance"[tiab] OR "missing data"[tiab]) |
| Medline | P3C-2: Applicability and inapplicable-item search for sexual function scales. | To identify literature on inapplicable items, not sexually active status, no partner, response options, missing data, floor effects, sexual orientation, and gender-identity applicability in sexual function, sexual distress, and sexual QoL scales. | ("Female Sexual Function Index"[tiab] OR FSFI[tiab] OR "International Index of Erectile Function"[tiab] OR IIEF[tiab] OR "IIEF-5"[tiab] OR "Arizona Sexual Experience Scale"[tiab] OR ASEX[tiab] OR "Massachusetts General Hospital Sexual Functioning Questionnaire"[tiab] OR "MGH-SFQ"[tiab] OR "Female Sexual Distress Scale"[tiab] OR FSDS[tiab] OR "FSDS-R"[tiab] OR "Sexual Quality of Life Questionnaire for Men"[tiab] OR "SQoL-M"[tiab]) AND ("not applicable"[tiab] OR "not relevant"[tiab] OR inapplicable[tiab] OR "response option"[tiab] OR "not sexually active"[tiab] OR "no sexual activity"[tiab] OR "no partner"[tiab] OR "partner status"[tiab] OR "current sexual activity"[tiab] OR "missing data"[tiab] OR "floor effect"[tiab] OR heterosexual[tiab] OR "penetrative activity"[tiab] OR "sexual orientation"[tiab] OR "gender identity"[tiab]) AND (validity[tiab] OR psychometric*[tiab] OR "content validity"[tiab] OR "content relevance"[tiab] OR "score interpretation"[tiab] OR scoring[tiab] OR interpretability[tiab]) |
| Medline | P3C-3: Validity-theory and construct-misalignment search. | To identify methodological evidence on validity theory, construct underrepresentation, construct-irrelevant variance, and construct misalignment. | ("construct underrepresentation"[tiab] OR "construct-irrelevant variance"[tiab] OR "construct irrelevant variance"[tiab] OR "validity theory"[tiab] OR ("construct validity"[tiab] AND (underrepresentation[tiab] OR "irrelevant variance"[tiab]))) AND ("patient-reported outcome"[tiab] OR "patient-reported outcomes"[tiab] OR "patient reported outcome"[tiab] OR "patient reported outcomes"[tiab] OR "clinical outcome assessment"[tiab] OR "clinical outcome assessments"[tiab] OR questionnaire*[tiab] OR scale*[tiab] OR instrument*[tiab] OR measurement[tiab] OR psychometric*[tiab]) |
| Medline | SOGI/LGBTQ+ applicability and underrepresentation supplementary search. | To identify evidence on SOGI/LGBTQ+ population applicability, communication needs, underrepresentation, and measurement boundaries in psoriasis, dermatology, or genital-dermatology literature. | (psoriasis[tiab] OR dermatolog*[tiab] OR "skin disease"[tiab] OR "skin diseases"[tiab] OR "chronic skin disease"[tiab] OR "chronic skin diseases"[tiab] OR "visible skin disease"[tiab] OR "visible skin diseases"[tiab] OR "cutaneous disease"[tiab] OR "cutaneous diseases"[tiab] OR "vulvar dermatosis"[tiab] OR "vulvar dermatoses"[tiab] OR "genital dermatosis"[tiab] OR "genital dermatoses"[tiab]) AND (LGBT*[tiab] OR LGBTQ*[tiab] OR "sexual minority"[tiab] OR "sexual minorities"[tiab] OR "gender minority"[tiab] OR "gender minorities"[tiab] OR "sexual orientation"[tiab] OR "gender identity"[tiab] OR transgender[tiab] OR nonbinary[tiab] OR "non-binary"[tiab] OR "gender diverse"[tiab] OR "same-sex partner"[tiab] OR "same-sex partnership"[tiab]) AND ("sexual health"[tiab] OR "sexual distress"[tiab] OR "sexual function"[tiab] OR "body image"[tiab] OR "genital self-image"[tiab] OR stigma[tiab] OR communication[tiab] OR "patient-reported outcome"[tiab] OR "patient-reported outcomes"[tiab] OR "clinical outcome assessment"[tiab] OR measurement[tiab] OR validity[tiab] OR applicability[tiab] OR underrepresentation[tiab] OR "measurement boundary"[tiab] OR "measurement boundaries"[tiab]) |
| Medline | Search implementation summary |  | Main search: P1A OR P1B OR P2A OR P2B OR P2C OR P3A-2 OR P3B-1-revised.  Targeted supplementary searches: P3A-1, P3B-2, P3C-1, P3C-2, P3C-3, and the SOGI/LGBTQ+ applicability/underrepresentation search were run and recorded separately and were not included in the main search set. |
|  | | | |
| Embase via Embase.com | P1A: Core disease–site search. | To identify core literature on genital, anogenital, perianal, or genital-relevant special-site psoriasis. | ( 'psoriasis'/exp OR psoriasis:ti,ab,kw OR 'psoriasis vulgaris':ti,ab,kw OR 'plaque psoriasis':ti,ab,kw ) AND ( genital*:ti,ab,kw OR genitalia:ti,ab,kw OR 'genital involvement':ti,ab,kw OR 'genital lesion':ti,ab,kw OR 'genital lesions':ti,ab,kw OR 'genital psoriasis':ti,ab,kw OR anogenital:ti,ab,kw OR 'anogenital psoriasis':ti,ab,kw OR perigenital:ti,ab,kw OR vulvar:ti,ab,kw OR vulval:ti,ab,kw OR vulva:ti,ab,kw OR labia:ti,ab,kw OR labial:ti,ab,kw OR penile:ti,ab,kw OR penis:ti,ab,kw OR penoscrotal:ti,ab,kw OR glans:ti,ab,kw OR foreskin:ti,ab,kw OR prepuce:ti,ab,kw OR preputial:ti,ab,kw OR scrotal:ti,ab,kw OR scrotum:ti,ab,kw OR perianal:ti,ab,kw OR 'perianal psoriasis':ti,ab,kw OR perineal:ti,ab,kw OR perineum:ti,ab,kw OR groin:ti,ab,kw OR inguinal:ti,ab,kw OR 'inguinal crease':ti,ab,kw OR 'inguinal creases':ti,ab,kw OR intergluteal:ti,ab,kw OR 'intergluteal fold':ti,ab,kw OR 'intergluteal cleft':ti,ab,kw OR 'gluteal cleft':ti,ab,kw OR 'natal cleft':ti,ab,kw OR 'inverse psoriasis':ti,ab,kw OR 'intertriginous psoriasis':ti,ab,kw OR 'flexural psoriasis':ti,ab,kw ) |
| Embase via Embase.com | P1B: Disease–site, outcome, measurement, communication, and clinical-recognition search. | To identify genital-relevant psoriasis literature on symptoms, sexual health, sexual impact, QoL, PROs, measurement, patient needs, communication, documentation, referral, follow-up care, or clinical recognition. | ( 'psoriasis'/exp OR psoriasis:ti,ab,kw OR 'psoriasis vulgaris':ti,ab,kw OR 'plaque psoriasis':ti,ab,kw ) AND ( genital*:ti,ab,kw OR genitalia:ti,ab,kw OR 'genital involvement':ti,ab,kw OR 'genital lesion':ti,ab,kw OR 'genital lesions':ti,ab,kw OR 'genital psoriasis':ti,ab,kw OR anogenital:ti,ab,kw OR 'anogenital psoriasis':ti,ab,kw OR perigenital:ti,ab,kw OR vulvar:ti,ab,kw OR vulval:ti,ab,kw OR vulva:ti,ab,kw OR labia:ti,ab,kw OR labial:ti,ab,kw OR penile:ti,ab,kw OR penis:ti,ab,kw OR penoscrotal:ti,ab,kw OR glans:ti,ab,kw OR foreskin:ti,ab,kw OR prepuce:ti,ab,kw OR preputial:ti,ab,kw OR scrotal:ti,ab,kw OR scrotum:ti,ab,kw OR perianal:ti,ab,kw OR 'perianal psoriasis':ti,ab,kw OR perineal:ti,ab,kw OR perineum:ti,ab,kw OR groin:ti,ab,kw OR inguinal:ti,ab,kw OR 'inguinal crease':ti,ab,kw OR 'inguinal creases':ti,ab,kw OR intergluteal:ti,ab,kw OR 'intergluteal fold':ti,ab,kw OR 'intergluteal cleft':ti,ab,kw OR 'gluteal cleft':ti,ab,kw OR 'natal cleft':ti,ab,kw OR 'inverse psoriasis':ti,ab,kw OR 'intertriginous psoriasis':ti,ab,kw OR 'flexural psoriasis':ti,ab,kw ) AND ( symptom*:ti,ab,kw OR itch*:ti,ab,kw OR prurit*:ti,ab,kw OR pain:ti,ab,kw OR painful:ti,ab,kw OR burning:ti,ab,kw OR fissur*:ti,ab,kw OR discomfort:ti,ab,kw OR friction:ti,ab,kw OR 'sexual health':ti,ab,kw OR psychosexual:ti,ab,kw OR 'sexual function':ti,ab,kw OR 'sexual dysfunction':ti,ab,kw OR 'sexual distress':ti,ab,kw OR 'sexual activity':ti,ab,kw OR 'sexual frequency':ti,ab,kw OR 'sexual impact':ti,ab,kw OR 'sexual impairment':ti,ab,kw OR 'sexual avoidance':ti,ab,kw OR intimacy:ti,ab,kw OR 'intimate relationship':ti,ab,kw OR 'intimate relationships':ti,ab,kw OR 'quality of life'/exp OR 'quality of life':ti,ab,kw OR QoL:ti,ab,kw OR 'sexual quality of life':ti,ab,kw OR 'patient-reported outcome':ti,ab,kw OR 'patient-reported outcomes':ti,ab,kw OR 'patient reported outcome':ti,ab,kw OR 'patient reported outcomes':ti,ab,kw OR questionnaire*:ti,ab,kw OR scale*:ti,ab,kw OR instrument*:ti,ab,kw OR measure*:ti,ab,kw OR measurement:ti,ab,kw OR assessment*:ti,ab,kw OR endpoint*:ti,ab,kw OR psychometric*:ti,ab,kw OR valid*:ti,ab,kw OR validation:ti,ab,kw OR reliability:ti,ab,kw OR responsiveness:ti,ab,kw OR 'content validity':ti,ab,kw OR 'construct validity':ti,ab,kw OR 'score interpretation':ti,ab,kw OR 'patient needs':ti,ab,kw OR 'unmet needs':ti,ab,kw OR 'treatment benefit':ti,ab,kw OR 'treatment benefits':ti,ab,kw OR communication:ti,ab,kw OR discuss*:ti,ab,kw OR conversation*:ti,ab,kw OR 'clinical recognition':ti,ab,kw OR 'clinical visibility':ti,ab,kw OR documentation:ti,ab,kw OR referral:ti,ab,kw OR 'follow-up care':ti,ab,kw ) |
| Embase via Embase.com | P2A: GenPs-specific or genital-site-specific instrument search. | To identify genital psoriasis-specific or genital-site-specific instrument names, endpoint names, study-specific measurement paths, trial outcome terms, and source reports. | ( 'psoriasis'/exp OR psoriasis:ti,ab,kw OR 'genital psoriasis':ti,ab,kw OR 'anogenital psoriasis':ti,ab,kw OR 'perianal psoriasis':ti,ab,kw ) AND ( 'sPGA-G':ti,ab,kw OR 'static Physician Global Assessment of Genitalia':ti,ab,kw OR "static Physician's Global Assessment of Genitalia":ti,ab,kw OR 'genital PGA':ti,ab,kw OR 'genital Physician Global Assessment':ti,ab,kw OR "genital Physician's Global Assessment":ti,ab,kw OR 'physician global assessment of genital psoriasis':ti,ab,kw OR 'Patient Global Assessment-Genital':ti,ab,kw OR "Patient's Global Assessment of Genital Psoriasis":ti,ab,kw OR 'Patient Global Assessment of Genital Psoriasis':ti,ab,kw OR 'PatGA-Genital':ti,ab,kw OR mGPASI:ti,ab,kw OR 'modified Genital Psoriasis Area and Severity Index':ti,ab,kw OR 'modified genital PASI':ti,ab,kw OR GPSS:ti,ab,kw OR 'Genital Psoriasis Symptoms Scale':ti,ab,kw OR 'GPI-NRS':ti,ab,kw OR 'Genital Psoriasis Itch Numeric Rating Scale':ti,ab,kw OR 'genital itch Numeric Rating Scale':ti,ab,kw OR 'genital itch NRS':ti,ab,kw OR 'GenPs-Itch NRS':ti,ab,kw OR 'GenPs-SFQ':ti,ab,kw OR 'Genital Psoriasis Sexual Frequency Questionnaire':ti,ab,kw OR 'Sexual Frequency Questionnaire':ti,ab,kw OR GPSIS:ti,ab,kw OR 'Genital Psoriasis Sexual Impact Scale':ti,ab,kw OR 'Reasons for Sexual Impairment':ti,ab,kw OR 'reasons for sexual impairment':ti,ab,kw OR ( RSI:ti,ab,kw AND psoriasis:ti,ab,kw AND ( 'sexual impairment':ti,ab,kw OR 'sexual impact':ti,ab,kw OR 'sexual activity':ti,ab,kw OR 'sexual avoidance':ti,ab,kw ) ) ) |
| Embase via Embase.com | P2B: GenPs-specific or genital-site-specific instruments plus validation and psychometric terms. | To identify evidence on the development, validation, content validity, reliability, responsiveness, score interpretation, and psychometric status of genital psoriasis-specific or genital-site-specific instruments. | ( 'psoriasis'/exp OR psoriasis:ti,ab,kw OR 'genital psoriasis':ti,ab,kw OR 'anogenital psoriasis':ti,ab,kw OR 'perianal psoriasis':ti,ab,kw ) AND ( 'sPGA-G':ti,ab,kw OR 'static Physician Global Assessment of Genitalia':ti,ab,kw OR "static Physician's Global Assessment of Genitalia":ti,ab,kw OR 'genital PGA':ti,ab,kw OR 'genital Physician Global Assessment':ti,ab,kw OR "genital Physician's Global Assessment":ti,ab,kw OR 'physician global assessment of genital psoriasis':ti,ab,kw OR 'Patient Global Assessment-Genital':ti,ab,kw OR "Patient's Global Assessment of Genital Psoriasis":ti,ab,kw OR 'Patient Global Assessment of Genital Psoriasis':ti,ab,kw OR 'PatGA-Genital':ti,ab,kw OR mGPASI:ti,ab,kw OR 'modified Genital Psoriasis Area and Severity Index':ti,ab,kw OR 'modified genital PASI':ti,ab,kw OR GPSS:ti,ab,kw OR 'Genital Psoriasis Symptoms Scale':ti,ab,kw OR 'GPI-NRS':ti,ab,kw OR 'Genital Psoriasis Itch Numeric Rating Scale':ti,ab,kw OR 'genital itch Numeric Rating Scale':ti,ab,kw OR 'genital itch NRS':ti,ab,kw OR 'GenPs-Itch NRS':ti,ab,kw OR 'GenPs-SFQ':ti,ab,kw OR 'Genital Psoriasis Sexual Frequency Questionnaire':ti,ab,kw OR 'Sexual Frequency Questionnaire':ti,ab,kw OR GPSIS:ti,ab,kw OR 'Genital Psoriasis Sexual Impact Scale':ti,ab,kw OR 'Reasons for Sexual Impairment':ti,ab,kw OR 'reasons for sexual impairment':ti,ab,kw OR ( RSI:ti,ab,kw AND psoriasis:ti,ab,kw AND ( 'sexual impairment':ti,ab,kw OR 'sexual impact':ti,ab,kw OR 'sexual activity':ti,ab,kw OR 'sexual avoidance':ti,ab,kw ) ) ) AND ( psychometric*:ti,ab,kw OR validation:ti,ab,kw OR validated:ti,ab,kw OR validity:ti,ab,kw OR valid*:ti,ab,kw OR reliability:ti,ab,kw OR reliable:ti,ab,kw OR responsiveness:ti,ab,kw OR responsive:ti,ab,kw OR 'content validity':ti,ab,kw OR 'construct validity':ti,ab,kw OR 'structural validity':ti,ab,kw OR 'factor analysis':ti,ab,kw OR 'internal consistency':ti,ab,kw OR 'test-retest':ti,ab,kw OR 'cognitive debriefing':ti,ab,kw OR 'item generation':ti,ab,kw OR 'item reduction':ti,ab,kw OR 'score interpretation':ti,ab,kw OR interpretability:ti,ab,kw OR 'meaningful change':ti,ab,kw OR 'minimally important difference':ti,ab,kw OR 'patient-reported outcome':ti,ab,kw OR 'patient-reported outcomes':ti,ab,kw OR 'clinical outcome assessment':ti,ab,kw OR 'clinical outcome assessments':ti,ab,kw ) |
| Embase via Embase.com | P2C: Focused search for dermatology-, psoriasis-, skin-related sexuality, and generic comparator instruments. | To identify dermatology-related, psoriasis-related, skin-related sexuality, generic sexual function, sexual distress, genital self-image, intimacy, stigma, patient needs, and comparator instruments. | ( 'psoriasis'/exp OR psoriasis:ti,ab,kw OR dermatolog*:ti,ab,kw OR 'skin disease':ti,ab,kw OR 'skin diseases':ti,ab,kw OR 'chronic skin disease':ti,ab,kw OR 'chronic skin diseases':ti,ab,kw OR 'visible skin disease':ti,ab,kw OR 'visible skin diseases':ti,ab,kw OR 'cutaneous disease':ti,ab,kw OR 'cutaneous diseases':ti,ab,kw OR 'vulvar dermatosis':ti,ab,kw OR 'vulvar dermatoses':ti,ab,kw OR 'genital dermatosis':ti,ab,kw OR 'genital dermatoses':ti,ab,kw ) AND ( 'Female Sexual Function Index':ti,ab,kw OR FSFI:ti,ab,kw OR 'International Index of Erectile Function':ti,ab,kw OR IIEF:ti,ab,kw OR 'IIEF-5':ti,ab,kw OR 'Arizona Sexual Experience Scale':ti,ab,kw OR 'Arizona Sexual Experiences Scale':ti,ab,kw OR ASEX:ti,ab,kw OR 'Massachusetts General Hospital Sexual Functioning Questionnaire':ti,ab,kw OR 'MGH-SFQ':ti,ab,kw OR 'Female Sexual Distress Scale':ti,ab,kw OR 'Female Sexual Distress Scale-Revised':ti,ab,kw OR FSDS:ti,ab,kw OR 'FSDS-R':ti,ab,kw OR QualipsoSex:ti,ab,kw OR Qualipsosex:ti,ab,kw OR 'QualipsoSex questionnaire':ti,ab,kw OR 'Qualipsosex questionnaire':ti,ab,kw OR 'Skin-Related Sexual Life Questionnaire':ti,ab,kw OR 'Skin Related Sexual Life Questionnaire':ti,ab,kw OR SRSLQ:ti,ab,kw OR 'Sexual Quality of Life Questionnaire for Men':ti,ab,kw OR 'Sexual Quality of Life Questionnaire for use in Men':ti,ab,kw OR 'SQoL-M':ti,ab,kw OR 'Female Genital Self-Image Scale':ti,ab,kw OR FGSIS:ti,ab,kw OR 'Dermatology Life Quality Index':ti,ab,kw OR DLQI:ti,ab,kw OR 'DLQI item 9':ti,ab,kw OR 'sexual difficulties item':ti,ab,kw OR 'Skindex-16':ti,ab,kw OR 'Vulval Quality of Life Index':ti,ab,kw OR 'Vulvar Quality of Life Index':ti,ab,kw OR VQLI:ti,ab,kw OR VLQI:ti,ab,kw OR 'Psoriasis Disability Index':ti,ab,kw OR PDI:ti,ab,kw OR 'Psoriasis Internalized Stigma Scale':ti,ab,kw OR PISS:ti,ab,kw OR 'Relationship and Sexuality Scale':ti,ab,kw OR 'Revised Dyadic Adjustment Scale':ti,ab,kw OR 'Dermatologic Intimacy Scale':ti,ab,kw OR 'Perceived Stigmatization Questionnaire':ti,ab,kw OR 'Dysmorphic Concern Questionnaire':ti,ab,kw OR 'Patient Benefit Index':ti,ab,kw OR PBI:ti,ab,kw OR 'Patient Needs Questionnaire':ti,ab,kw OR 'Patient Benefit Questionnaire':ti,ab,kw OR 'Comprehensive Assessment of Psoriasis-related Patient-reported outcomes':ti,ab,kw ) AND ( 'sexual health':ti,ab,kw OR psychosexual:ti,ab,kw OR 'sexual function':ti,ab,kw OR 'sexual dysfunction':ti,ab,kw OR 'sexual distress':ti,ab,kw OR 'sexual quality of life':ti,ab,kw OR 'skin-related sexuality':ti,ab,kw OR sexuality:ti,ab,kw OR intimacy:ti,ab,kw OR 'body image':ti,ab,kw OR 'genital self-image':ti,ab,kw OR 'sexual self-image':ti,ab,kw OR stigma*:ti,ab,kw OR 'self-stigma':ti,ab,kw OR 'internalized stigma':ti,ab,kw OR shame:ti,ab,kw OR embarrassment:ti,ab,kw OR 'quality of life':ti,ab,kw OR QoL:ti,ab,kw OR psychometric*:ti,ab,kw OR valid*:ti,ab,kw OR validation:ti,ab,kw OR reliability:ti,ab,kw OR responsiveness:ti,ab,kw OR 'content validity':ti,ab,kw OR 'construct validity':ti,ab,kw OR 'structural validity':ti,ab,kw OR 'factor analysis':ti,ab,kw OR 'score interpretation':ti,ab,kw OR interpretability:ti,ab,kw OR 'meaningful change':ti,ab,kw OR 'minimally important difference':ti,ab,kw OR 'not relevant':ti,ab,kw OR 'not applicable':ti,ab,kw OR 'not sexually active':ti,ab,kw OR 'no sexual activity':ti,ab,kw OR 'no partner':ti,ab,kw OR 'partner status':ti,ab,kw OR 'current sexual activity':ti,ab,kw OR 'sexual orientation':ti,ab,kw OR 'gender identity':ti,ab,kw OR transgender:ti,ab,kw OR 'gender diverse':ti,ab,kw OR 'same-sex partnership':ti,ab,kw ) |
| Embase via Embase.com | P3A-2: Targeted construct-evidence search with measurement and interpretation terms. | To identify supplementary construct evidence that includes construct terms together with measurement, instruments, PROs, psychometrics, communication, clinical recognition, or patient needs. | ( 'psoriasis'/exp OR psoriasis:ti,ab,kw OR dermatolog*:ti,ab,kw OR 'skin disease':ti,ab,kw OR 'skin diseases':ti,ab,kw OR 'chronic skin disease':ti,ab,kw OR 'chronic skin diseases':ti,ab,kw OR 'visible skin disease':ti,ab,kw OR 'visible skin diseases':ti,ab,kw OR 'cutaneous disease':ti,ab,kw OR 'cutaneous diseases':ti,ab,kw OR 'vulvar dermatosis':ti,ab,kw OR 'vulvar dermatoses':ti,ab,kw OR 'genital dermatosis':ti,ab,kw OR 'genital dermatoses':ti,ab,kw ) AND ( psychosexual:ti,ab,kw OR 'sexual distress':ti,ab,kw OR 'sexual avoidance':ti,ab,kw OR 'sexual quality of life':ti,ab,kw OR 'sexual health':ti,ab,kw OR 'sexual function':ti,ab,kw OR 'sexual dysfunction':ti,ab,kw OR 'sexual impairment':ti,ab,kw OR 'sexual activity':ti,ab,kw OR 'sexual self-image':ti,ab,kw OR 'genital self-image':ti,ab,kw OR 'body image':ti,ab,kw OR 'appearance concern':ti,ab,kw OR 'appearance concerns':ti,ab,kw OR shame:ti,ab,kw OR embarrassment:ti,ab,kw OR stigma:ti,ab,kw OR stigmatization:ti,ab,kw OR stigmatisation:ti,ab,kw OR 'self-stigma':ti,ab,kw OR 'internalized stigma':ti,ab,kw OR 'internalised stigma':ti,ab,kw OR intimacy:ti,ab,kw OR 'intimate relationship':ti,ab,kw OR 'intimate relationships':ti,ab,kw OR 'relationship burden':ti,ab,kw OR 'partner evaluation':ti,ab,kw OR 'fear of rejection':ti,ab,kw OR avoidance:ti,ab,kw ) AND ( measurement:ti,ab,kw OR measure*:ti,ab,kw OR instrument*:ti,ab,kw OR questionnaire*:ti,ab,kw OR scale*:ti,ab,kw OR construct:ti,ab,kw OR constructs:ti,ab,kw OR 'patient-reported outcome':ti,ab,kw OR 'patient-reported outcomes':ti,ab,kw OR 'patient reported outcome':ti,ab,kw OR 'patient reported outcomes':ti,ab,kw OR psychometric*:ti,ab,kw OR valid*:ti,ab,kw OR validation:ti,ab,kw OR 'score interpretation':ti,ab,kw OR communication:ti,ab,kw OR 'clinical recognition':ti,ab,kw OR 'clinical visibility':ti,ab,kw OR 'patient needs':ti,ab,kw OR 'unmet needs':ti,ab,kw ) |
| Embase via Embase.com | P3B-1-revised: Patient-needs and care-context search. | To identify evidence on patient needs, unmet needs, patient preferences, patient–clinician communication, clinical recognition, and clinical visibility in psoriasis, dermatology, or genital-dermatology literature. | ( 'psoriasis'/exp OR psoriasis:ti,ab,kw OR dermatolog*:ti,ab,kw OR 'skin disease':ti,ab,kw OR 'skin diseases':ti,ab,kw OR 'chronic skin disease':ti,ab,kw OR 'chronic skin diseases':ti,ab,kw OR 'visible skin disease':ti,ab,kw OR 'visible skin diseases':ti,ab,kw OR 'cutaneous disease':ti,ab,kw OR 'cutaneous diseases':ti,ab,kw OR 'dermatologic disease':ti,ab,kw OR 'dermatologic diseases':ti,ab,kw OR 'vulvar dermatosis':ti,ab,kw OR 'vulvar dermatoses':ti,ab,kw OR 'genital dermatosis':ti,ab,kw OR 'genital dermatoses':ti,ab,kw OR ( ( genital:ti,ab,kw OR anogenital:ti,ab,kw OR perianal:ti,ab,kw OR vulvar:ti,ab,kw OR vulval:ti,ab,kw ) AND ( dermatolog*:ti,ab,kw OR dermatos*:ti,ab,kw OR cutaneous:ti,ab,kw OR skin:ti,ab,kw ) ) ) AND ( 'patient needs':ti,ab,kw OR 'unmet needs':ti,ab,kw OR 'patient need':ti,ab,kw OR 'unmet need':ti,ab,kw OR 'patient preference':ti,ab,kw OR 'patient preferences':ti,ab,kw OR 'Patient Benefit Index':ti,ab,kw OR 'Patient Needs Questionnaire':ti,ab,kw OR 'Patient Benefit Questionnaire':ti,ab,kw OR 'patient-clinician communication':ti,ab,kw OR 'clinician-patient communication':ti,ab,kw OR 'physician-patient communication':ti,ab,kw OR 'doctor-patient communication':ti,ab,kw OR 'difficult-to-communicate':ti,ab,kw OR 'preferred language':ti,ab,kw OR 'dermatology practice':ti,ab,kw OR 'dermatology visit':ti,ab,kw OR 'dermatology visits':ti,ab,kw OR 'routine dermatology':ti,ab,kw OR 'outpatient dermatology':ti,ab,kw OR 'clinical recognition':ti,ab,kw OR 'clinical visibility':ti,ab,kw ) AND ( genital:ti,ab,kw OR genitalia:ti,ab,kw OR anogenital:ti,ab,kw OR perianal:ti,ab,kw OR vulvar:ti,ab,kw OR vulval:ti,ab,kw OR sexual:ti,ab,kw OR psychosexual:ti,ab,kw OR 'sexual health':ti,ab,kw OR 'sexual function':ti,ab,kw OR 'sexual distress':ti,ab,kw OR 'sexual avoidance':ti,ab,kw OR intimacy:ti,ab,kw OR 'body image':ti,ab,kw OR 'genital self-image':ti,ab,kw OR stigma*:ti,ab,kw OR shame:ti,ab,kw OR embarrassment:ti,ab,kw OR 'quality of life':ti,ab,kw OR QoL:ti,ab,kw OR 'patient-reported outcome':ti,ab,kw OR 'patient-reported outcomes':ti,ab,kw OR communication:ti,ab,kw OR 'clinical recognition':ti,ab,kw OR 'clinical visibility':ti,ab,kw ) |
| Embase via Embase.com | P3A-1: Broad supplementary construct search. | To identify supplementary construct evidence related to sexual function versus sexual health, sexual distress, sexual avoidance, body image, genital self-image, sexual self-image, shame, stigma, intimacy, and relationship burden. | ( 'psoriasis'/exp OR psoriasis:ti,ab,kw OR dermatolog*:ti,ab,kw OR 'skin disease':ti,ab,kw OR 'skin diseases':ti,ab,kw OR 'chronic skin disease':ti,ab,kw OR 'chronic skin diseases':ti,ab,kw OR 'visible skin disease':ti,ab,kw OR 'visible skin diseases':ti,ab,kw OR 'cutaneous disease':ti,ab,kw OR 'cutaneous diseases':ti,ab,kw OR 'vulvar dermatosis':ti,ab,kw OR 'vulvar dermatoses':ti,ab,kw OR 'genital dermatosis':ti,ab,kw OR 'genital dermatoses':ti,ab,kw ) AND ( psychosexual:ti,ab,kw OR 'sexual distress':ti,ab,kw OR 'sexual avoidance':ti,ab,kw OR 'sexual quality of life':ti,ab,kw OR 'sexual health':ti,ab,kw OR 'sexual function':ti,ab,kw OR 'sexual dysfunction':ti,ab,kw OR 'sexual impairment':ti,ab,kw OR 'sexual activity':ti,ab,kw OR 'sexual self-image':ti,ab,kw OR 'genital self-image':ti,ab,kw OR 'body image':ti,ab,kw OR 'appearance concern':ti,ab,kw OR 'appearance concerns':ti,ab,kw OR shame:ti,ab,kw OR embarrassment:ti,ab,kw OR stigma:ti,ab,kw OR stigmatization:ti,ab,kw OR stigmatisation:ti,ab,kw OR 'self-stigma':ti,ab,kw OR 'internalized stigma':ti,ab,kw OR 'internalised stigma':ti,ab,kw OR intimacy:ti,ab,kw OR 'intimate relationship':ti,ab,kw OR 'intimate relationships':ti,ab,kw OR 'relationship burden':ti,ab,kw OR 'partner evaluation':ti,ab,kw OR 'fear of rejection':ti,ab,kw OR avoidance:ti,ab,kw ) |
| Embase via Embase.com | P3B-2: Sexual-health and genital-sensitive communication, and clinical-recognition search. | To identify evidence related to sexual-health or genital-sensitive communication, clinical recognition, documentation, referral, and follow-up care. | ( 'psoriasis'/exp OR psoriasis:ti,ab,kw OR dermatolog*:ti,ab,kw OR 'skin disease':ti,ab,kw OR 'skin diseases':ti,ab,kw OR 'chronic skin disease':ti,ab,kw OR 'chronic skin diseases':ti,ab,kw OR 'visible skin disease':ti,ab,kw OR 'visible skin diseases':ti,ab,kw ) AND ( 'sexual health discussion':ti,ab,kw OR 'sexual health communication':ti,ab,kw OR 'sexual communication':ti,ab,kw OR 'patient-clinician communication':ti,ab,kw OR 'clinician-patient communication':ti,ab,kw OR 'physician-patient communication':ti,ab,kw OR 'doctor-patient communication':ti,ab,kw OR 'sensitive topic':ti,ab,kw OR 'sensitive topics':ti,ab,kw OR 'sensitive issue':ti,ab,kw OR 'sensitive issues':ti,ab,kw OR 'difficult-to-communicate':ti,ab,kw OR 'preferred language':ti,ab,kw OR disclosure:ti,ab,kw OR disclose*:ti,ab,kw OR discuss*:ti,ab,kw OR conversation*:ti,ab,kw OR 'clinical recognition':ti,ab,kw OR 'clinical visibility':ti,ab,kw OR documentation:ti,ab,kw OR 'medical record':ti,ab,kw OR 'medical records':ti,ab,kw OR referral:ti,ab,kw OR 'follow-up care':ti,ab,kw OR triage:ti,ab,kw OR 'initial identification':ti,ab,kw ) AND ( genital:ti,ab,kw OR genitalia:ti,ab,kw OR 'genital psoriasis':ti,ab,kw OR 'genital involvement':ti,ab,kw OR anogenital:ti,ab,kw OR perianal:ti,ab,kw OR vulvar:ti,ab,kw OR vulval:ti,ab,kw OR psychosexual:ti,ab,kw OR 'sexual health':ti,ab,kw OR 'sexual function':ti,ab,kw OR 'sexual dysfunction':ti,ab,kw OR 'sexual distress':ti,ab,kw OR 'sexual avoidance':ti,ab,kw OR 'sexual activity':ti,ab,kw OR 'sexual quality of life':ti,ab,kw OR intimacy:ti,ab,kw OR 'intimate relationship':ti,ab,kw OR 'intimate relationships':ti,ab,kw OR 'genital self-image':ti,ab,kw OR 'sexual self-image':ti,ab,kw OR ( ( 'body image':ti,ab,kw OR stigma*:ti,ab,kw OR shame:ti,ab,kw OR embarrassment:ti,ab,kw ) AND ( sexual:ti,ab,kw OR genital:ti,ab,kw OR intimacy:ti,ab,kw OR psychosexual:ti,ab,kw ) ) ) |
| Embase via Embase.com | P3C-1: DLQI “not relevant/not applicable” and score-interpretation search. | To identify literature on DLQI item 9, not relevant/not applicable responses, inapplicable items, scoring, interpretability, content relevance, and score interpretation. | ( 'Dermatology Life Quality Index':ti,ab,kw OR DLQI:ti,ab,kw ) AND ( psoriasis:ti,ab,kw OR dermatolog*:ti,ab,kw OR 'skin disease':ti,ab,kw OR 'skin diseases':ti,ab,kw OR 'quality of life':ti,ab,kw ) AND ( 'not relevant':ti,ab,kw OR 'not applicable':ti,ab,kw OR inapplicable:ti,ab,kw OR 'response option':ti,ab,kw OR scoring:ti,ab,kw OR 'score interpretation':ti,ab,kw OR interpretability:ti,ab,kw OR 'content validity':ti,ab,kw OR 'content relevance':ti,ab,kw OR 'missing data':ti,ab,kw ) |
| Embase via Embase.com | P3C-2: Applicability and inapplicable-item search for sexual function scales. | To identify literature on inapplicable items, not sexually active status, no partner, response options, missing data, floor effects, sexual orientation, and gender-identity applicability in sexual function, sexual distress, and sexual QoL scales. | ( 'Female Sexual Function Index':ti,ab,kw OR FSFI:ti,ab,kw OR 'International Index of Erectile Function':ti,ab,kw OR IIEF:ti,ab,kw OR 'IIEF-5':ti,ab,kw OR 'Arizona Sexual Experience Scale':ti,ab,kw OR ASEX:ti,ab,kw OR 'Massachusetts General Hospital Sexual Functioning Questionnaire':ti,ab,kw OR 'MGH-SFQ':ti,ab,kw OR 'Female Sexual Distress Scale':ti,ab,kw OR FSDS:ti,ab,kw OR 'FSDS-R':ti,ab,kw OR 'Sexual Quality of Life Questionnaire for Men':ti,ab,kw OR 'SQoL-M':ti,ab,kw ) AND ( 'not applicable':ti,ab,kw OR 'not relevant':ti,ab,kw OR inapplicable:ti,ab,kw OR 'response option':ti,ab,kw OR 'not sexually active':ti,ab,kw OR 'no sexual activity':ti,ab,kw OR 'no partner':ti,ab,kw OR 'partner status':ti,ab,kw OR 'current sexual activity':ti,ab,kw OR 'missing data':ti,ab,kw OR 'floor effect':ti,ab,kw OR heterosexual:ti,ab,kw OR 'penetrative activity':ti,ab,kw OR 'sexual orientation':ti,ab,kw OR 'gender identity':ti,ab,kw ) AND ( validity:ti,ab,kw OR psychometric*:ti,ab,kw OR 'content validity':ti,ab,kw OR 'content relevance':ti,ab,kw OR 'score interpretation':ti,ab,kw OR scoring:ti,ab,kw OR interpretability:ti,ab,kw ) |
| Embase via Embase.com | P3C-3: Validity-theory and construct-misalignment search. | To identify methodological evidence on validity theory, construct underrepresentation, construct-irrelevant variance, and construct misalignment. | ( 'construct underrepresentation':ti,ab,kw OR 'construct-irrelevant variance':ti,ab,kw OR 'construct irrelevant variance':ti,ab,kw OR 'validity theory':ti,ab,kw OR ( 'construct validity':ti,ab,kw AND ( underrepresentation:ti,ab,kw OR 'irrelevant variance':ti,ab,kw ) ) ) AND ( 'patient-reported outcome':ti,ab,kw OR 'patient-reported outcomes':ti,ab,kw OR 'patient reported outcome':ti,ab,kw OR 'patient reported outcomes':ti,ab,kw OR 'clinical outcome assessment':ti,ab,kw OR 'clinical outcome assessments':ti,ab,kw OR questionnaire*:ti,ab,kw OR scale*:ti,ab,kw OR instrument*:ti,ab,kw OR measurement:ti,ab,kw OR psychometric*:ti,ab,kw ) |
| Embase via Embase.com | SOGI/LGBTQ+ applicability and underrepresentation supplementary search. | To identify evidence on SOGI/LGBTQ+ population applicability, communication needs, underrepresentation, and measurement boundaries in psoriasis, dermatology, or genital-dermatology literature. | ( psoriasis:ti,ab,kw OR dermatolog*:ti,ab,kw OR 'skin disease':ti,ab,kw OR 'skin diseases':ti,ab,kw OR 'chronic skin disease':ti,ab,kw OR 'chronic skin diseases':ti,ab,kw OR 'visible skin disease':ti,ab,kw OR 'visible skin diseases':ti,ab,kw OR 'cutaneous disease':ti,ab,kw OR 'cutaneous diseases':ti,ab,kw OR 'vulvar dermatosis':ti,ab,kw OR 'vulvar dermatoses':ti,ab,kw OR 'genital dermatosis':ti,ab,kw OR 'genital dermatoses':ti,ab,kw ) AND ( LGBT*:ti,ab,kw OR LGBTQ*:ti,ab,kw OR 'sexual minority':ti,ab,kw OR 'sexual minorities':ti,ab,kw OR 'gender minority':ti,ab,kw OR 'gender minorities':ti,ab,kw OR 'sexual orientation':ti,ab,kw OR 'gender identity':ti,ab,kw OR transgender:ti,ab,kw OR nonbinary:ti,ab,kw OR 'non-binary':ti,ab,kw OR 'gender diverse':ti,ab,kw OR 'same-sex partner':ti,ab,kw OR 'same-sex partnership':ti,ab,kw ) AND ( 'sexual health':ti,ab,kw OR 'sexual distress':ti,ab,kw OR 'sexual function':ti,ab,kw OR 'body image':ti,ab,kw OR 'genital self-image':ti,ab,kw OR stigma:ti,ab,kw OR communication:ti,ab,kw OR 'patient-reported outcome':ti,ab,kw OR 'patient-reported outcomes':ti,ab,kw OR 'clinical outcome assessment':ti,ab,kw OR measurement:ti,ab,kw OR validity:ti,ab,kw OR applicability:ti,ab,kw OR underrepresentation:ti,ab,kw OR 'measurement boundary':ti,ab,kw OR 'measurement boundaries':ti,ab,kw ) |
| Embase via Embase.com | Search implementation summary |  | Main search: P1A OR P1B OR P2A OR P2B OR P2C OR P3A-2 OR P3B-1-revised.  Targeted supplementary searches: P3A-1, P3B-2, P3C-1, P3C-2, P3C-3, and the SOGI/LGBTQ+ applicability/underrepresentation search were run and recorded separately and were not included in the main search set. |
|  | | | |
| APA PsycINFO via Ovid | P1A: Core disease–site search. | To identify core literature on genital, anogenital, perianal, or genital-relevant special-site psoriasis. | (XB psoriasis OR XB "psoriasis vulgaris" OR XB "plaque psoriasis") AND (XB genital* OR XB genitalia OR XB "genital involvement" OR XB "genital lesion" OR XB "genital lesions" OR XB "genital psoriasis" OR XB anogenital OR XB "anogenital psoriasis" OR XB perigenital OR XB vulvar OR XB vulval OR XB vulva OR XB labia OR XB labial OR XB penile OR XB penis OR XB penoscrotal OR XB glans OR XB foreskin OR XB prepuce OR XB preputial OR XB scrotal OR XB scrotum OR XB perianal OR XB "perianal psoriasis" OR XB perineal OR XB perineum OR XB groin OR XB inguinal OR XB "inguinal crease" OR XB "inguinal creases" OR XB intergluteal OR XB "intergluteal fold" OR XB "intergluteal cleft" OR XB "gluteal cleft" OR XB "natal cleft" OR XB "inverse psoriasis" OR XB "intertriginous psoriasis" OR XB "flexural psoriasis") |
| APA PsycINFO via Ovid | P1B: Disease–site, outcome, measurement, communication, and clinical-recognition search. | To identify genital-relevant psoriasis literature on symptoms, sexual health, sexual impact, QoL, PROs, measurement, patient needs, communication, documentation, referral, follow-up care, or clinical recognition. | (XB psoriasis OR XB "psoriasis vulgaris" OR XB "plaque psoriasis") AND (XB genital* OR XB genitalia OR XB "genital involvement" OR XB "genital lesion" OR XB "genital lesions" OR XB "genital psoriasis" OR XB anogenital OR XB "anogenital psoriasis" OR XB perigenital OR XB vulvar OR XB vulval OR XB vulva OR XB labia OR XB labial OR XB penile OR XB penis OR XB penoscrotal OR XB glans OR XB foreskin OR XB prepuce OR XB preputial OR XB scrotal OR XB scrotum OR XB perianal OR XB "perianal psoriasis" OR XB perineal OR XB perineum OR XB groin OR XB inguinal OR XB "inguinal crease" OR XB "inguinal creases" OR XB intergluteal OR XB "intergluteal fold" OR XB "intergluteal cleft" OR XB "gluteal cleft" OR XB "natal cleft" OR XB "inverse psoriasis" OR XB "intertriginous psoriasis" OR XB "flexural psoriasis") AND (XB symptom* OR XB itch* OR XB prurit* OR XB pain OR XB painful OR XB burning OR XB fissur* OR XB discomfort OR XB friction OR XB "sexual health" OR XB psychosexual OR XB "sexual function" OR XB "sexual dysfunction" OR XB "sexual distress" OR XB "sexual activity" OR XB "sexual frequency" OR XB "sexual impact" OR XB "sexual impairment" OR XB "sexual avoidance" OR XB intimacy OR XB "intimate relationship" OR XB "intimate relationships" OR XB "quality of life" OR XB QoL OR XB "sexual quality of life" OR XB "patient-reported outcome" OR XB "patient-reported outcomes" OR XB "patient reported outcome" OR XB "patient reported outcomes" OR XB questionnaire* OR XB scale* OR XB instrument* OR XB measure* OR XB measurement OR XB assessment* OR XB endpoint* OR XB psychometric* OR XB valid* OR XB validation OR XB reliability OR XB responsiveness OR XB "content validity" OR XB "construct validity" OR XB "score interpretation" OR XB "patient needs" OR XB "unmet needs" OR XB "treatment benefit" OR XB "treatment benefits" OR XB communication OR XB discuss* OR XB conversation* OR XB "clinical recognition" OR XB "clinical visibility" OR XB documentation OR XB referral OR XB "follow-up care") |
| APA PsycINFO via Ovid | P2A: GenPs-specific or genital-site-specific instrument search. | To identify genital psoriasis-specific or genital-site-specific instrument names, endpoint names, study-specific measurement paths, trial outcome terms, and source reports. | (XB psoriasis OR XB "genital psoriasis" OR XB "anogenital psoriasis" OR XB "perianal psoriasis") AND (XB "sPGA-G" OR XB "static Physician's Global Assessment of Genitalia" OR XB "static Physician Global Assessment of Genitalia" OR XB "genital PGA" OR XB "genital Physician's Global Assessment" OR XB "genital Physician Global Assessment" OR XB "physician global assessment of genital psoriasis" OR XB "Patient Global Assessment-Genital" OR XB "Patient's Global Assessment of Genital Psoriasis" OR XB "Patient Global Assessment of Genital Psoriasis" OR XB "PatGA-Genital" OR XB mGPASI OR XB "modified Genital Psoriasis Area and Severity Index" OR XB "modified genital PASI" OR XB GPSS OR XB "Genital Psoriasis Symptoms Scale" OR XB "GPI-NRS" OR XB "Genital Psoriasis Itch Numeric Rating Scale" OR XB "genital itch Numeric Rating Scale" OR XB "genital itch NRS" OR XB "GenPs-Itch NRS" OR XB "GenPs-SFQ" OR XB "Genital Psoriasis Sexual Frequency Questionnaire" OR XB "Sexual Frequency Questionnaire" OR XB GPSIS OR XB "Genital Psoriasis Sexual Impact Scale" OR XB "Reasons for Sexual Impairment" OR XB "reasons for sexual impairment" OR (XB RSI AND XB psoriasis AND (XB "sexual impairment" OR XB "sexual impact" OR XB "sexual activity" OR XB "sexual avoidance"))) |
| APA PsycINFO via Ovid | P2B: GenPs-specific or genital-site-specific instruments plus validation and psychometric terms. | To identify evidence on the development, validation, content validity, reliability, responsiveness, score interpretation, and psychometric status of genital psoriasis-specific or genital-site-specific instruments. | (XB psoriasis OR XB "genital psoriasis" OR XB "anogenital psoriasis" OR XB "perianal psoriasis") AND (XB "sPGA-G" OR XB "static Physician's Global Assessment of Genitalia" OR XB "static Physician Global Assessment of Genitalia" OR XB "genital PGA" OR XB "genital Physician's Global Assessment" OR XB "genital Physician Global Assessment" OR XB "physician global assessment of genital psoriasis" OR XB "Patient Global Assessment-Genital" OR XB "Patient's Global Assessment of Genital Psoriasis" OR XB "Patient Global Assessment of Genital Psoriasis" OR XB "PatGA-Genital" OR XB mGPASI OR XB "modified Genital Psoriasis Area and Severity Index" OR XB "modified genital PASI" OR XB GPSS OR XB "Genital Psoriasis Symptoms Scale" OR XB "GPI-NRS" OR XB "Genital Psoriasis Itch Numeric Rating Scale" OR XB "genital itch Numeric Rating Scale" OR XB "genital itch NRS" OR XB "GenPs-Itch NRS" OR XB "GenPs-SFQ" OR XB "Genital Psoriasis Sexual Frequency Questionnaire" OR XB "Sexual Frequency Questionnaire" OR XB GPSIS OR XB "Genital Psoriasis Sexual Impact Scale" OR XB "Reasons for Sexual Impairment" OR XB "reasons for sexual impairment" OR (XB RSI AND XB psoriasis AND (XB "sexual impairment" OR XB "sexual impact" OR XB "sexual activity" OR XB "sexual avoidance"))) AND (XB psychometric* OR XB validation OR XB validated OR XB validity OR XB valid* OR XB reliability OR XB reliable OR XB responsiveness OR XB responsive OR XB "content validity" OR XB "construct validity" OR XB "structural validity" OR XB "factor analysis" OR XB "internal consistency" OR XB "test-retest" OR XB "cognitive debriefing" OR XB "item generation" OR XB "item reduction" OR XB "score interpretation" OR XB interpretability OR XB "meaningful change" OR XB "minimally important difference" OR XB "patient-reported outcome" OR XB "patient-reported outcomes" OR XB "clinical outcome assessment" OR XB "clinical outcome assessments") |
| APA PsycINFO via Ovid | P2C: Focused search for dermatology-, psoriasis-, skin-related sexuality, and generic comparator instruments. | To identify dermatology-related, psoriasis-related, skin-related sexuality, generic sexual function, sexual distress, genital self-image, intimacy, stigma, patient-needs, and comparator instruments. | (XB psoriasis OR XB dermatolog* OR XB "skin disease" OR XB "skin diseases" OR XB "chronic skin disease" OR XB "chronic skin diseases" OR XB "visible skin disease" OR XB "visible skin diseases" OR XB "cutaneous disease" OR XB "cutaneous diseases" OR XB "vulvar dermatosis" OR XB "vulvar dermatoses" OR XB "genital dermatosis" OR XB "genital dermatoses") AND (XB "Female Sexual Function Index" OR XB FSFI OR XB "International Index of Erectile Function" OR XB IIEF OR XB "IIEF-5" OR XB "Arizona Sexual Experience Scale" OR XB "Arizona Sexual Experiences Scale" OR XB ASEX OR XB "Massachusetts General Hospital Sexual Functioning Questionnaire" OR XB "MGH-SFQ" OR XB "Female Sexual Distress Scale" OR XB "Female Sexual Distress Scale-Revised" OR XB FSDS OR XB "FSDS-R" OR XB QualipsoSex OR XB Qualipsosex OR XB "QualipsoSex questionnaire" OR XB "Qualipsosex questionnaire" OR XB "Skin-Related Sexual Life Questionnaire" OR XB "Skin Related Sexual Life Questionnaire" OR XB SRSLQ OR XB "Sexual Quality of Life Questionnaire for Men" OR XB "Sexual Quality of Life Questionnaire for use in Men" OR XB "SQoL-M" OR XB "Female Genital Self-Image Scale" OR XB FGSIS OR XB "Dermatology Life Quality Index" OR XB DLQI OR XB "DLQI item 9" OR XB "sexual difficulties item" OR XB "Skindex-16" OR XB "Vulval Quality of Life Index" OR XB "Vulvar Quality of Life Index" OR XB VQLI OR XB VLQI OR XB "Psoriasis Disability Index" OR XB PDI OR XB "Psoriasis Internalized Stigma Scale" OR XB PISS OR XB "Relationship and Sexuality Scale" OR XB "Revised Dyadic Adjustment Scale" OR XB "Dermatologic Intimacy Scale" OR XB "Perceived Stigmatization Questionnaire" OR XB "Dysmorphic Concern Questionnaire" OR XB "Patient Benefit Index" OR XB PBI OR XB "Patient Needs Questionnaire" OR XB "Patient Benefit Questionnaire" OR XB "Comprehensive Assessment of Psoriasis-related Patient-reported outcomes") AND (XB "sexual health" OR XB psychosexual OR XB "sexual function" OR XB "sexual dysfunction" OR XB "sexual distress" OR XB "sexual quality of life" OR XB "skin-related sexuality" OR XB sexuality OR XB intimacy OR XB "body image" OR XB "genital self-image" OR XB "sexual self-image" OR XB stigma* OR XB "self-stigma" OR XB "internalized stigma" OR XB shame OR XB embarrassment OR XB "quality of life" OR XB QoL OR XB psychometric* OR XB valid* OR XB validation OR XB reliability OR XB responsiveness OR XB "content validity" OR XB "construct validity" OR XB "structural validity" OR XB "factor analysis" OR XB "score interpretation" OR XB interpretability OR XB "meaningful change" OR XB "minimally important difference" OR XB "not relevant" OR XB "not applicable" OR XB "not sexually active" OR XB "no sexual activity" OR XB "no partner" OR XB "partner status" OR XB "current sexual activity" OR XB "sexual orientation" OR XB "gender identity" OR XB transgender OR XB "gender diverse" OR XB "same-sex partnership") |
| APA PsycINFO via Ovid | P3A-2: Targeted construct-evidence search with measurement and interpretation terms. | To identify supplementary construct evidence that includes construct terms together with measurement, instruments, PROs, psychometrics, communication, clinical recognition, or patient needs. | (XB psoriasis OR XB dermatolog* OR XB "skin disease" OR XB "skin diseases" OR XB "chronic skin disease" OR XB "chronic skin diseases" OR XB "visible skin disease" OR XB "visible skin diseases" OR XB "cutaneous disease" OR XB "cutaneous diseases" OR XB "vulvar dermatosis" OR XB "vulvar dermatoses" OR XB "genital dermatosis" OR XB "genital dermatoses") AND (XB psychosexual OR XB "sexual distress" OR XB "sexual avoidance" OR XB "sexual quality of life" OR XB "sexual health" OR XB "sexual function" OR XB "sexual dysfunction" OR XB "sexual impairment" OR XB "sexual activity" OR XB "sexual self-image" OR XB "genital self-image" OR XB "body image" OR XB "appearance concern" OR XB "appearance concerns" OR XB shame OR XB embarrassment OR XB stigma OR XB stigmatization OR XB stigmatisation OR XB "self-stigma" OR XB "internalized stigma" OR XB "internalised stigma" OR XB intimacy OR XB "intimate relationship" OR XB "intimate relationships" OR XB "relationship burden" OR XB "partner evaluation" OR XB "fear of rejection" OR XB avoidance) AND (XB measurement OR XB measure* OR XB instrument* OR XB questionnaire* OR XB scale* OR XB construct OR XB constructs OR XB "patient-reported outcome" OR XB "patient-reported outcomes" OR XB "patient reported outcome" OR XB "patient reported outcomes" OR XB psychometric* OR XB valid* OR XB validation OR XB "score interpretation" OR XB communication OR XB "clinical recognition" OR XB "clinical visibility" OR XB "patient needs" OR XB "unmet needs") |
| APA PsycINFO via Ovid | P3B-1-revised: Patient-needs and care-context search. | To identify evidence on patient needs, unmet needs, patient preferences, patient–clinician communication, clinical recognition, and clinical visibility in psoriasis, dermatology, or genital-dermatology literature. | (XB psoriasis OR XB dermatolog* OR XB "skin disease" OR XB "skin diseases" OR XB "chronic skin disease" OR XB "chronic skin diseases" OR XB "visible skin disease" OR XB "visible skin diseases" OR XB "cutaneous disease" OR XB "cutaneous diseases" OR XB "dermatologic disease" OR XB "dermatologic diseases" OR XB "vulvar dermatosis" OR XB "vulvar dermatoses" OR XB "genital dermatosis" OR XB "genital dermatoses" OR ((XB genital OR XB anogenital OR XB perianal OR XB vulvar OR XB vulval) AND (XB dermatolog* OR XB dermatos* OR XB cutaneous OR XB skin))) AND (XB "patient needs" OR XB "unmet needs" OR XB "patient need" OR XB "unmet need" OR XB "patient preference" OR XB "patient preferences" OR XB "Patient Benefit Index" OR XB "Patient Needs Questionnaire" OR XB "Patient Benefit Questionnaire" OR XB "patient-clinician communication" OR XB "clinician-patient communication" OR XB "physician-patient communication" OR XB "doctor-patient communication" OR XB "difficult-to-communicate" OR XB "preferred language" OR XB "dermatology practice" OR XB "dermatology visit" OR XB "dermatology visits" OR XB "routine dermatology" OR XB "outpatient dermatology" OR XB "clinical recognition" OR XB "clinical visibility") AND (XB genital OR XB genitalia OR XB anogenital OR XB perianal OR XB vulvar OR XB vulval OR XB sexual OR XB psychosexual OR XB "sexual health" OR XB "sexual function" OR XB "sexual distress" OR XB "sexual avoidance" OR XB intimacy OR XB "body image" OR XB "genital self-image" OR XB stigma* OR XB shame OR XB embarrassment OR XB "quality of life" OR XB QoL OR XB "patient-reported outcome" OR XB "patient-reported outcomes" OR XB communication OR XB "clinical recognition" OR XB "clinical visibility") |
| APA PsycINFO via Ovid | P3A-1: Broad supplementary construct search. | To identify supplementary construct evidence related to sexual function versus sexual health, sexual distress, sexual avoidance, body image, genital self-image, sexual self-image, shame, stigma, intimacy, and relationship burden. | (XB psoriasis OR XB dermatolog* OR XB "skin disease" OR XB "skin diseases" OR XB "chronic skin disease" OR XB "chronic skin diseases" OR XB "visible skin disease" OR XB "visible skin diseases" OR XB "cutaneous disease" OR XB "cutaneous diseases" OR XB "vulvar dermatosis" OR XB "vulvar dermatoses" OR XB "genital dermatosis" OR XB "genital dermatoses") AND (XB psychosexual OR XB "sexual distress" OR XB "sexual avoidance" OR XB "sexual quality of life" OR XB "sexual health" OR XB "sexual function" OR XB "sexual dysfunction" OR XB "sexual impairment" OR XB "sexual activity" OR XB "sexual self-image" OR XB "genital self-image" OR XB "body image" OR XB "appearance concern" OR XB "appearance concerns" OR XB shame OR XB embarrassment OR XB stigma OR XB stigmatization OR XB stigmatisation OR XB "self-stigma" OR XB "internalized stigma" OR XB "internalised stigma" OR XB intimacy OR XB "intimate relationship" OR XB "intimate relationships" OR XB "relationship burden" OR XB "partner evaluation" OR XB "fear of rejection" OR XB avoidance) |
| APA PsycINFO via Ovid | P3B-2: Sexual-health and genital-sensitive communication, and clinical-recognition search. | To identify evidence related to sexual-health or genital-sensitive communication, clinical recognition, documentation, referral, and follow-up care. | (XB psoriasis OR XB dermatolog* OR XB "skin disease" OR XB "skin diseases" OR XB "chronic skin disease" OR XB "chronic skin diseases" OR XB "visible skin disease" OR XB "visible skin diseases") AND (XB "sexual health discussion" OR XB "sexual health communication" OR XB "sexual communication" OR XB "patient-clinician communication" OR XB "clinician-patient communication" OR XB "physician-patient communication" OR XB "doctor-patient communication" OR XB "sensitive topic" OR XB "sensitive topics" OR XB "sensitive issue" OR XB "sensitive issues" OR XB "difficult-to-communicate" OR XB "preferred language" OR XB disclosure OR XB disclose* OR XB discuss* OR XB conversation* OR XB "clinical recognition" OR XB "clinical visibility" OR XB documentation OR XB "medical record" OR XB "medical records" OR XB referral OR XB "follow-up care" OR XB triage OR XB "initial identification") AND (XB genital OR XB genitalia OR XB "genital psoriasis" OR XB "genital involvement" OR XB anogenital OR XB perianal OR XB vulvar OR XB vulval OR XB psychosexual OR XB "sexual health" OR XB "sexual function" OR XB "sexual dysfunction" OR XB "sexual distress" OR XB "sexual avoidance" OR XB "sexual activity" OR XB "sexual quality of life" OR XB intimacy OR XB "intimate relationship" OR XB "intimate relationships" OR XB "genital self-image" OR XB "sexual self-image" OR ((XB "body image" OR XB stigma* OR XB shame OR XB embarrassment) AND (XB sexual OR XB genital OR XB intimacy OR XB psychosexual))) |
| APA PsycINFO via Ovid | P3C-1: DLQI “not relevant/not applicable” and score-interpretation search. | To identify literature on DLQI item 9, not relevant/not applicable responses, inapplicable items, scoring, interpretability, content relevance, and score interpretation. | (XB "Dermatology Life Quality Index" OR XB DLQI) AND (XB psoriasis OR XB dermatolog* OR XB "skin disease" OR XB "skin diseases" OR XB "quality of life") AND (XB "not relevant" OR XB "not applicable" OR XB inapplicable OR XB "response option" OR XB scoring OR XB "score interpretation" OR XB interpretability OR XB "content validity" OR XB "content relevance" OR XB "missing data") |
| APA PsycINFO via Ovid | P3C-2: Applicability and inapplicable-item search for sexual function scales. | To identify literature on inapplicable items, not sexually active status, no partner, response options, missing data, floor effects, sexual orientation, and gender-identity applicability in sexual function, sexual distress, and sexual QoL scales. | (XB "Female Sexual Function Index" OR XB FSFI OR XB "International Index of Erectile Function" OR XB IIEF OR XB "IIEF-5" OR XB "Arizona Sexual Experience Scale" OR XB ASEX OR XB "Massachusetts General Hospital Sexual Functioning Questionnaire" OR XB "MGH-SFQ" OR XB "Female Sexual Distress Scale" OR XB FSDS OR XB "FSDS-R" OR XB "Sexual Quality of Life Questionnaire for Men" OR XB "SQoL-M") AND (XB "not applicable" OR XB "not relevant" OR XB inapplicable OR XB "response option" OR XB "not sexually active" OR XB "no sexual activity" OR XB "no partner" OR XB "partner status" OR XB "current sexual activity" OR XB "missing data" OR XB "floor effect" OR XB heterosexual OR XB "penetrative activity" OR XB "sexual orientation" OR XB "gender identity") AND (XB validity OR XB psychometric* OR XB "content validity" OR XB "content relevance" OR XB "score interpretation" OR XB scoring OR XB interpretability) |
| APA PsycINFO via Ovid | P3C-3: Validity-theory and construct-misalignment search. | To identify methodological evidence on validity theory, construct underrepresentation, construct-irrelevant variance, and construct misalignment. | (XB "construct underrepresentation" OR XB "construct-irrelevant variance" OR XB "construct irrelevant variance" OR XB "validity theory" OR (XB "construct validity" AND (XB underrepresentation OR XB "irrelevant variance"))) AND (XB "patient-reported outcome" OR XB "patient-reported outcomes" OR XB "patient reported outcome" OR XB "patient reported outcomes" OR XB "clinical outcome assessment" OR XB "clinical outcome assessments" OR XB questionnaire* OR XB scale* OR XB instrument* OR XB measurement OR XB psychometric*) |
| APA PsycINFO via Ovid | SOGI/LGBTQ+ applicability and underrepresentation supplementary search. | To identify evidence on SOGI/LGBTQ+ population applicability, communication needs, underrepresentation, and measurement boundaries in psoriasis, dermatology, or genital-dermatology literature. | (XB psoriasis OR XB dermatolog* OR XB "skin disease" OR XB "skin diseases" OR XB "chronic skin disease" OR XB "chronic skin diseases" OR XB "visible skin disease" OR XB "visible skin diseases" OR XB "cutaneous disease" OR XB "cutaneous diseases" OR XB "vulvar dermatosis" OR XB "vulvar dermatoses" OR XB "genital dermatosis" OR XB "genital dermatoses") AND (XB LGBT* OR XB LGBTQ* OR XB "sexual minority" OR XB "sexual minorities" OR XB "gender minority" OR XB "gender minorities" OR XB "sexual orientation" OR XB "gender identity" OR XB transgender OR XB nonbinary OR XB "non-binary" OR XB "gender diverse" OR XB "same-sex partner" OR XB "same-sex partnership") AND (XB "sexual health" OR XB "sexual distress" OR XB "sexual function" OR XB "body image" OR XB "genital self-image" OR XB stigma OR XB communication OR XB "patient-reported outcome" OR XB "patient-reported outcomes" OR XB "clinical outcome assessment" OR XB measurement OR XB validity OR XB applicability OR XB underrepresentation OR XB "measurement boundary" OR XB "measurement boundaries") |
| APA PsycINFO via Ovid | Search implementation summary |  | Main search: P1A OR P1B OR P2A OR P2B OR P2C OR P3A-2 OR P3B-1-revised.  Targeted supplementary searches: P3A-1, P3B-2, P3C-1, P3C-2, P3C-3, and the SOGI/LGBTQ+ applicability/underrepresentation search were run and recorded separately and were not included in the main search set. |
|  | | | |
| CINAHL via EBSCO Research Platform | P1A: Core disease–site search. | To identify core literature on genital, anogenital, perianal, or genital-relevant special-site psoriasis. | (MH "Psoriasis+" OR TI psoriasis OR AB psoriasis OR TI "psoriasis vulgaris" OR AB "psoriasis vulgaris" OR TI "plaque psoriasis" OR AB "plaque psoriasis") AND (TI genital* OR AB genital* OR TI genitalia OR AB genitalia OR TI "genital involvement" OR AB "genital involvement" OR TI "genital lesion" OR AB "genital lesion" OR TI "genital lesions" OR AB "genital lesions" OR TI "genital psoriasis" OR AB "genital psoriasis" OR TI anogenital OR AB anogenital OR TI "anogenital psoriasis" OR AB "anogenital psoriasis" OR TI perigenital OR AB perigenital OR TI vulvar OR AB vulvar OR TI vulval OR AB vulval OR TI vulva OR AB vulva OR TI labia OR AB labia OR TI labial OR AB labial OR TI penile OR AB penile OR TI penis OR AB penis OR TI penoscrotal OR AB penoscrotal OR TI glans OR AB glans OR TI foreskin OR AB foreskin OR TI prepuce OR AB prepuce OR TI preputial OR AB preputial OR TI scrotal OR AB scrotal OR TI scrotum OR AB scrotum OR TI perianal OR AB perianal OR TI "perianal psoriasis" OR AB "perianal psoriasis" OR TI perineal OR AB perineal OR TI perineum OR AB perineum OR TI groin OR AB groin OR TI inguinal OR AB inguinal OR TI "inguinal crease" OR AB "inguinal crease" OR TI "inguinal creases" OR AB "inguinal creases" OR TI intergluteal OR AB intergluteal OR TI "intergluteal fold" OR AB "intergluteal fold" OR TI "intergluteal cleft" OR AB "intergluteal cleft" OR TI "gluteal cleft" OR AB "gluteal cleft" OR TI "natal cleft" OR AB "natal cleft" OR TI "inverse psoriasis" OR AB "inverse psoriasis" OR TI "intertriginous psoriasis" OR AB "intertriginous psoriasis" OR TI "flexural psoriasis" OR AB "flexural psoriasis") |
| CINAHL via EBSCO Research Platform | P1B: Disease–site, outcome, measurement, communication, and clinical-recognition search. | To identify genital-relevant psoriasis literature on symptoms, sexual health, sexual impact, QoL, PROs, measurement, patient needs, communication, documentation, referral, follow-up care, or clinical recognition. | (MH "Psoriasis+" OR TI psoriasis OR AB psoriasis OR TI "psoriasis vulgaris" OR AB "psoriasis vulgaris" OR TI "plaque psoriasis" OR AB "plaque psoriasis") AND (TI genital* OR AB genital* OR TI genitalia OR AB genitalia OR TI "genital involvement" OR AB "genital involvement" OR TI "genital lesion" OR AB "genital lesion" OR TI "genital lesions" OR AB "genital lesions" OR TI "genital psoriasis" OR AB "genital psoriasis" OR TI anogenital OR AB anogenital OR TI "anogenital psoriasis" OR AB "anogenital psoriasis" OR TI perigenital OR AB perigenital OR TI vulvar OR AB vulvar OR TI vulval OR AB vulval OR TI vulva OR AB vulva OR TI labia OR AB labia OR TI labial OR AB labial OR TI penile OR AB penile OR TI penis OR AB penis OR TI penoscrotal OR AB penoscrotal OR TI glans OR AB glans OR TI foreskin OR AB foreskin OR TI prepuce OR AB prepuce OR TI preputial OR AB preputial OR TI scrotal OR AB scrotal OR TI scrotum OR AB scrotum OR TI perianal OR AB perianal OR TI "perianal psoriasis" OR AB "perianal psoriasis" OR TI perineal OR AB perineal OR TI perineum OR AB perineum OR TI groin OR AB groin OR TI inguinal OR AB inguinal OR TI "inguinal crease" OR AB "inguinal crease" OR TI "inguinal creases" OR AB "inguinal creases" OR TI intergluteal OR AB intergluteal OR TI "intergluteal fold" OR AB "intergluteal fold" OR TI "intergluteal cleft" OR AB "intergluteal cleft" OR TI "gluteal cleft" OR AB "gluteal cleft" OR TI "natal cleft" OR AB "natal cleft" OR TI "inverse psoriasis" OR AB "inverse psoriasis" OR TI "intertriginous psoriasis" OR AB "intertriginous psoriasis" OR TI "flexural psoriasis" OR AB "flexural psoriasis") AND (TI symptom* OR AB symptom* OR TI itch* OR AB itch* OR TI prurit* OR AB prurit* OR TI pain OR AB pain OR TI painful OR AB painful OR TI burning OR AB burning OR TI fissur* OR AB fissur* OR TI discomfort OR AB discomfort OR TI friction OR AB friction OR TI "sexual health" OR AB "sexual health" OR TI psychosexual OR AB psychosexual OR TI "sexual function" OR AB "sexual function" OR TI "sexual dysfunction" OR AB "sexual dysfunction" OR TI "sexual distress" OR AB "sexual distress" OR TI "sexual activity" OR AB "sexual activity" OR TI "sexual frequency" OR AB "sexual frequency" OR TI "sexual impact" OR AB "sexual impact" OR TI "sexual impairment" OR AB "sexual impairment" OR TI "sexual avoidance" OR AB "sexual avoidance" OR TI intimacy OR AB intimacy OR TI "intimate relationship" OR AB "intimate relationship" OR TI "intimate relationships" OR AB "intimate relationships" OR MH "Quality of Life+" OR TI "quality of life" OR AB "quality of life" OR TI QoL OR AB QoL OR TI "sexual quality of life" OR AB "sexual quality of life" OR TI "patient-reported outcome" OR AB "patient-reported outcome" OR TI "patient-reported outcomes" OR AB "patient-reported outcomes" OR TI "patient reported outcome" OR AB "patient reported outcome" OR TI "patient reported outcomes" OR AB "patient reported outcomes" OR TI questionnaire* OR AB questionnaire* OR TI scale* OR AB scale* OR TI instrument* OR AB instrument* OR TI measure* OR AB measure* OR TI measurement OR AB measurement OR TI assessment* OR AB assessment* OR TI endpoint* OR AB endpoint* OR TI psychometric* OR AB psychometric* OR TI valid* OR AB valid* OR TI validation OR AB validation OR TI reliability OR AB reliability OR TI responsiveness OR AB responsiveness OR TI "content validity" OR AB "content validity" OR TI "construct validity" OR AB "construct validity" OR TI "score interpretation" OR AB "score interpretation" OR TI "patient needs" OR AB "patient needs" OR TI "unmet needs" OR AB "unmet needs" OR TI "treatment benefit" OR AB "treatment benefit" OR TI "treatment benefits" OR AB "treatment benefits" OR TI communication OR AB communication OR TI discuss* OR AB discuss* OR TI conversation* OR AB conversation* OR TI "clinical recognition" OR AB "clinical recognition" OR TI "clinical visibility" OR AB "clinical visibility" OR TI documentation OR AB documentation OR TI referral OR AB referral OR TI "follow-up care" OR AB "follow-up care") |
| CINAHL via EBSCO Research Platform | P2A: GenPs-specific or genital-site-specific instrument search. | To identify genital psoriasis-specific or genital-site-specific instrument names, endpoint names, study-specific measurement paths, trial outcome terms, and source reports. | (MH "Psoriasis+" OR TI psoriasis OR AB psoriasis OR TI "genital psoriasis" OR AB "genital psoriasis" OR TI "anogenital psoriasis" OR AB "anogenital psoriasis" OR TI "perianal psoriasis" OR AB "perianal psoriasis") AND (TI "sPGA-G" OR AB "sPGA-G" OR TI "static Physician's Global Assessment of Genitalia" OR AB "static Physician's Global Assessment of Genitalia" OR TI "static Physician Global Assessment of Genitalia" OR AB "static Physician Global Assessment of Genitalia" OR TI "genital PGA" OR AB "genital PGA" OR TI "genital Physician's Global Assessment" OR AB "genital Physician's Global Assessment" OR TI "genital Physician Global Assessment" OR AB "genital Physician Global Assessment" OR TI "physician global assessment of genital psoriasis" OR AB "physician global assessment of genital psoriasis" OR TI "Patient Global Assessment-Genital" OR AB "Patient Global Assessment-Genital" OR TI "Patient's Global Assessment of Genital Psoriasis" OR AB "Patient's Global Assessment of Genital Psoriasis" OR TI "Patient Global Assessment of Genital Psoriasis" OR AB "Patient Global Assessment of Genital Psoriasis" OR TI "PatGA-Genital" OR AB "PatGA-Genital" OR TI mGPASI OR AB mGPASI OR TI "modified Genital Psoriasis Area and Severity Index" OR AB "modified Genital Psoriasis Area and Severity Index" OR TI "modified genital PASI" OR AB "modified genital PASI" OR TI GPSS OR AB GPSS OR TI "Genital Psoriasis Symptoms Scale" OR AB "Genital Psoriasis Symptoms Scale" OR TI "GPI-NRS" OR AB "GPI-NRS" OR TI "Genital Psoriasis Itch Numeric Rating Scale" OR AB "Genital Psoriasis Itch Numeric Rating Scale" OR TI "genital itch Numeric Rating Scale" OR AB "genital itch Numeric Rating Scale" OR TI "genital itch NRS" OR AB "genital itch NRS" OR TI "GenPs-Itch NRS" OR AB "GenPs-Itch NRS" OR TI "GenPs-SFQ" OR AB "GenPs-SFQ" OR TI "Genital Psoriasis Sexual Frequency Questionnaire" OR AB "Genital Psoriasis Sexual Frequency Questionnaire" OR TI "Sexual Frequency Questionnaire" OR AB "Sexual Frequency Questionnaire" OR TI GPSIS OR AB GPSIS OR TI "Genital Psoriasis Sexual Impact Scale" OR AB "Genital Psoriasis Sexual Impact Scale" OR TI "Reasons for Sexual Impairment" OR AB "Reasons for Sexual Impairment" OR TI "reasons for sexual impairment" OR AB "reasons for sexual impairment" OR ((TI RSI OR AB RSI) AND (TI psoriasis OR AB psoriasis) AND (TI "sexual impairment" OR AB "sexual impairment" OR TI "sexual impact" OR AB "sexual impact" OR TI "sexual activity" OR AB "sexual activity" OR TI "sexual avoidance" OR AB "sexual avoidance"))) |
| CINAHL via EBSCO Research Platform | P2B: GenPs-specific or genital-site-specific instruments plus validation and psychometric terms. | To identify evidence on the development, validation, content validity, reliability, responsiveness, score interpretation, and psychometric status of genital psoriasis-specific or genital-site-specific instruments. | (MH "Psoriasis+" OR TI psoriasis OR AB psoriasis OR TI "genital psoriasis" OR AB "genital psoriasis" OR TI "anogenital psoriasis" OR AB "anogenital psoriasis" OR TI "perianal psoriasis" OR AB "perianal psoriasis") AND (TI "sPGA-G" OR AB "sPGA-G" OR TI "static Physician's Global Assessment of Genitalia" OR AB "static Physician's Global Assessment of Genitalia" OR TI "static Physician Global Assessment of Genitalia" OR AB "static Physician Global Assessment of Genitalia" OR TI "genital PGA" OR AB "genital PGA" OR TI "genital Physician's Global Assessment" OR AB "genital Physician's Global Assessment" OR TI "genital Physician Global Assessment" OR AB "genital Physician Global Assessment" OR TI "physician global assessment of genital psoriasis" OR AB "physician global assessment of genital psoriasis" OR TI "Patient Global Assessment-Genital" OR AB "Patient Global Assessment-Genital" OR TI "Patient's Global Assessment of Genital Psoriasis" OR AB "Patient's Global Assessment of Genital Psoriasis" OR TI "Patient Global Assessment of Genital Psoriasis" OR AB "Patient Global Assessment of Genital Psoriasis" OR TI "PatGA-Genital" OR AB "PatGA-Genital" OR TI mGPASI OR AB mGPASI OR TI "modified Genital Psoriasis Area and Severity Index" OR AB "modified Genital Psoriasis Area and Severity Index" OR TI "modified genital PASI" OR AB "modified genital PASI" OR TI GPSS OR AB GPSS OR TI "Genital Psoriasis Symptoms Scale" OR AB "Genital Psoriasis Symptoms Scale" OR TI "GPI-NRS" OR AB "GPI-NRS" OR TI "Genital Psoriasis Itch Numeric Rating Scale" OR AB "Genital Psoriasis Itch Numeric Rating Scale" OR TI "genital itch Numeric Rating Scale" OR AB "genital itch Numeric Rating Scale" OR TI "genital itch NRS" OR AB "genital itch NRS" OR TI "GenPs-Itch NRS" OR AB "GenPs-Itch NRS" OR TI "GenPs-SFQ" OR AB "GenPs-SFQ" OR TI "Genital Psoriasis Sexual Frequency Questionnaire" OR AB "Genital Psoriasis Sexual Frequency Questionnaire" OR TI "Sexual Frequency Questionnaire" OR AB "Sexual Frequency Questionnaire" OR TI GPSIS OR AB GPSIS OR TI "Genital Psoriasis Sexual Impact Scale" OR AB "Genital Psoriasis Sexual Impact Scale" OR TI "Reasons for Sexual Impairment" OR AB "Reasons for Sexual Impairment" OR TI "reasons for sexual impairment" OR AB "reasons for sexual impairment" OR ((TI RSI OR AB RSI) AND (TI psoriasis OR AB psoriasis) AND (TI "sexual impairment" OR AB "sexual impairment" OR TI "sexual impact" OR AB "sexual impact" OR TI "sexual activity" OR AB "sexual activity" OR TI "sexual avoidance" OR AB "sexual avoidance"))) AND (TI psychometric* OR AB psychometric* OR TI validation OR AB validation OR TI validated OR AB validated OR TI validity OR AB validity OR TI valid* OR AB valid* OR TI reliability OR AB reliability OR TI reliable OR AB reliable OR TI responsiveness OR AB responsiveness OR TI responsive OR AB responsive OR TI "content validity" OR AB "content validity" OR TI "construct validity" OR AB "construct validity" OR TI "structural validity" OR AB "structural validity" OR TI "factor analysis" OR AB "factor analysis" OR TI "internal consistency" OR AB "internal consistency" OR TI "test-retest" OR AB "test-retest" OR TI "cognitive debriefing" OR AB "cognitive debriefing" OR TI "item generation" OR AB "item generation" OR TI "item reduction" OR AB "item reduction" OR TI "score interpretation" OR AB "score interpretation" OR TI interpretability OR AB interpretability OR TI "meaningful change" OR AB "meaningful change" OR TI "minimally important difference" OR AB "minimally important difference" OR TI "patient-reported outcome" OR AB "patient-reported outcome" OR TI "patient-reported outcomes" OR AB "patient-reported outcomes" OR TI "clinical outcome assessment" OR AB "clinical outcome assessment" OR TI "clinical outcome assessments" OR AB "clinical outcome assessments") |
| CINAHL via EBSCO Research Platform | P2C: Focused search for dermatology-, psoriasis-, skin-related sexuality, and generic comparator instruments. | To identify dermatology-related, psoriasis-related, skin-related sexuality, generic sexual function, sexual distress, genital self-image, intimacy, stigma, patient needs, and comparator instruments. | (MH "Psoriasis+" OR TI psoriasis OR AB psoriasis OR TI dermatolog* OR AB dermatolog* OR MH "Skin Diseases+" OR TI "skin disease" OR AB "skin disease" OR TI "skin diseases" OR AB "skin diseases" OR TI "chronic skin disease" OR AB "chronic skin disease" OR TI "chronic skin diseases" OR AB "chronic skin diseases" OR TI "visible skin disease" OR AB "visible skin disease" OR TI "visible skin diseases" OR AB "visible skin diseases" OR TI "cutaneous disease" OR AB "cutaneous disease" OR TI "cutaneous diseases" OR AB "cutaneous diseases" OR TI "vulvar dermatosis" OR AB "vulvar dermatosis" OR TI "vulvar dermatoses" OR AB "vulvar dermatoses" OR TI "genital dermatosis" OR AB "genital dermatosis" OR TI "genital dermatoses" OR AB "genital dermatoses") AND (TI "Female Sexual Function Index" OR AB "Female Sexual Function Index" OR TI FSFI OR AB FSFI OR TI "International Index of Erectile Function" OR AB "International Index of Erectile Function" OR TI IIEF OR AB IIEF OR TI "IIEF-5" OR AB "IIEF-5" OR TI "Arizona Sexual Experience Scale" OR AB "Arizona Sexual Experience Scale" OR TI "Arizona Sexual Experiences Scale" OR AB "Arizona Sexual Experiences Scale" OR TI ASEX OR AB ASEX OR TI "Massachusetts General Hospital Sexual Functioning Questionnaire" OR AB "Massachusetts General Hospital Sexual Functioning Questionnaire" OR TI "MGH-SFQ" OR AB "MGH-SFQ" OR TI "Female Sexual Distress Scale" OR AB "Female Sexual Distress Scale" OR TI "Female Sexual Distress Scale-Revised" OR AB "Female Sexual Distress Scale-Revised" OR TI FSDS OR AB FSDS OR TI "FSDS-R" OR AB "FSDS-R" OR TI QualipsoSex OR AB QualipsoSex OR TI Qualipsosex OR AB Qualipsosex OR TI "QualipsoSex questionnaire" OR AB "QualipsoSex questionnaire" OR TI "Qualipsosex questionnaire" OR AB "Qualipsosex questionnaire" OR TI "Skin-Related Sexual Life Questionnaire" OR AB "Skin-Related Sexual Life Questionnaire" OR TI "Skin Related Sexual Life Questionnaire" OR AB "Skin Related Sexual Life Questionnaire" OR TI SRSLQ OR AB SRSLQ OR TI "Sexual Quality of Life Questionnaire for Men" OR AB "Sexual Quality of Life Questionnaire for Men" OR TI "Sexual Quality of Life Questionnaire for use in Men" OR AB "Sexual Quality of Life Questionnaire for use in Men" OR TI "SQoL-M" OR AB "SQoL-M" OR TI "Female Genital Self-Image Scale" OR AB "Female Genital Self-Image Scale" OR TI FGSIS OR AB FGSIS OR TI "Dermatology Life Quality Index" OR AB "Dermatology Life Quality Index" OR TI DLQI OR AB DLQI OR TI "DLQI item 9" OR AB "DLQI item 9" OR TI "sexual difficulties item" OR AB "sexual difficulties item" OR TI "Skindex-16" OR AB "Skindex-16" OR TI "Vulval Quality of Life Index" OR AB "Vulval Quality of Life Index" OR TI "Vulvar Quality of Life Index" OR AB "Vulvar Quality of Life Index" OR TI VQLI OR AB VQLI OR TI VLQI OR AB VLQI OR TI "Psoriasis Disability Index" OR AB "Psoriasis Disability Index" OR TI PDI OR AB PDI OR TI "Psoriasis Internalized Stigma Scale" OR AB "Psoriasis Internalized Stigma Scale" OR TI PISS OR AB PISS OR TI "Relationship and Sexuality Scale" OR AB "Relationship and Sexuality Scale" OR TI "Revised Dyadic Adjustment Scale" OR AB "Revised Dyadic Adjustment Scale" OR TI "Dermatologic Intimacy Scale" OR AB "Dermatologic Intimacy Scale" OR TI "Perceived Stigmatization Questionnaire" OR AB "Perceived Stigmatization Questionnaire" OR TI "Dysmorphic Concern Questionnaire" OR AB "Dysmorphic Concern Questionnaire" OR TI "Patient Benefit Index" OR AB "Patient Benefit Index" OR TI PBI OR AB PBI OR TI "Patient Needs Questionnaire" OR AB "Patient Needs Questionnaire" OR TI "Patient Benefit Questionnaire" OR AB "Patient Benefit Questionnaire" OR TI "Comprehensive Assessment of Psoriasis-related Patient-reported outcomes" OR AB "Comprehensive Assessment of Psoriasis-related Patient-reported outcomes") AND (TI "sexual health" OR AB "sexual health" OR TI psychosexual OR AB psychosexual OR TI "sexual function" OR AB "sexual function" OR TI "sexual dysfunction" OR AB "sexual dysfunction" OR TI "sexual distress" OR AB "sexual distress" OR TI "sexual quality of life" OR AB "sexual quality of life" OR TI "skin-related sexuality" OR AB "skin-related sexuality" OR TI sexuality OR AB sexuality OR TI intimacy OR AB intimacy OR TI "body image" OR AB "body image" OR TI "genital self-image" OR AB "genital self-image" OR TI "sexual self-image" OR AB "sexual self-image" OR TI stigma* OR AB stigma* OR TI "self-stigma" OR AB "self-stigma" OR TI "internalized stigma" OR AB "internalized stigma" OR TI shame OR AB shame OR TI embarrassment OR AB embarrassment OR MH "Quality of Life+" OR TI "quality of life" OR AB "quality of life" OR TI QoL OR AB QoL OR TI psychometric* OR AB psychometric* OR TI valid* OR AB valid* OR TI validation OR AB validation OR TI reliability OR AB reliability OR TI responsiveness OR AB responsiveness OR TI "content validity" OR AB "content validity" OR TI "construct validity" OR AB "construct validity" OR TI "structural validity" OR AB "structural validity" OR TI "factor analysis" OR AB "factor analysis" OR TI "score interpretation" OR AB "score interpretation" OR TI interpretability OR AB interpretability OR TI "meaningful change" OR AB "meaningful change" OR TI "minimally important difference" OR AB "minimally important difference" OR TI "not relevant" OR AB "not relevant" OR TI "not applicable" OR AB "not applicable" OR TI "not sexually active" OR AB "not sexually active" OR TI "no sexual activity" OR AB "no sexual activity" OR TI "no partner" OR AB "no partner" OR TI "partner status" OR AB "partner status" OR TI "current sexual activity" OR AB "current sexual activity" OR TI "sexual orientation" OR AB "sexual orientation" OR TI "gender identity" OR AB "gender identity" OR TI transgender OR AB transgender OR TI "gender diverse" OR AB "gender diverse" OR TI "same-sex partnership" OR AB "same-sex partnership") |
| CINAHL via EBSCO Research Platform | P3A-2: Targeted construct-evidence search with measurement and interpretation terms. | To identify supplementary construct evidence that includes construct terms together with measurement, instruments, PROs, psychometrics, communication, clinical recognition, or patient needs. | (MH "Psoriasis+" OR TI psoriasis OR AB psoriasis OR TI dermatolog* OR AB dermatolog* OR MH "Skin Diseases+" OR TI "skin disease" OR AB "skin disease" OR TI "skin diseases" OR AB "skin diseases" OR TI "chronic skin disease" OR AB "chronic skin disease" OR TI "chronic skin diseases" OR AB "chronic skin diseases" OR TI "visible skin disease" OR AB "visible skin disease" OR TI "visible skin diseases" OR AB "visible skin diseases" OR TI "cutaneous disease" OR AB "cutaneous disease" OR TI "cutaneous diseases" OR AB "cutaneous diseases" OR TI "vulvar dermatosis" OR AB "vulvar dermatosis" OR TI "vulvar dermatoses" OR AB "vulvar dermatoses" OR TI "genital dermatosis" OR AB "genital dermatosis" OR TI "genital dermatoses" OR AB "genital dermatoses") AND (TI psychosexual OR AB psychosexual OR TI "sexual distress" OR AB "sexual distress" OR TI "sexual avoidance" OR AB "sexual avoidance" OR TI "sexual quality of life" OR AB "sexual quality of life" OR TI "sexual health" OR AB "sexual health" OR TI "sexual function" OR AB "sexual function" OR TI "sexual dysfunction" OR AB "sexual dysfunction" OR TI "sexual impairment" OR AB "sexual impairment" OR TI "sexual activity" OR AB "sexual activity" OR TI "sexual self-image" OR AB "sexual self-image" OR TI "genital self-image" OR AB "genital self-image" OR TI "body image" OR AB "body image" OR TI "appearance concern" OR AB "appearance concern" OR TI "appearance concerns" OR AB "appearance concerns" OR TI shame OR AB shame OR TI embarrassment OR AB embarrassment OR TI stigma OR AB stigma OR TI stigmatization OR AB stigmatization OR TI stigmatisation OR AB stigmatisation OR TI "self-stigma" OR AB "self-stigma" OR TI "internalized stigma" OR AB "internalized stigma" OR TI "internalised stigma" OR AB "internalised stigma" OR TI intimacy OR AB intimacy OR TI "intimate relationship" OR AB "intimate relationship" OR TI "intimate relationships" OR AB "intimate relationships" OR TI "relationship burden" OR AB "relationship burden" OR TI "partner evaluation" OR AB "partner evaluation" OR TI "fear of rejection" OR AB "fear of rejection" OR TI avoidance OR AB avoidance) AND (TI measurement OR AB measurement OR TI measure* OR AB measure* OR TI instrument* OR AB instrument* OR TI questionnaire* OR AB questionnaire* OR TI scale* OR AB scale* OR TI construct OR AB construct OR TI constructs OR AB constructs OR TI "patient-reported outcome" OR AB "patient-reported outcome" OR TI "patient-reported outcomes" OR AB "patient-reported outcomes" OR TI "patient reported outcome" OR AB "patient reported outcome" OR TI "patient reported outcomes" OR AB "patient reported outcomes" OR TI psychometric* OR AB psychometric* OR TI valid* OR AB valid* OR TI validation OR AB validation OR TI "score interpretation" OR AB "score interpretation" OR TI communication OR AB communication OR TI "clinical recognition" OR AB "clinical recognition" OR TI "clinical visibility" OR AB "clinical visibility" OR TI "patient needs" OR AB "patient needs" OR TI "unmet needs" OR AB "unmet needs") |
| CINAHL via EBSCO Research Platform | P3B-1-revised: Patient-needs and care-context search. | To identify evidence on patient needs, unmet needs, patient preferences, patient–clinician communication, clinical recognition, and clinical visibility in psoriasis, dermatology, or genital-dermatology literature. | (MH "Psoriasis+" OR TI psoriasis OR AB psoriasis OR TI dermatolog* OR AB dermatolog* OR MH "Skin Diseases+" OR TI "skin disease" OR AB "skin disease" OR TI "skin diseases" OR AB "skin diseases" OR TI "chronic skin disease" OR AB "chronic skin disease" OR TI "chronic skin diseases" OR AB "chronic skin diseases" OR TI "visible skin disease" OR AB "visible skin disease" OR TI "visible skin diseases" OR AB "visible skin diseases" OR TI "cutaneous disease" OR AB "cutaneous disease" OR TI "cutaneous diseases" OR AB "cutaneous diseases" OR TI "dermatologic disease" OR AB "dermatologic disease" OR TI "dermatologic diseases" OR AB "dermatologic diseases" OR TI "vulvar dermatosis" OR AB "vulvar dermatosis" OR TI "vulvar dermatoses" OR AB "vulvar dermatoses" OR TI "genital dermatosis" OR AB "genital dermatosis" OR TI "genital dermatoses" OR AB "genital dermatoses" OR ((TI genital OR AB genital OR TI anogenital OR AB anogenital OR TI perianal OR AB perianal OR TI vulvar OR AB vulvar OR TI vulval OR AB vulval) AND (TI dermatolog* OR AB dermatolog* OR TI dermatos* OR AB dermatos* OR TI cutaneous OR AB cutaneous OR TI skin OR AB skin))) AND (TI "patient needs" OR AB "patient needs" OR TI "unmet needs" OR AB "unmet needs" OR TI "patient need" OR AB "patient need" OR TI "unmet need" OR AB "unmet need" OR TI "patient preference" OR AB "patient preference" OR TI "patient preferences" OR AB "patient preferences" OR TI "Patient Benefit Index" OR AB "Patient Benefit Index" OR TI "Patient Needs Questionnaire" OR AB "Patient Needs Questionnaire" OR TI "Patient Benefit Questionnaire" OR AB "Patient Benefit Questionnaire" OR TI "patient-clinician communication" OR AB "patient-clinician communication" OR TI "clinician-patient communication" OR AB "clinician-patient communication" OR TI "physician-patient communication" OR AB "physician-patient communication" OR TI "doctor-patient communication" OR AB "doctor-patient communication" OR TI "difficult-to-communicate" OR AB "difficult-to-communicate" OR TI "preferred language" OR AB "preferred language" OR TI "dermatology practice" OR AB "dermatology practice" OR TI "dermatology visit" OR AB "dermatology visit" OR TI "dermatology visits" OR AB "dermatology visits" OR TI "routine dermatology" OR AB "routine dermatology" OR TI "outpatient dermatology" OR AB "outpatient dermatology" OR TI "clinical recognition" OR AB "clinical recognition" OR TI "clinical visibility" OR AB "clinical visibility") AND (TI genital OR AB genital OR TI genitalia OR AB genitalia OR TI anogenital OR AB anogenital OR TI perianal OR AB perianal OR TI vulvar OR AB vulvar OR TI vulval OR AB vulval OR TI sexual OR AB sexual OR TI psychosexual OR AB psychosexual OR TI "sexual health" OR AB "sexual health" OR TI "sexual function" OR AB "sexual function" OR TI "sexual distress" OR AB "sexual distress" OR TI "sexual avoidance" OR AB "sexual avoidance" OR TI intimacy OR AB intimacy OR TI "body image" OR AB "body image" OR TI "genital self-image" OR AB "genital self-image" OR TI stigma* OR AB stigma* OR TI shame OR AB shame OR TI embarrassment OR AB embarrassment OR MH "Quality of Life+" OR TI "quality of life" OR AB "quality of life" OR TI QoL OR AB QoL OR TI "patient-reported outcome" OR AB "patient-reported outcome" OR TI "patient-reported outcomes" OR AB "patient-reported outcomes" OR TI communication OR AB communication OR TI "clinical recognition" OR AB "clinical recognition" OR TI "clinical visibility" OR AB "clinical visibility") |
| CINAHL via EBSCO Research Platform | P3A-1: Broad supplementary construct search. | To identify supplementary construct evidence related to sexual function versus sexual health, sexual distress, sexual avoidance, body image, genital self-image, sexual self-image, shame, stigma, intimacy, and relationship burden. | (MH "Psoriasis+" OR TI psoriasis OR AB psoriasis OR TI dermatolog* OR AB dermatolog* OR MH "Skin Diseases+" OR TI "skin disease" OR AB "skin disease" OR TI "skin diseases" OR AB "skin diseases" OR TI "chronic skin disease" OR AB "chronic skin disease" OR TI "chronic skin diseases" OR AB "chronic skin diseases" OR TI "visible skin disease" OR AB "visible skin disease" OR TI "visible skin diseases" OR AB "visible skin diseases" OR TI "cutaneous disease" OR AB "cutaneous disease" OR TI "cutaneous diseases" OR AB "cutaneous diseases" OR TI "vulvar dermatosis" OR AB "vulvar dermatosis" OR TI "vulvar dermatoses" OR AB "vulvar dermatoses" OR TI "genital dermatosis" OR AB "genital dermatosis" OR TI "genital dermatoses" OR AB "genital dermatoses") AND (TI psychosexual OR AB psychosexual OR TI "sexual distress" OR AB "sexual distress" OR TI "sexual avoidance" OR AB "sexual avoidance" OR TI "sexual quality of life" OR AB "sexual quality of life" OR TI "sexual health" OR AB "sexual health" OR TI "sexual function" OR AB "sexual function" OR TI "sexual dysfunction" OR AB "sexual dysfunction" OR TI "sexual impairment" OR AB "sexual impairment" OR TI "sexual activity" OR AB "sexual activity" OR TI "sexual self-image" OR AB "sexual self-image" OR TI "genital self-image" OR AB "genital self-image" OR TI "body image" OR AB "body image" OR TI "appearance concern" OR AB "appearance concern" OR TI "appearance concerns" OR AB "appearance concerns" OR TI shame OR AB shame OR TI embarrassment OR AB embarrassment OR TI stigma OR AB stigma OR TI stigmatization OR AB stigmatization OR TI stigmatisation OR AB stigmatisation OR TI "self-stigma" OR AB "self-stigma" OR TI "internalized stigma" OR AB "internalized stigma" OR TI "internalised stigma" OR AB "internalised stigma" OR TI intimacy OR AB intimacy OR TI "intimate relationship" OR AB "intimate relationship" OR TI "intimate relationships" OR AB "intimate relationships" OR TI "relationship burden" OR AB "relationship burden" OR TI "partner evaluation" OR AB "partner evaluation" OR TI "fear of rejection" OR AB "fear of rejection" OR TI avoidance OR AB avoidance) |
| CINAHL via EBSCO Research Platform | P3B-2: Sexual-health and genital-sensitive communication, and clinical-recognition search. | To identify evidence related to sexual-health or genital-sensitive communication, clinical recognition, documentation, referral, and follow-up care. | (MH "Psoriasis+" OR TI psoriasis OR AB psoriasis OR TI dermatolog* OR AB dermatolog* OR MH "Skin Diseases+" OR TI "skin disease" OR AB "skin disease" OR TI "skin diseases" OR AB "skin diseases" OR TI "chronic skin disease" OR AB "chronic skin disease" OR TI "chronic skin diseases" OR AB "chronic skin diseases" OR TI "visible skin disease" OR AB "visible skin disease" OR TI "visible skin diseases" OR AB "visible skin diseases") AND (TI "sexual health discussion" OR AB "sexual health discussion" OR TI "sexual health communication" OR AB "sexual health communication" OR TI "sexual communication" OR AB "sexual communication" OR TI "patient-clinician communication" OR AB "patient-clinician communication" OR TI "clinician-patient communication" OR AB "clinician-patient communication" OR TI "physician-patient communication" OR AB "physician-patient communication" OR TI "doctor-patient communication" OR AB "doctor-patient communication" OR TI "sensitive topic" OR AB "sensitive topic" OR TI "sensitive topics" OR AB "sensitive topics" OR TI "sensitive issue" OR AB "sensitive issue" OR TI "sensitive issues" OR AB "sensitive issues" OR TI "difficult-to-communicate" OR AB "difficult-to-communicate" OR TI "preferred language" OR AB "preferred language" OR TI disclosure OR AB disclosure OR TI disclose* OR AB disclose* OR TI discuss* OR AB discuss* OR TI conversation* OR AB conversation* OR TI "clinical recognition" OR AB "clinical recognition" OR TI "clinical visibility" OR AB "clinical visibility" OR TI documentation OR AB documentation OR TI "medical record" OR AB "medical record" OR TI "medical records" OR AB "medical records" OR TI referral OR AB referral OR TI "follow-up care" OR AB "follow-up care" OR TI triage OR AB triage OR TI "initial identification" OR AB "initial identification") AND (TI genital OR AB genital OR TI genitalia OR AB genitalia OR TI "genital psoriasis" OR AB "genital psoriasis" OR TI "genital involvement" OR AB "genital involvement" OR TI anogenital OR AB anogenital OR TI perianal OR AB perianal OR TI vulvar OR AB vulvar OR TI vulval OR AB vulval OR TI psychosexual OR AB psychosexual OR TI "sexual health" OR AB "sexual health" OR TI "sexual function" OR AB "sexual function" OR TI "sexual dysfunction" OR AB "sexual dysfunction" OR TI "sexual distress" OR AB "sexual distress" OR TI "sexual avoidance" OR AB "sexual avoidance" OR TI "sexual activity" OR AB "sexual activity" OR TI "sexual quality of life" OR AB "sexual quality of life" OR TI intimacy OR AB intimacy OR TI "intimate relationship" OR AB "intimate relationship" OR TI "intimate relationships" OR AB "intimate relationships" OR TI "genital self-image" OR AB "genital self-image" OR TI "sexual self-image" OR AB "sexual self-image" OR ((TI "body image" OR AB "body image" OR TI stigma* OR AB stigma* OR TI shame OR AB shame OR TI embarrassment OR AB embarrassment) AND (TI sexual OR AB sexual OR TI genital OR AB genital OR TI intimacy OR AB intimacy OR TI psychosexual OR AB psychosexual))) |
| CINAHL via EBSCO Research Platform | P3C-1: DLQI “not relevant/not applicable” and score-interpretation search. | To identify literature on DLQI item 9, not relevant/not applicable responses, inapplicable items, scoring, interpretability, content relevance, and score interpretation. | (TI "Dermatology Life Quality Index" OR AB "Dermatology Life Quality Index" OR TI DLQI OR AB DLQI) AND (TI psoriasis OR AB psoriasis OR TI dermatolog* OR AB dermatolog* OR TI "skin disease" OR AB "skin disease" OR TI "skin diseases" OR AB "skin diseases" OR MH "Quality of Life+" OR TI "quality of life" OR AB "quality of life") AND (TI "not relevant" OR AB "not relevant" OR TI "not applicable" OR AB "not applicable" OR TI inapplicable OR AB inapplicable OR TI "response option" OR AB "response option" OR TI scoring OR AB scoring OR TI "score interpretation" OR AB "score interpretation" OR TI interpretability OR AB interpretability OR TI "content validity" OR AB "content validity" OR TI "content relevance" OR AB "content relevance" OR TI "missing data" OR AB "missing data") |
| CINAHL via EBSCO Research Platform | P3C-2: Applicability and inapplicable-item search for sexual function scales. | To identify literature on inapplicable items, not sexually active status, no partner, response options, missing data, floor effects, sexual orientation, and gender-identity applicability in sexual function, sexual distress, and sexual QoL scales. | (TI "Female Sexual Function Index" OR AB "Female Sexual Function Index" OR TI FSFI OR AB FSFI OR TI "International Index of Erectile Function" OR AB "International Index of Erectile Function" OR TI IIEF OR AB IIEF OR TI "IIEF-5" OR AB "IIEF-5" OR TI "Arizona Sexual Experience Scale" OR AB "Arizona Sexual Experience Scale" OR TI ASEX OR AB ASEX OR TI "Massachusetts General Hospital Sexual Functioning Questionnaire" OR AB "Massachusetts General Hospital Sexual Functioning Questionnaire" OR TI "MGH-SFQ" OR AB "MGH-SFQ" OR TI "Female Sexual Distress Scale" OR AB "Female Sexual Distress Scale" OR TI FSDS OR AB FSDS OR TI "FSDS-R" OR AB "FSDS-R" OR TI "Sexual Quality of Life Questionnaire for Men" OR AB "Sexual Quality of Life Questionnaire for Men" OR TI "SQoL-M" OR AB "SQoL-M") AND (TI "not applicable" OR AB "not applicable" OR TI "not relevant" OR AB "not relevant" OR TI inapplicable OR AB inapplicable OR TI "response option" OR AB "response option" OR TI "not sexually active" OR AB "not sexually active" OR TI "no sexual activity" OR AB "no sexual activity" OR TI "no partner" OR AB "no partner" OR TI "partner status" OR AB "partner status" OR TI "current sexual activity" OR AB "current sexual activity" OR TI "missing data" OR AB "missing data" OR TI "floor effect" OR AB "floor effect" OR TI heterosexual OR AB heterosexual OR TI "penetrative activity" OR AB "penetrative activity" OR TI "sexual orientation" OR AB "sexual orientation" OR TI "gender identity" OR AB "gender identity") AND (TI validity OR AB validity OR TI psychometric* OR AB psychometric* OR TI "content validity" OR AB "content validity" OR TI "content relevance" OR AB "content relevance" OR TI "score interpretation" OR AB "score interpretation" OR TI scoring OR AB scoring OR TI interpretability OR AB interpretability) |
| CINAHL via EBSCO Research Platform | P3C-3: Validity-theory and construct-misalignment search. | To identify methodological evidence on validity theory, construct underrepresentation, construct-irrelevant variance, and construct misalignment. | (TI "construct underrepresentation" OR AB "construct underrepresentation" OR TI "construct-irrelevant variance" OR AB "construct-irrelevant variance" OR TI "construct irrelevant variance" OR AB "construct irrelevant variance" OR TI "validity theory" OR AB "validity theory" OR ((TI "construct validity" OR AB "construct validity") AND (TI underrepresentation OR AB underrepresentation OR TI "irrelevant variance" OR AB "irrelevant variance"))) AND (TI "patient-reported outcome" OR AB "patient-reported outcome" OR TI "patient-reported outcomes" OR AB "patient-reported outcomes" OR TI "patient reported outcome" OR AB "patient reported outcome" OR TI "patient reported outcomes" OR AB "patient reported outcomes" OR TI "clinical outcome assessment" OR AB "clinical outcome assessment" OR TI "clinical outcome assessments" OR AB "clinical outcome assessments" OR TI questionnaire* OR AB questionnaire* OR TI scale* OR AB scale* OR TI instrument* OR AB instrument* OR TI measurement OR AB measurement OR TI psychometric* OR AB psychometric*) |
| CINAHL via EBSCO Research Platform | SOGI/LGBTQ+ applicability and underrepresentation supplementary search. | To identify evidence on SOGI/LGBTQ+ population applicability, communication needs, underrepresentation, and measurement boundaries in psoriasis, dermatology, or genital-dermatology literature. | (TI psoriasis OR AB psoriasis OR TI dermatolog* OR AB dermatolog* OR MH "Skin Diseases+" OR TI "skin disease" OR AB "skin disease" OR TI "skin diseases" OR AB "skin diseases" OR TI "chronic skin disease" OR AB "chronic skin disease" OR TI "chronic skin diseases" OR AB "chronic skin diseases" OR TI "visible skin disease" OR AB "visible skin disease" OR TI "visible skin diseases" OR AB "visible skin diseases" OR TI "cutaneous disease" OR AB "cutaneous disease" OR TI "cutaneous diseases" OR AB "cutaneous diseases" OR TI "vulvar dermatosis" OR AB "vulvar dermatosis" OR TI "vulvar dermatoses" OR AB "vulvar dermatoses" OR TI "genital dermatosis" OR AB "genital dermatosis" OR TI "genital dermatoses" OR AB "genital dermatoses") AND (TI LGBT* OR AB LGBT* OR TI LGBTQ* OR AB LGBTQ* OR TI "sexual minority" OR AB "sexual minority" OR TI "sexual minorities" OR AB "sexual minorities" OR TI "gender minority" OR AB "gender minority" OR TI "gender minorities" OR AB "gender minorities" OR TI "sexual orientation" OR AB "sexual orientation" OR TI "gender identity" OR AB "gender identity" OR TI transgender OR AB transgender OR TI nonbinary OR AB nonbinary OR TI "non-binary" OR AB "non-binary" OR TI "gender diverse" OR AB "gender diverse" OR TI "same-sex partner" OR AB "same-sex partner" OR TI "same-sex partnership" OR AB "same-sex partnership") AND (TI "sexual health" OR AB "sexual health" OR TI "sexual distress" OR AB "sexual distress" OR TI "sexual function" OR AB "sexual function" OR TI "body image" OR AB "body image" OR TI "genital self-image" OR AB "genital self-image" OR TI stigma OR AB stigma OR TI communication OR AB communication OR TI "patient-reported outcome" OR AB "patient-reported outcome" OR TI "patient-reported outcomes" OR AB "patient-reported outcomes" OR TI "clinical outcome assessment" OR AB "clinical outcome assessment" OR TI measurement OR AB measurement OR TI validity OR AB validity OR TI applicability OR AB applicability OR TI underrepresentation OR AB underrepresentation OR TI "measurement boundary" OR AB "measurement boundary" OR TI "measurement boundaries" OR AB "measurement boundaries") |
| CINAHL via EBSCO Research Platform | Search implementation summary |  | Main search: P1A OR P1B OR P2A OR P2B OR P2C OR P3A-2 OR P3B-1-revised.  Targeted supplementary searches: P3A-1, P3B-2, P3C-1, P3C-2, P3C-3, and the SOGI/LGBTQ+ applicability/underrepresentation search were run and recorded separately and were not included in the main search set. |
