# Supplementary material for: Psychosexual burden in adult genital psoriasis: outcome measurement, construct coverage, and clinical recognition gaps
Source: Front Psychiatry. 2026 Aug 12;17:1890558. doi: 10.3389/fpsyt.2026.1890558 (PMC13507888; doi:10.3389/fpsyt.2026.1890558)
Supplement: Supplementary file 3 [file Table2.docx]

**Supplementary Table 2. Eligibility scope and evidence-use boundaries**

| Item | Eligibility scope | Evidence-use boundaries |
| --- | --- | --- |
| Study population | Studies of adults (≥18 years) were included. Mixed adult–adolescent studies were included when they informed measurement, construct interpretation, or clinical recognition related to adult genital psoriasis. | Child- or adolescent-only studies were generally not used as evidence sources. |
| Disease and site | Studies were included when they involved genital, anogenital, perianal, or other relevant special-site psoriasis, including vulvar/vulval, penile, scrotal, perineal, intergluteal, or inguinal crease involvement. Inverse, intertriginous, or flexural psoriasis studies were included only when these sites were specified. | Psoriasis literature without genital or relevant special-site involvement was not used as direct evidence for adult genital psoriasis. |
| Outcomes and constructs | Literature was included when it addressed local symptoms, psychosexual burden, sexual health, sexual function, sexual distress, sexual avoidance, intimacy-related impact, genital or sexual self-image, body image, stigma, patient needs, communication, clinical recognition, PROs, COAs, or measurement evidence. | Literature on general efficacy, safety, or nonsite-specific disease burden was not used when it did not support the review focus. |
| Instruments and measurement evidence | Literature was included when it addressed instrument development, validation status, measurement content, score interpretation, instrument application, endpoint selection, outcome definition, or PRO/COA evidence. | AGP-related instruments were used for disease- or site-related measurement. Non-AGP-specific instruments were used only for construct reference, comparator evidence, or applicability-boundary discussion. |
| Instrument applicability and representativeness boundaries | Literature was included when it informed applicability limits of instruments assessing sexual function, sexual distress, sexual quality of life, genital self-image, intimacy, or related constructs. Relevant factors included partner status, recent or absent sexual activity, not applicable/not relevant items, scoring approaches, sexual orientation, gender identity, and SOGI/LGBTQ+-related applicability limits. | These factors were used to interpret applicability and representativeness limits, not as stand-alone exclusion criteria. |
| Supplementary construct and methodological evidence | Psoriasis, dermatology, sexual medicine, psychometrics, PRO, communication, validity theory, or SOGI/LGBTQ+-related literature was included as supplementary evidence when it informed construct interpretation, instrument boundaries, score interpretation, underrepresentation, or methodological context. | Supplementary evidence was used only for interpretation or methodological context. It was not used as direct evidence of adult genital psoriasis burden. |
| Communication and clinical recognition evidence | Literature was included when it addressed patient needs, patient–clinician communication, sensitive-topic communication, clinical recognition, clinical visibility, documentation, referral, follow-up care, or questionnaire response versus spontaneous disclosure. | Non-AGP literature was used only to explain communication, disclosure, documentation, recognition, or linkage to follow-up care. |
| Publication type and full-text availability | Original studies, instrument studies, clinical studies, supplementary reports or materials, consensus statements, or reviews were included when they provided extractable information relevant to this review. English full-text availability was assessed during screening. | Non-original studies were mainly used for background, definitions, instrument sources, endpoint information, or methodological context. Records without extractable key information were not used as main-text evidence. |
| Duplicate records and related reports | Duplicate records were merged. Related reports from the same study were considered together when they provided additional information. | Related reports were used to supplement information on instruments, outcomes, endpoints, scoring, or validation status. They were not counted as independent evidence records when they reported the same study population or study family. |
| Abbreviations: AGP, adult genital psoriasis; COA, clinical outcome assessment; PRO, patient-reported outcome; QoL, quality of life; SOGI, sexual orientation and gender identity; LGBTQ+, lesbian, gay, bisexual, transgender, queer or questioning, and related sexual and gender minority groups. | | |
