# Supplementary material for: Psychosexual burden in adult genital psoriasis: outcome measurement, construct coverage, and clinical recognition gaps
Source: Front Psychiatry. 2026 Aug 12;17:1890558. doi: 10.3389/fpsyt.2026.1890558 (PMC13507888; doi:10.3389/fpsyt.2026.1890558)
Supplement: Supplementary file 4 [file Table3.docx]

**Supplementary Table 3. Measurement tools and pathways: development, validation status, and applicability boundaries**

| Tool / pathway | Content validity / development | Structural validity | Reliability | Responsiveness | External validation / cross-population applicability | Scoring and applicability boundaries | Key sources |
| --- | --- | --- | --- | --- | --- | --- | --- |
| sPGA-G / genital PGA | GenPs-specific ClinRO for overall genital severity | Single global rating; not applicable | Source evidence; some inter-rater reliability data | Treatment-response endpoint in GenPs trials | Mainly GenPs treatment studies; limited routine-practice data | Local lesion severity only | Merola 2017 (1)  Ryan 2018 (2)  Guenther 2020 (3)  Merola 2024 (4) |
| PASI / PGA | Generic psoriasis severity tools | PASI formative index; PGA global rating | Established generic severity instruments | Overall psoriasis severity change | Widely used in psoriasis; not AGP-specific | Background disease-status reference only | Fredriksson, 1978(5)  da Silva 2020b (6)  Elsadek 2024 (7)  Ong 2025 (8) |
| mGPASI | GenPs regional severity adaptation | Regional area / severity index | Independent reliability data unclear | Regional severity change in GenPs studies | Mainly trial / observational contexts | Local lesion extent and severity only | Ryan 2018 (2)  Guenther 2020 (3) |
| PatGA-Genital | Patient global rating used in GenPs screening and later trials | Single patient global rating | Independent reliability data unclear | Patient-rated severity change | Mainly GenPs clinical studies | Patient-perceived severity only | Ryan 2018 (2)  Gottlieb et al., 2018a(9)  Merola et al., 2020(10)  Guenther 2020 (3) |
| GPSS | Content development with literature review, expert input, patient interviews, cognitive interviews, and cultural adaptation | Eight symptom NRS items; total 0–80 | Partial psychometric data in supplementary materials | Symptom change; itch-response thresholds used | Development and clinical-study support; real-world use available | Local symptom score only | Gottlieb 2018 (9)  Yosipovitch 2018 (11)  Guenther 2020 (3)  Ben-Anaya 2025 (12)  Janke 2026 (13) |
| GPI-NRS / GenPs-Itch NRS | Single-item GenPs itch NRS | Single-item NRS; not applicable | Independent reliability data unclear | Itch-intensity change; ≥3-point / ≥4-point response thresholds used | Mainly clinical-trial contexts | Itch intensity only | Ryan 2018 (2)  Yosipovitch 2018 (11)  Guenther 2020 (3)  Merola 2024 (4) |
| GenPs-SFQ | Content development with literature review, expert input, patient interviews, and cognitive interviews | Two separate items; no total score | Further psychometric evaluation needed; item 2 validation data available | Sexual-activity limitation change, mainly item 2 | Limited to development and trial contexts; limited SOGI / inactive / unpartnered validation | Depends on recent sexual activity | Gottlieb 2018b (14)  Ryan 2018 (2)  Yosipovitch,2018(11)  Guenther 2020 (3) |
| GPSIS / sexual impact PROs | Development information available; sexual-activity avoidance / symptom impact | Partial validation data; independent structural data limited | Partial validation data; independent reliability data limited | Sexual-activity avoidance / impact change | GenPs trial and real-world use; limited cross-population validation | Sexual activity impact / avoidance only | Cather 2018(15)  Yosipovitch 2018 (11)  Ben-Anaya 2025 (12)  Janke 2026 (13) |
| RSI | Investigator-developed reasons list | Not reported | Not reported | Descriptive reasons in routine-care survey | GenPs / ASI routine-care use; no independent validation | Study-specific reasons list; not a validated scale | Janke 2026 (13) |
| FSFI / IIEF / IIEF-5 / ASEX | Generic sexual function / response instruments | Original structural-validity evidence; no AGP-specific validation | Mature reliability evidence in original contexts | Sexual-function research; limited AGP response interpretation | Sexual medicine use; not AGP-specific | Sensitive to sexual activity, partner status, SOGI / SGM context, non-penetrative activity, and inapplicable items | Rosen et al., 1997 (16)  Rosen et al., 1999 (17)  Rosen et al., 2000 (18)  McGahuey 2000 (19)  Rynja et al., 2010 (20)  Meeuwis 2011 (21)  Yule et al., 2011 (22)  Boehmer 2012 (23)  Forbes 2014 (24)  Kiss 2021 (25)  Yi 2022 (26)  Lynch 2023 (27)  Quintana 2024 (28)  Elsadek 2024 (7) |
| QualipsoSex / SRSLQ | Skin-related sexuality tools; QualipsoSex developed with focus groups, content analysis, and Delphi process | Reported structural-validity data | Reported reliability data | Skin-related sexuality research; limited AGP response data | Psoriasis / PsA / dermatology contexts; not GenPs-specific | Skin-related sexuality reference only | Esteve 2018(29)  Lespessailles 2021 (30)  Kędra 2023 (31) |
| SQoL-M | Male sexual QoL instrument developed in PE / ED | Unidimensional structure supported | Internal consistency and test–retest reliability reported | Male sexual QoL research | PE / ED male samples; not GenPs-specific | Male sexual QoL reference only | Abraham 2008 (32)  Meeuwis 2011 (21) |
| FSDS / FSDS-R | Female sexual distress instruments | Unidimensional structure supported | Internal consistency and test–retest reliability reported | Treatment sensitivity / discrimination reported | Female sexual distress; not AGP-specific | Female sexual distress reference only | Derogatis 2002 (33)  Derogatis 2008 (34)  Meeuwis 2011 (21)  Yi 2022 (26) |
| RSS | Relationship- / sexuality-related impact in genital / anogenital psoriasis studies | Independent structural data unclear | Independent reliability data unclear | Relationship / sexual-life impact; limited AGP response data | Genital / anogenital psoriasis applications; not GenPs-specific | Affected by partner relationship, recent sexual activity, and intimacy context | Ryan 2015 (35)  Yang 2018 (36)  da Silva 2022 (37)  Janke 2026 (13) |
| DIS | Dermatology intimacy instrument | Reported structural-validity data | Reported reliability data | Dermatologic intimacy impact; limited AGP response data | Dermatology context; no clear GenPs-specific validation | Skin-related intimacy reference only | Malakouti 2017 (38) |
| DLQI / DLQI item 9 | Generic dermatology QoL tool; item 9 covers sexual difficulties | Mature DLQI psychometric use; item 9 single item | Mature DLQI reliability; limited item-9 standalone interpretation | QoL and sexual-difficulty change in GenPs studies | Widely used in dermatology; not GenPs-specific | Single-item sexual-difficulty signal; “not relevant” scored as 0 may underestimate sexual-life-related impact | Finlay 1994 (39)  Rencz 2018 (40)  Barbieri 2019 (41)  Barbieri 2020 (42)  Rencz 2021 (43)  Meeuwis 2011 (21)  Yosipovitch 2018 (11)  Yi 2022 (26)  Merola 2024 (4)  Ong 2025 (8) |
| FGSIS / MGSIS | Female / male genital self-image instruments | FGSIS unidimensional; MGSIS male-instrument validation | Internal consistency reported | Genital self-image reference; limited AGP response data | Not AGP-specific; FGSIS has female genital psoriasis application; MGSIS original validation only | Sex-specific; not generalizable to all genders or SOGI groups | Herbenick 2010 (44)  Herbenick 2013 (45)  Rivera 2022 (46)  Elsadek 2024 (7) |
| PBI | Dermatology patient-defined outcome measure; PNQ / PBQ | Weighted patient-needs / benefit score | Validation and application evidence in dermatology / psoriasis | Patient-perceived treatment benefit | Dermatology, psoriasis, and anogenital psoriasis contexts; not GenPs-specific | Patient needs / perceived benefit only | Augustin 2009 (47)  Feuerhahn 2012 (48)  Radtke 2013 (49)  da Silva 2020a (50)  da Silva 2020b (6)  da Silva 2022 (37) |
| Note: This table is descriptive and is not a formal COSMIN quality assessment. Scoring and applicability boundaries relate to construct underrepresentation and construct-irrelevant variance. Abbreviations: AGP, adult genital psoriasis; ASI, areas of sexual interest; ASEX, Arizona Sexual Experience Scale; ClinRO, clinician-reported outcome; COSMIN, COnsensus-based Standards for the selection of health Measurement INstruments; DIS, Dermatologic Intimacy Scale; DLQI, Dermatology Life Quality Index; ED, erectile dysfunction; FGSIS, Female Genital Self-Image Scale; FSDS, Female Sexual Distress Scale; FSDS-R, Female Sexual Distress Scale-Revised; FSFI, Female Sexual Function Index; GenPs, genital psoriasis; GenPs-Itch NRS, Genital Psoriasis Itch Numeric Rating Scale; GenPs-SFQ, Genital Psoriasis Sexual Frequency Questionnaire; GPI-NRS, Genital Psoriasis Itch Numeric Rating Scale; GPSIS, Genital Psoriasis Sexual Impact Scale; GPSS, Genital Psoriasis Symptoms Scale; IIEF, International Index of Erectile Function; IIEF-5, five-item International Index of Erectile Function; MGSIS, Male Genital Self-Image Scale; mGPASI, modified Genital Psoriasis Area and Severity Index; NRS, numeric rating scale; PASI, Psoriasis Area and Severity Index; PatGA-Genital, Patient Global Assessment of Genital Psoriasis; PBQ, Patient Benefit Questionnaire; PBI, Patient Benefit Index; PE, premature ejaculation; PGA, Physician’s Global Assessment; PNQ, Patient Needs Questionnaire; PRO, patient-reported outcome; PROs, patient-reported outcomes; PsA, psoriatic arthritis; QoL, quality of life; RSI, Reasons for Sexual Impairment questionnaire; RSS, Relationship and Sexuality Scale; SGM, sexual and gender minority; SOGI, sexual orientation and gender identity; sPGA-G, static Physician’s Global Assessment of Genitalia; SQoL-M, Sexual Quality of Life Questionnaire for Men; SRSLQ, Skin-Related Sexual Life Questionnaire. | | | | | | | |

References

1.	Merola JF, Bleakman AP, Gottlieb AB, Menter A, Naegeli AN, Bissonnette R, Guenther L, Sullivan J, Meeuwis K, See K, et al. The Static Physician’s Global Assessment of Genitalia: A Clinical Outcome Measure for the Severity of Genital Psoriasis. *J Drugs Dermatol* (2017) 16:793–799. https://pm.yuntsg.com/details.html?pmid=28809994 [Accessed May 2, 2026]

2.	Ryan C, Menter A, Guenther L, Blauvelt A, Bissonnette R, Meeuwis K, Sullivan J, Cather JC, Yosipovitch G, Gottlieb AB, et al. Efficacy and safety of ixekizumab in a randomized, double-blinded, placebo-controlled phase IIIb study of patients with moderate-to-severe genital psoriasis. *Br J Dermatol* (2018) 179:844–852. doi: 10.1111/bjd.16736

3.	Guenther L, Potts Bleakman A, Weisman J, Poulin Y, Spelman L, Burge R, Erickson J, Todd K, Bertram CC, Ryan C. Ixekizumab Results in Persistent Clinical Improvement in Moderate-to-Severe Genital Psoriasis During a 52 Week, Randomized, Placebo-Controlled, Phase 3 Clinical Trial. *Acta Derm Venereol* (2020) 100:adv00006. doi: 10.2340/00015555-3353

4.	Merola JF, Parish LC, Guenther L, Lynde C, Lacour J-P, Staubach P, Cheng S, Paris M, Picard H, Deignan C, et al. Efficacy and safety of apremilast in patients with moderate-to-severe genital psoriasis: Results from DISCREET, a phase 3 randomized, double-blind, placebo-controlled trial. *J Am Acad Dermatol* (2024) 90:485–493. doi: 10.1016/j.jaad.2023.10.020

5.	Fredriksson T, Pettersson U. Severe psoriasis--oral therapy with a new retinoid. *Dermatologica* (1978) 157:238–244. doi: 10.1159/000250839

6.	da Silva N, von Stülpnagel C, Langenbruch A, Danckworth A, Augustin M, Sommer R. Disease burden and patient needs and benefits in anogenital psoriasis: developmental specificities for person-centred healthcare of emerging adults and adults. *J Eur Acad Dermatol Venereol* (2020) 34:1010–1018. doi: 10.1111/jdv.16076

7.	Elsadek HM, Ali MS, Elaidy AM. Impact of Genital and Non-Genital Psoriasis on Genital Self-Image, Quality of Life, and Sexual Dysfunction in Female Patients. *Dermatol Pract Concept* (2024) 14:e2024159. doi: 10.5826/dpc.1403a159

8.	Ong KY, Heng YK, Zhao X, Oon HH. Genital Psoriasis in Asians: Impact on Quality of Life and Sexual Health. *Exp Dermatol* (2025) 34:e70072. doi: 10.1111/exd.70072

9.	Gottlieb AB, Kirby B, Ryan C, Naegeli AN, Burge R, Potts Bleakman A, Anatchkova MD, Yosipovitch G. The Development of a Patient-Reported Outcome Measure for Assessment of Genital Psoriasis Symptoms: The Genital Psoriasis Symptoms Scale (GPSS). *Dermatol Ther (Heidelb)* (2018) 8:45–56. doi: 10.1007/s13555-017-0213-2

10.	Merola JF, Ghislain P-D, Dauendorffer JN, Potts Bleakman A, Brnabic AJM, Burge R, Riedl E. Ixekizumab improves secondary lesional signs, pain and sexual health in patients with moderate-to-severe genital psoriasis. *J Eur Acad Dermatol Venereol* (2020) 34:1257–1262. doi: 10.1111/jdv.16181

11.	Yosipovitch G, Foley P, Ryan C, Cather JC, Meeuwis KA, Burge R, Bleakman AP, Lin C-Y, Malatestinic W, Gottlieb A. Ixekizumab Improved Patient-Reported Genital Psoriasis Symptoms and Impact of Symptoms on Sexual Activity vs Placebo in a Randomized, Double-Blind Study. *J Sex Med* (2018) 15:1645–1652. doi: 10.1016/j.jsxm.2018.09.004

12.	Ben-Anaya N, Yip T, Sorbe C, Bickert T, Hockmann J, Bürkle C-P, Lohmann K, Schild M, Augustin M, von Kiedrowski R. Reduction of impairment of sexual activity in patients with genital psoriasis treated with risankizumab: interim results of a real-world study. *J Dermatolog Treat* (2025) 36:2585248. doi: 10.1080/09546634.2025.2585248

13.	Janke TM, Sorbe C, Sommer R, Bickert T, Mrowietz U, Gomis-Kleindienst S, Schild M, Lohmann K, Augustin M. Prevalence of Genital Psoriasis and Its Impact on Patients’ Sexual Life in Routine Care: Epidemiologic Survey in Germany. *Psoriasis (Auckl)* (2026) 16:516803. doi: 10.2147/PTT.S516803

14.	Gottlieb AB, Kirby B, Ryan C, Naegeli AN, Burge R, Potts Bleakman A, Anatchkova MD, Cather J. The Development of the Genital Psoriasis Sexual Frequency Questionnaire (GenPs-SFQ) to Assess the Impact of Genital Psoriasis on Sexual Health. *Dermatol Ther (Heidelb)* (2018) 8:33–44. doi: 10.1007/s13555-017-0212-3

15.	Cather J, Kirby B, Ryan C, Naegeli AN, Burge R, Potts Bleakman A, Anatchkova MD, Gottlieb AB. 039 The Development of a Patient Reported Outcome Measure to Assess Sexual Impacts of Genital Psoriasis: GPSIS. *J Sex Med* (2018) 15:S19. doi: 10.1016/j.jsxm.2017.11.058

16.	Rosen RC, Riley A, Wagner G, Osterloh IH, Kirkpatrick J, Mishra A. The international index of erectile function (IIEF): a multidimensional scale for assessment of erectile dysfunction. *Urology* (1997) 49:822–830. doi: 10.1016/s0090-4295(97)00238-0

17.	Rosen RC, Cappelleri JC, Smith MD, Lipsky J, Peña BM. Development and evaluation of an abridged, 5-item version of the International Index of Erectile Function (IIEF-5) as a diagnostic tool for erectile dysfunction. *Int J Impot Res* (1999) 11:319–326. doi: 10.1038/sj.ijir.3900472

18.	Rosen R, Brown C, Heiman J, Leiblum S, Meston C, Shabsigh R, Ferguson D, D’Agostino R. The Female Sexual Function Index (FSFI): a multidimensional self-report instrument for the assessment of female sexual function. *J Sex Marital Ther* (2000) 26:191–208. doi: 10.1080/009262300278597

19.	McGahuey CA, Gelenberg AJ, Laukes CA, Moreno FA, Delgado PL, McKnight KM, Manber R. The Arizona Sexual Experience Scale (ASEX): reliability and validity. *J Sex Marital Ther* (2000) 26:25–40. doi: 10.1080/009262300278623

20.	Rynja S, Bosch R, Kok E, Wouters G, de Kort L. IIEF-15: unsuitable for assessing erectile function of young men? *J Sex Med* (2010) 7:2825–2830. doi: 10.1111/j.1743-6109.2010.01847.x

21.	Meeuwis K a. P, de Hullu JA, van de Nieuwenhof HP, Evers AWM, Massuger LF a. G, van de Kerkhof PCM, van Rossum MM. Quality of life and sexual health in patients with genital psoriasis. *Br J Dermatol* (2011) 164:1247–1255. doi: 10.1111/j.1365-2133.2011.10249.x

22.	Yule M, Davison J, Brotto L. The International Index of Erectile Function: a methodological critique and suggestions for improvement. *J Sex Marital Ther* (2011) 37:255–269. doi: 10.1080/0092623X.2011.582431

23.	Boehmer U, Timm A, Ozonoff A, Potter J. Applying the Female Sexual Functioning Index to sexual minority women. *J Womens Health (Larchmt)* (2012) 21:401–409. doi: 10.1089/jwh.2011.3072

24.	Forbes MK, Baillie AJ, Schniering CA. Critical flaws in the Female Sexual Function Index and the international index of Erectile Function. *J Sex Res* (2014) 51:485–491. doi: 10.1080/00224499.2013.876607

25.	Kiss MJ, McDonagh LK, Sparks B, Hamp T, Morrison TG. Accurately Assessing Gay Men’s Erectile Functioning: A Critique of the International Index of Erectile Function (IIEF) Use with Gay Men. *J Sex Res* (2021) 58:589–598. doi: 10.1080/00224499.2020.1811195

26.	Yi OS, Huan KY, Har LC, Ali NM, Chiang TW. Genital Psoriasis: A Prospective, Observational, Single-Centre Study on Prevalence, Clinical Features, Risk Factors, and Its Impact on Quality of Life and Sexual Health. *Indian J Dermatol* (2022) 67:205. doi: 10.4103/ijd.ijd_754_21

27.	Lynch KA, Austria MD, Le T, Walters CB, Vickers A, Roche KL, Atkinson TM, Carlsson SV. Suggestions for modifications to the Female Sexual Function Index based on cognitive interviews with sexual and gender minority individuals and cisgender, heterosexual persons. *J Sex Med* (2023) 20:871–877. doi: 10.1093/jsxmed/qdad042

28.	Quintana GR, Ponce FP, Escudero-Pastén J, Santibáñez-Palma JF, Aguayo-Zuñiga CP, Carrasco-Portiño M, Cid-Aguayo M, González-Arias M, Ilabaca P, Jarpa-Schäcker R, et al. Analysis of the International Index of Erectile Function: psychometric evidence and measurement invariance across relationship status and age generations in a Chilean sample. *Sex Med* (2024) 12:qfae084. doi: 10.1093/sexmed/qfae084

29.	Esteve E, Maccari F, Delavierre D, Vicariot C, Charles B, Marty M, Lespessailles E. Preliminary development of a questionnaire assessing the impact of psoriasis and psoriatic arthritis on patient’s perception of sexuality. *Medicine (Baltimore)* (2018) 97:e12807. doi: 10.1097/MD.0000000000012807

30.	Lespessailles E, Mahé E, Reguiai Z, Begon E, Maccari F, Beneton N, Chaby G, Rosenberg C, Dernis E, Roux F, et al. Psychometric validation of a patient-reported outcome questionnaire (Qualipsosex) assessing the impact of psoriasis and psoriatic arthritis on patient perception of sexuality. *Medicine (Baltimore)* (2021) 100:e24168. doi: 10.1097/MD.0000000000024168

31.	Kędra K, Reich A. Skin-Related Sexual Life Questionnaire (SRSLQ): Creation and Validation of the Questionnaire. *Medicina (Kaunas)* (2023) 59:2023. doi: 10.3390/medicina59112023

32.	Abraham L, Symonds T, Morris MF. Psychometric validation of a sexual quality of life questionnaire for use in men with premature ejaculation or erectile dysfunction. *J Sex Med* (2008) 5:595–601. doi: 10.1111/j.1743-6109.2007.00749.x

33.	Derogatis LR, Rosen R, Leiblum S, Burnett A, Heiman J. The Female Sexual Distress Scale (FSDS): initial validation of a standardized scale for assessment of sexually related personal distress in women. *J Sex Marital Ther* (2002) 28:317–330. doi: 10.1080/00926230290001448

34.	Derogatis L, Clayton A, Lewis-D’Agostino D, Wunderlich G, Fu Y. Validation of the female sexual distress scale-revised for assessing distress in women with hypoactive sexual desire disorder. *J Sex Med* (2008) 5:357–364. doi: 10.1111/j.1743-6109.2007.00672.x

35.	Ryan C, Sadlier M, De Vol E, Patel M, Lloyd AA, Day A, Lally A, Kirby B, Menter A. Genital psoriasis is associated with significant impairment in quality of life and sexual functioning. *J Am Acad Dermatol* (2015) 72:978–983. doi: 10.1016/j.jaad.2015.02.1127

36.	Yang EJ, Beck KM, Sanchez IM, Koo J, Liao W. The impact of genital psoriasis on quality of life: a systematic review. *Psoriasis (Auckl)* (2018) 8:41–47. doi: 10.2147/PTT.S169389

37.	da Silva N, Augustin M, Hilbring C, Braren-von Stülpnagel CC, Sommer R. Psychological (co)morbidity in patients with psoriasis: the impact of pruritus and anogenital involvement on symptoms of depression and anxiety and on body dysmorphic concerns - a cross-sectional study. *BMJ Open* (2022) 12:e055477. doi: 10.1136/bmjopen-2021-055477

38.	Malakouti M, Brown GE, Leon A, Wang E, Naegeli AN, Edson-Heredia E, Levin E, Koo JYM. The dermatologic intimacy scale: quantitatively measuring the impact of skin disease on intimacy. *J Dermatolog Treat* (2017) 28:347–352. doi: 10.1080/09546634.2016.1252032

39.	Finlay AY, Khan GK. Dermatology Life Quality Index (DLQI)--a simple practical measure for routine clinical use. *Clin Exp Dermatol* (1994) 19:210–216. doi: 10.1111/j.1365-2230.1994.tb01167.x

40.	Rencz F, Poór AK, Péntek M, Holló P, Kárpáti S, Gulácsi L, Szegedi A, Remenyik É, Hidvégi B, Herszényi K, et al. A detailed analysis of “not relevant” responses on the DLQI in psoriasis: potential biases in treatment decisions. *J Eur Acad Dermatol Venereol* (2018) 32:783–790. doi: 10.1111/jdv.14676

41.	Barbieri JS, Gelfand JM. Influence of “Not Relevant” Responses on the Dermatology Life Quality Index (DLQI) for Patients With Psoriasis in the United States. *JAMA Dermatol* (2019) 155:743–745. doi: 10.1001/jamadermatol.2018.5655

42.	Barbieri JS, Shin DB, Syed MN, Takeshita J, Gelfand JM. Evaluation of the Frequency of “Not Relevant” Responses on the Dermatology Life Quality Index by Sociodemographic Characteristics of Patients With Psoriasis. *JAMA Dermatol* (2020) 156:446–450. doi: 10.1001/jamadermatol.2019.4659

43.	Rencz F, Mitev AZ, Szabó Á, Beretzky Z, Poór AK, Holló P, Wikonkál N, Sárdy M, Kárpáti S, Szegedi A, et al. A Rasch model analysis of two interpretations of “not relevant” responses on the Dermatology Life Quality Index (DLQI). *Qual Life Res* (2021) 30:2375–2386. doi: 10.1007/s11136-021-02803-7

44.	Herbenick D, Reece M. Development and validation of the female genital self-image scale. *J Sex Med* (2010) 7:1822–1830. doi: 10.1111/j.1743-6109.2010.01728.x

45.	Herbenick D, Schick V, Reece M, Sanders SA, Fortenberry JD. The development and validation of the Male Genital Self-Image Scale: results from a nationally representative probability sample of men in the United States. *J Sex Med* (2013) 10:1516–1525. doi: 10.1111/jsm.12124

46.	Rivera S, Flood A, Dykstra C, Herbenick D, DeMaria AL. Genital Self-Image, Sexual Function, and Quality of Life Among Individuals with Vulvar and Non-Vulvar Inflammatory Dermatoses. *Arch Sex Behav* (2022) 51:3965–3979. doi: 10.1007/s10508-022-02353-0

47.	Augustin M, Radtke MA, Zschocke I, Blome C, Behechtnejad J, Schäfer I, Reusch M, Mielke V, Rustenbach SJ. The patient benefit index: a novel approach in patient-defined outcomes measurement for skin diseases. *Arch Dermatol Res* (2009) 301:561–571. doi: 10.1007/s00403-009-0928-8

48.	Feuerhahn J, Blome C, Radtke M, Augustin M. Validation of the patient benefit index for the assessment of patient-relevant benefit in the treatment of psoriasis. *Arch Dermatol Res* (2012) 304:433–441. doi: 10.1007/s00403-012-1256-y

49.	Radtke MA, Schäfer I, Blome C, Augustin M. Patient benefit index (PBI) in the treatment of psoriasis--results of the National Care Study “PsoHealth.” *Eur J Dermatol* (2013) 23:212–217. doi: 10.1684/ejd.2013.1988

50.	da Silva N, Augustin M, Langenbruch A, Mrowietz U, Reich K, Thaçi D, Boehncke W-H, Kirsten N, Danckworth A, Sommer R. Sex-related impairment and patient needs/benefits in anogenital psoriasis: Difficult-to-communicate topics and their impact on patient-centred care. *PLoS One* (2020) 15:e0235091. doi: 10.1371/journal.pone.0235091
